# Supplementary figures and images for: Organisms with alternative genetic codes resolve unassigned codons via mistranslation and ribosomal rescue
Source: eLife. 2018 Oct 30;7:e34878. doi: 10.7554/eLife.34878 (PMC6207430; doi:10.7554/eLife.34878)

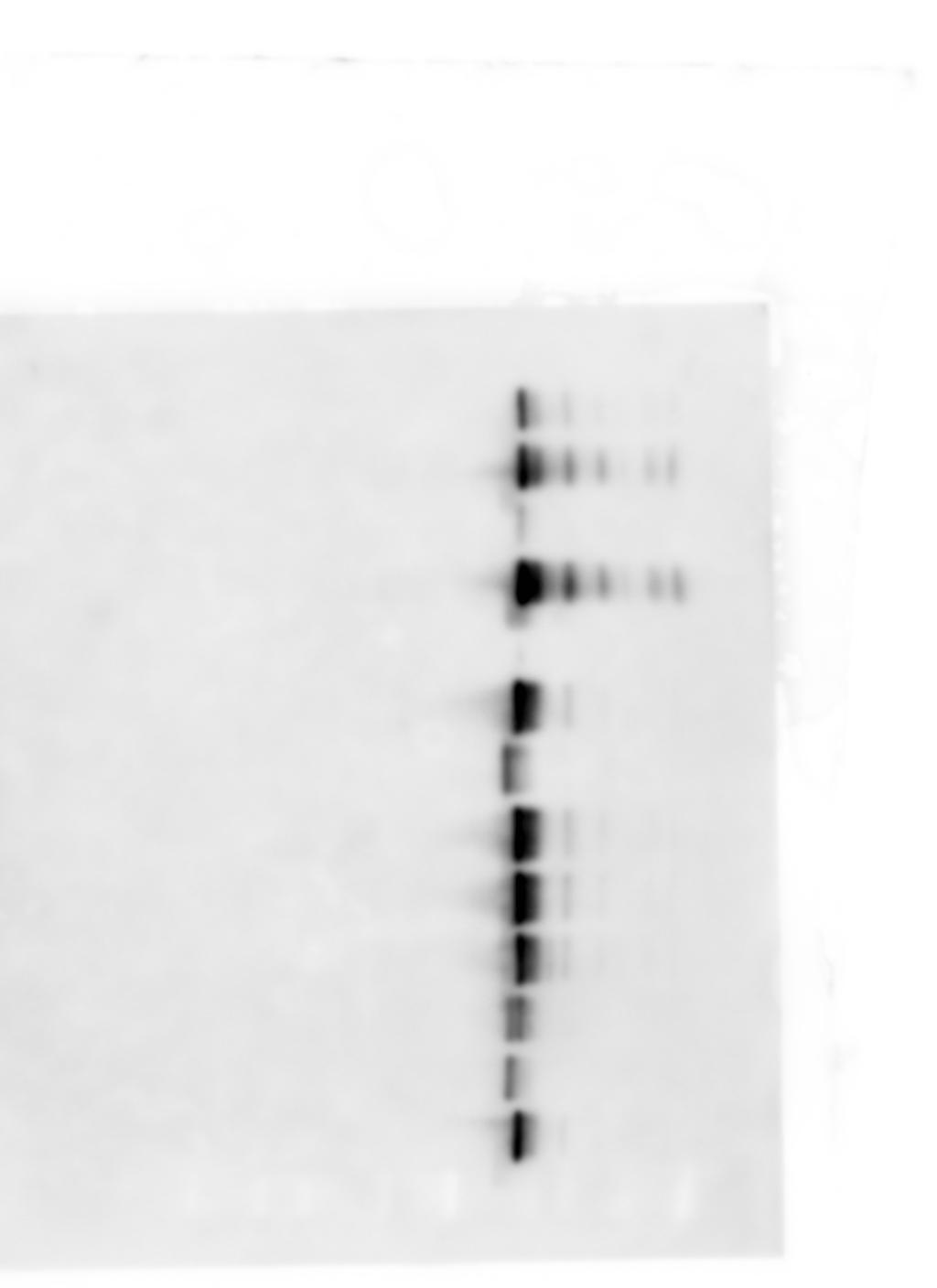

Supplement: Figure 3—source data 3. [file elife-34878-fig3-data3.zip › Figure 3 ΓÇô Source Data 3.jpg]

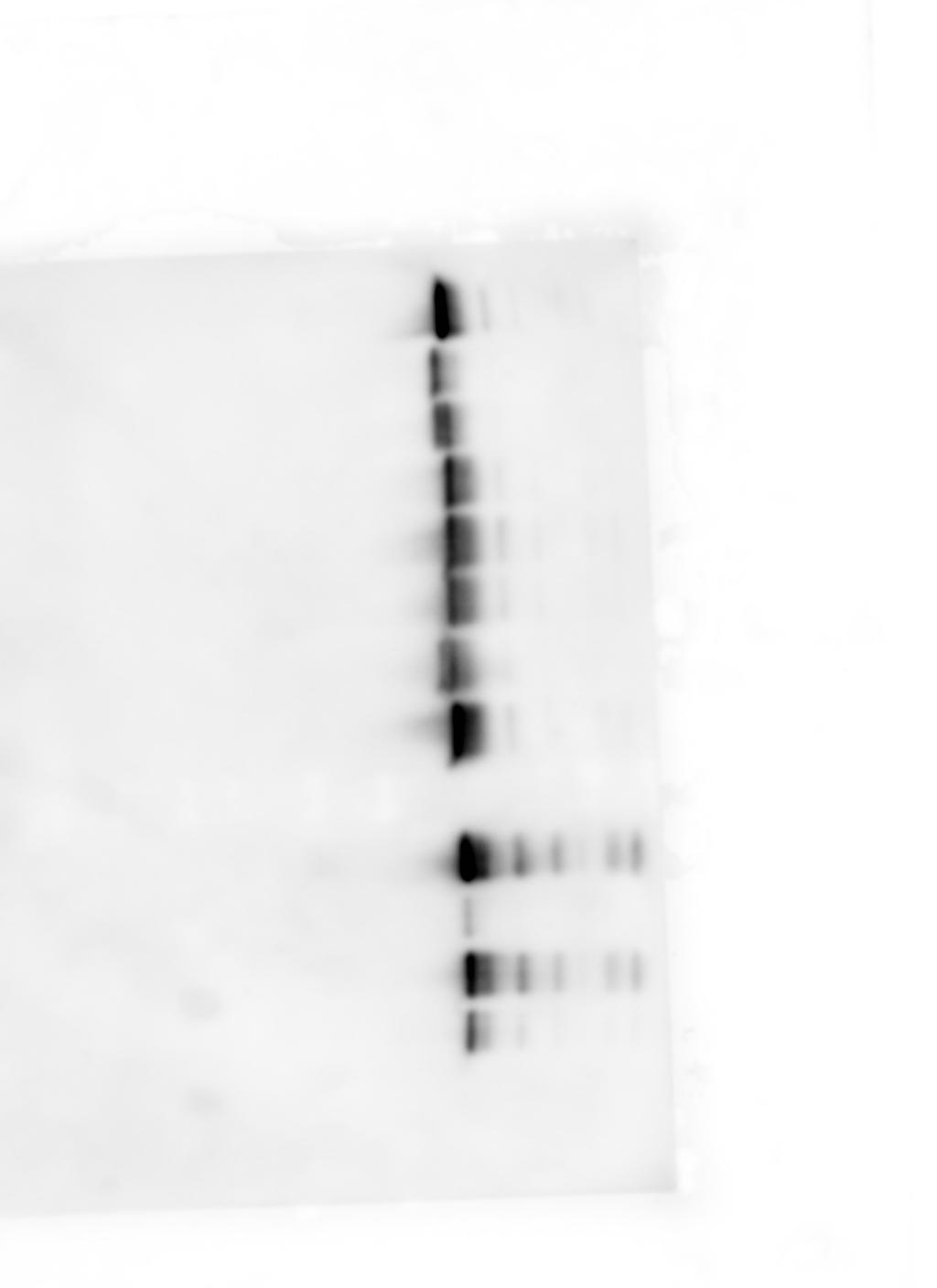

Supplement: Figure 3—source data 4. [file elife-34878-fig3-data4.zip › Figure 3 ΓÇô Source Data 4.jpg]

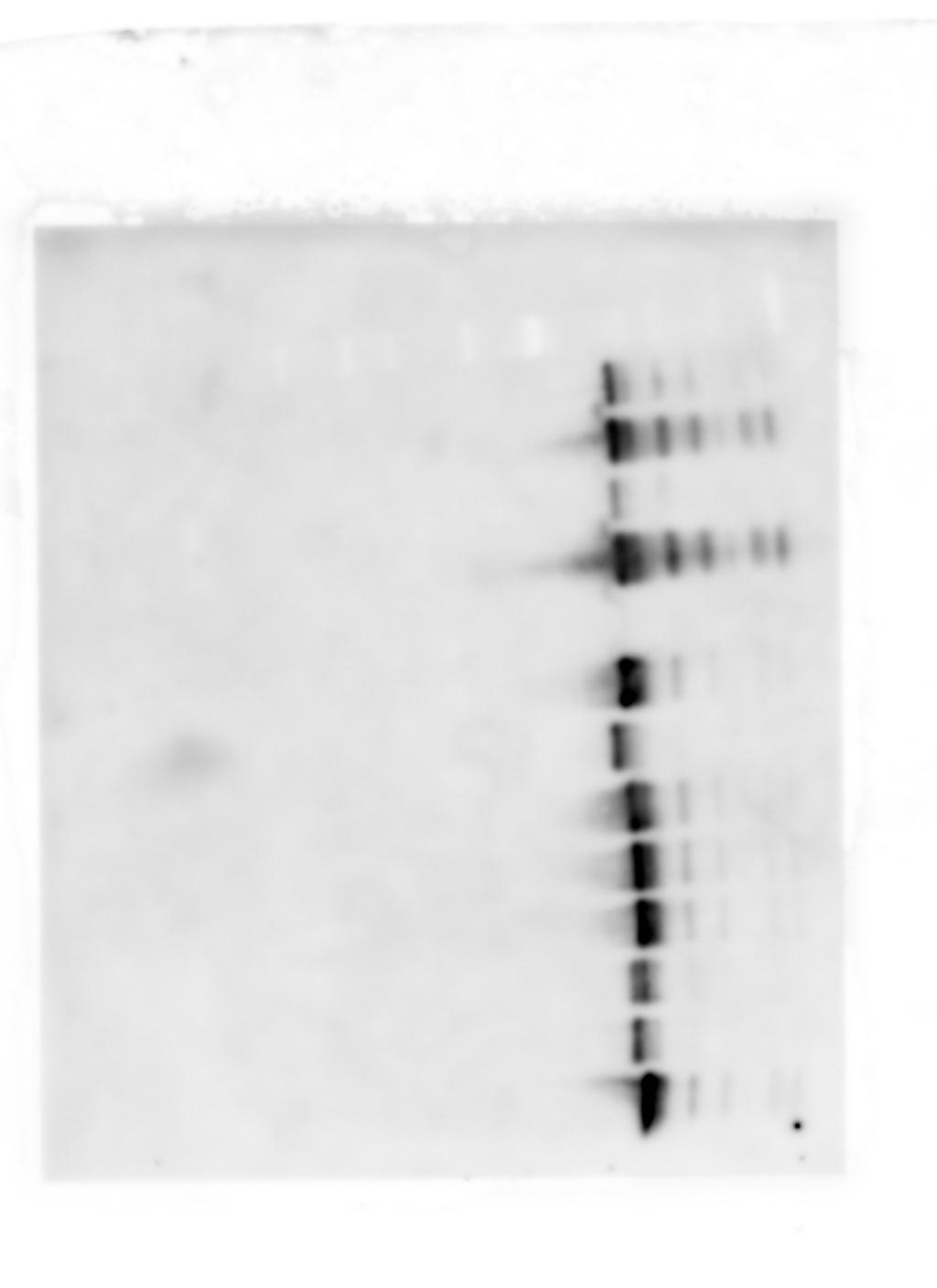

Supplement: Figure 3—source data 5. [file elife-34878-fig3-data5.zip › Figure 3 ΓÇô Source Data 5.jpg]
