# Supplementary material for: Organisms with alternative genetic codes resolve unassigned codons via mistranslation and ribosomal rescue
Source: eLife. 2018 Oct 30;7:e34878. doi: 10.7554/eLife.34878 (PMC6207430; doi:10.7554/eLife.34878)
Supplement: Supplementary file 1. [file elife-34878-supp1.docx]

**Organisms with alternative genetic codes resolve unassigned codons via mistranslation and ribosomal rescue**

**Authors:** Natalie J. Ma, Colin F. Hemez, Karl W. Barber, Jesse Rinehart, Farren J. Isaacs

**Supplementary Materials**

**Nucleotide sequence of UAG-ending GFP construct**

GFP Protein in **Green**

6xHis-tag in **Blue**

UAG stop codon in **Orange**

Nucleotides after stop codon in **Red**

Transcriptional terminator sequence **underlined**

**ATGAGTAAAGGAGAAGAACTTTTCACTGGAGTTGTCCCAATTCTTGTTGAATTAGATGGTGATGTTAATGGGCACAAATTTTCTGTCAGTGGAGAGGGTGAAGGTGATGCAACATACGGAAAACTTACCCTTAAATTTATTTGCACTACTGGAAAACTACCTGTTCCATGGCCAACACTTGTCACTACTTTCTCTTATGGTGTTCAATGCTTTTCCCGTTATCCGGATCACATGAAACGGCATGACTTTTTCAAGAGTGCCATGCCCGAAGGTTATGTACAGGAACGCACTATATCTTTCAAAGATGACGGGAACTACAAGACGCGTGCTGAAGTCAAGTTTGAAGGTGATACCCTTGTTAATCGTATCGAGTTAAAAGGTATTGATTTTAAAGAAGATGGAAACATTCTCGGACACAAACTCGAATACAACTATAACTCACACAATGTATACATCACGGCAGACAAACAAAAGAATGGAATCAAAGCTAACTTCAAAATTCGCCACAACATTGAAGATGGATCCGTTCAACTAGCAGACCATTATCAACAAAATACTCCAATTGGCGATGGCCCTGTCCTTTTACCAGACAACCATTACCTGTCGACACAATCTGCCCTTTCGAAAGATCCCAACGAAAAGCGTGACCACATGGTCCTTCTTGAGTTTGTAACTGCTGCTGGGATTACACATGGCATGGATGAGCTCTACAAACTCGAGCACCACCACCACCACCACTAGGGCGCTCGGGGATCCCATGGTACGCGTGCTAGAGGCATCAAATAAAACGAAAGGCTCAGTCGAAAGACTGGGCCTTTCGTTTTATCTGTTGTTTGTCGGTGA**

**Table S1 – C-terminal fragments detected by Mass Spectrometry for GFP constructs ending in TAG and TAA expressed in +RF1 and ΔRF1 backgrounds**

| **Strain** | **GFP** | **Reading Frame** | **Sequence** | **Length** | **Modifications** | **Missed cleavages** | **Raw file** | **Fraction** | **Experiment** | **MS/MS m/z** | **Charge** | **m/z** | **Mass** | **MS/MS Scan Number** | **Score** |
| --- | --- | --- | --- | --- | --- | --- | --- | --- | --- | --- | --- | --- | --- | --- | --- |
| GRO.DD | UAG-GFP | 2 | LEHHHHHHALGD | 12 | Unmodified | 0 | VOT16-1542 | 1 | 1170 | 360.67 | 4 | 360.6723 | 1438.66 | 1751 | 60.691 |
| GRO.DD | UAG-GFP | 3 | LEHHHHHHASGIP | 13 | Unmodified | 0 | VOT16-1542 | 1 | 1170 | 503.58 | 3 | 503.58 | 1507.718 | 1846 | 15.941 |
| GRO.DD | UAG-GFP | 2 | LEHHHHHHGDAANDENYALDD | 21 | Unmodified | 0 | VOT16-1542 | 1 | 1170 | 612.51 | 4 | 612.5091 | 2446.007 | 2210 | 63.302 |
| GRO.DD | UAG-GFP | 2 | LEHHHHHHGDPMVR | 14 | Unmodified | 0 | VOT16-1542 | 1 | 1170 | 580.28 | 3 | 580.2746 | 1737.802 | 1785 | 159.12 |
| GRO.DD | UAG-GFP | 3 | LEHHHHHHGSGIPWYAC | 17 | Unmodified | 0 | VOT16-1542 | 1 | 1170 | 519.74 | 4 | 519.4855 | 2073.913 | 3771 | 15.273 |
| GRO.DD | UAG-GFP | 3 | LEHHHHHHLSGI | 12 | Unmodified | 0 | VOT16-1542 | 1 | 1170 | 485.24 | 3 | 485.2447 | 1452.712 | 1726 | 14.009 |
| GRO.DD | UAG-GFP | 3 | LEHHHHHHSGAANDENYALDD | 21 | Unmodified | 0 | VOT16-1542 | 1 | 1170 | 605.76 | 4 | 605.5103 | 2418.012 | 2156 | 73.138 |
| GRO.DD | UAG-GFP | 3 | LEHHHHHHSGIPAANDENYALDD | 23 | Unmodified | 0 | VOT16-1542 | 1 | 1170 | 658.54 | 4 | 658.0445 | 2628.149 | 3264 | 9.5658 |
| GRO.DD | UAG-GFP | 3 | LEHHHHHHSGIPWYAC | 16 | Unmodified | 0 | VOT16-1542 | 1 | 1170 | 673.64 | 3 | 673.3044 | 2016.891 | 3801 | 82.202 |
| GRO.DD | UAG-GFP | 2 | ALGDPMVR | 8 | Unmodified | 0 | VOT16-1542 | 1 | 1170 | 429.73 | 2 | 429.7287 | 857.4429 | 4130 | 98.458 |
| GRO.DD | UAG-GFP | 2 | ALGDPMVR | 8 | Oxidation (M) | 0 | VOT16-1542 | 1 | 1170 | 437.73 | 2 | 437.7262 | 873.4378 | 2808 | 73.665 |
| GRO.DD | UAG-GFP | 1 | GSHGAANDENYALDD | 15 | Unmodified | 0 | VOT16-1542 | 1 | 1170 | 774.82 | 2 | 774.8135 | 1547.612 | 3494 | 48.899 |
| GRO.DD | UAG-GFP | 1 | HAANDENYALDD | 12 | Unmodified | 0 | VOT16-1542 | 1 | 1170 | 674.28 | 2 | 674.276 | 1346.537 | 3373 | 102.05 |
| GRO.DD | UAG-GFP | N/A | LEHHHHHHAAAANDENYALDD | 21 | Unmodified | 0 | VOT16-1542 | 1 | 1170 | 806.35 | 3 | 806.3516 | 2416.033 | 2213 | 63.302 |
| GRO.DD | UAG-GFP | N/A | LEHHHHHHAAANDENYALDD | 20 | Unmodified | 0 | VOT16-1542 | 1 | 1170 | 587.51 | 4 | 587.2562 | 2344.996 | 2168 | 62.203 |
| GRO.DD | UAG-GFP | 1 | LEHHHHHHAANDENYALDD | 19 | Unmodified | 0 | VOT16-1542 | 1 | 1170 | 1138.49 | 2 | 1137.987 | 2273.959 | 2118 | 112.82 |
| GRO.DD | UAG-GFP | 1 | LEHHHHHHAANDENYALDD | 19 | Unmodified | 0 | VOT16-1542 | 1 | 1170 | 759.33 | 3 | 758.9935 | 2273.959 | 2371 | 68.576 |
| GRO.DD | UAG-GFP | 1 | LEHHHHHHAANDENYALDD | 19 | Unmodified | 0 | VOT16-1542 | 1 | 1170 | 569.75 | 4 | 569.497 | 2273.959 | 2155 | 58.98 |
| GRO.DD | UAG-GFP | 1 | LEHHHHHHAANDENYALDD | 19 | Unmodified | 0 | VOT16-1542 | 1 | 1170 | 569.75 | 4 | 569.497 | 2273.959 | 2578 | 38.777 |
| GRO.DD | UAG-GFP | 2 | LEHHHHHHALGDPMVR | 16 | Unmodified | 0 | VOT16-1542 | 1 | 1170 | 481.74 | 4 | 481.488 | 1921.923 | 2228 | 95.822 |
| GRO.DD | UAG-GFP | 2 | LEHHHHHHALGDPMVR | 16 | Unmodified | 0 | VOT16-1542 | 1 | 1170 | 641.65 | 3 | 641.6483 | 1921.923 | 2173 | 55.011 |
| GRO.DD | UAG-GFP | 2 | LEHHHHHHGDPAANDENYALDD | 22 | Unmodified | 0 | VOT16-1542 | 1 | 1170 | 637.02 | 4 | 636.7722 | 2543.06 | 2372 | 43.696 |
| GRO.DD | UAG-GFP | 2 | LEHHHHHHGDPM | 12 | Unmodified | 0 | VOT16-1542 | 1 | 1170 | 495.22 | 3 | 495.2181 | 1482.632 | 1775 | 33.1 |
| GRO.DD | UAG-GFP | 2 | LEHHHHHHGDPMV | 13 | Unmodified | 0 | VOT16-1542 | 1 | 1170 | 528.24 | 3 | 528.2409 | 1581.701 | 1911 | 91.313 |
| GRO.DD | UAG-GFP | 2 | LEHHHHHHGDPMVR | 14 | Unmodified | 0 | VOT16-1542 | 1 | 1170 | 435.46 | 4 | 435.4577 | 1737.802 | 1804 | 70.628 |
| GRO.DD | UAG-GFP | 2 | LEHHHHHHGDPMVR | 14 | Unmodified | 0 | VOT16-1542 | 1 | 1170 | 348.77 | 5 | 348.5677 | 1737.802 | 1826 | 50.222 |
| GRO.DD | UAG-GFP | 2 | LEHHHHHHGGDPMVR | 15 | Unmodified | 0 | VOT16-1542 | 1 | 1170 | 449.71 | 4 | 449.7131 | 1794.823 | 1787 | 13.791 |
| GRO.DD | UAG-GFP | 3 | LEHHHHHHGR | 10 | Unmodified | 0 | VOT16-1542 | 1 | 1170 | 324.91 | 4 | 324.9106 | 1295.613 | 2794 | 12.576 |
| GRO.DD | UAG-GFP | 3 | LEHHHHHHKSGIPWYAC | 17 | Unmodified | 1 | VOT16-1542 | 1 | 1170 | 537.51 | 4 | 537.2539 | 2144.986 | 3334 | 11.318 |
| GRO.DD | UAG-GFP | 2 | LEHHHHHHLGDPMVR | 15 | Unmodified | 0 | VOT16-1542 | 1 | 1170 | 463.73 | 4 | 463.7288 | 1850.886 | 2061 | 77.42 |
| GRO.DD | UAG-GFP | 2 | LEHHHHHHQALGDPMVR | 17 | Unmodified | 0 | VOT16-1542 | 1 | 1170 | 513.75 | 4 | 513.5027 | 2049.982 | 2138 | 78.95 |
| GRO.DD | UAG-GFP | 1 | LEHHHHHHQGAR | 12 | Unmodified | 0 | VOT16-1542 | 1 | 1170 | 374.68 | 4 | 374.6845 | 1494.709 | 1496 | 30.889 |
| GRO.DD | UAG-GFP | 3 | LEHHHHHHQR | 10 | Unmodified | 0 | VOT16-1542 | 1 | 1170 | 342.92 | 4 | 342.6699 | 1366.65 | 1622 | 17.473 |
| GRO.DD | UAG-GFP | 3 | LEHHHHHHQR | 10 | Unmodified | 0 | VOT16-1542 | 1 | 1170 | 343.22 | 4 | 342.6699 | 1366.65 | 2832 | 9.6551 |
| GRO.DD | UAG-GFP | 3 | LEHHHHHHSGIP | 12 | Unmodified | 0 | VOT16-1542 | 1 | 1170 | 479.9 | 3 | 479.9009 | 1436.681 | 1856 | 63.694 |
| GRO.DD | UAG-GFP | 3 | LEHHHHHHSGIPW | 13 | Unmodified | 0 | VOT16-1542 | 1 | 1170 | 541.93 | 3 | 541.9274 | 1622.76 | 3171 | 23.468 |
| GRO.DD | UAG-GFP | 3 | LEHHHHHHSGIPWYA | 15 | Unmodified | 0 | VOT16-1542 | 1 | 1170 | 465.47 | 4 | 465.2225 | 1856.861 | 3743 | 43.03 |
| GRO.DD | UAG-GFP | 3 | LEHHHHHHSGIPWYA | 15 | Unmodified | 0 | VOT16-1542 | 1 | 1170 | 620.3 | 3 | 619.9609 | 1856.861 | 3694 | 36.292 |
| GRO.DD | UAG-GFP | 3 | LEHHHHHHSGIPWYAC | 16 | Unmodified | 0 | VOT16-1542 | 1 | 1170 | 505.48 | 4 | 505.2301 | 2016.891 | 3888 | 48.527 |
| GRO.DD | UAG-GFP | 3 | LEHHHHHHSGIPWYAC | 16 | Unmodified | 0 | VOT16-1542 | 1 | 1170 | 505.48 | 4 | 505.2301 | 2016.891 | 4226 | 27.767 |
| GRO.DD | UAG-GFP | 1 | LEHHHHHHVA | 10 | Unmodified | 0 | VOT16-1542 | 1 | 1170 | 418.54 | 3 | 418.5394 | 1252.596 | 1684 | 26.442 |
| GRO.DD | UAG-GFP | 1 | LEHHHHHHYGAR | 12 | Unmodified | 0 | VOT16-1542 | 1 | 1170 | 383.44 | 4 | 383.4357 | 1529.714 | 1766 | 49.189 |
| GRO.DD | UAG-GFP | 1 | LEHHHHHHYGAR | 12 | Unmodified | 0 | VOT16-1542 | 1 | 1170 | 306.95 | 5 | 306.95 | 1529.714 | 1659 | 19.099 |
| GRO.DD | UAG-GFP | 3 | LGDPMVR | 7 | Unmodified | 0 | VOT16-1542 | 1 | 1170 | 394.21 | 2 | 394.2102 | 786.4058 | 4071 | 70.912 |
| GRO.DD | UAG-GFP | 1 | LGLAANDENYALDD | 14 | Unmodified | 0 | VOT16-1542 | 1 | 1170 | 747.34 | 2 | 747.3414 | 1492.668 | 7357 | 36.944 |
| GRO.DD | UAG-GFP | 1 | LGLSAANDENYALDD | 15 | Unmodified | 0 | VOT16-1542 | 1 | 1170 | 790.86 | 2 | 790.8574 | 1579.7 | 7324 | 38.995 |
| GRO.DD | UAG-GFP | 2 | SGAANDENYALDD | 13 | Unmodified | 0 | VOT16-1542 | 1 | 1170 | 677.77 | 2 | 677.7733 | 1353.532 | 4252 | 32.933 |
| GRO.DD | UAG-GFP | 2 | SHGAANDENYALDD | 14 | Unmodified | 0 | VOT16-1542 | 1 | 1170 | 746.8 | 2 | 746.3028 | 1490.591 | 3401 | 79.875 |
| GRO.DD | UAG-GFP | 3 | VLEASNK | 7 | Unmodified | 0 | VOT16-1542 | 1 | 1170 | 380.71 | 2 | 380.7136 | 759.4127 | 1987 | 143.02 |
| GRO.DD | UAG-GFP | 3 | VLEASNK | 7 | Unmodified | 0 | VOT16-1542 | 1 | 1170 | 380.71 | 2 | 380.7136 | 759.4127 | 2172 | 77.741 |
| GRO.DD.prfA+ | UAG-GFP | 1 | LEHHHHHHAANDENYALDD | 19 | Unmodified | 0 | VOT16-1545 | 1 | 1171 | 759 | 3 | 758.9935 | 2273.959 | 2215 | 83.54 |
| GRO.DD.prfA+ | UAG-GFP | 1 | LEHHHHHHAANDENYALDD | 19 | Unmodified | 0 | VOT16-1545 | 1 | 1171 | 569.75 | 4 | 569.497 | 2273.959 | 2262 | 58.98 |
| GRO.DD.prfA+ | UAG-GFP | 1 | LEHHHHHHAANDENYALDD | 19 | Unmodified | 0 | VOT16-1545 | 1 | 1171 | 759.33 | 3 | 758.9935 | 2273.959 | 1989 | 12.193 |
| GRO.DD.prfA+ | UAA-GFP | 1 | LEHHHHHHAANDENYALDD | 19 | Unmodified | 0 | VOT16-1551 | 1 | 1181 | 569.75 | 4 | 569.497 | 2273.959 | 2041 | 88.385 |
| GRO.DD.prfA+ | UAA-GFP | 1 | LEHHHHHHAANDENYALDD | 19 | Unmodified | 0 | VOT16-1551 | 1 | 1181 | 759.33 | 3 | 758.9935 | 2273.959 | 2025 | 39.338 |
| GRO.DD | UAG-GFP | 1 | HAANDENYALDD | 12 | Unmodified | 0 | VOT16-1571 | 1 | 1170_R | 674.28 | 2 | 674.276 | 1346.537 | 3171 | 120.32 |
| GRO.DD | UAG-GFP | N/A | LEHHHHHHAAAANDENYALDD | 21 | Unmodified | 0 | VOT16-1571 | 1 | 1170_R | 806.69 | 3 | 806.3516 | 2416.033 | 1968 | 65.423 |
| GRO.DD | UAG-GFP | 1 | LEHHHHHHAANDENYALDD | 19 | Unmodified | 0 | VOT16-1571 | 1 | 1170_R | 1138.48 | 2 | 1137.987 | 2273.959 | 1843 | 146.78 |
| GRO.DD | UAG-GFP | 2 | LEHHHHHHALGDPMVR | 16 | Unmodified | 0 | VOT16-1571 | 1 | 1170_R | 641.98 | 3 | 641.6483 | 1921.923 | 1808 | 121.82 |
| GRO.DD | UAG-GFP | 1 | LEHHHHHHGAANDENYALDD | 20 | Unmodified | 0 | VOT16-1571 | 1 | 1170_R | 582.97 | 4 | 583.7523 | 2330.98 | 1832 | 31.41 |
| GRO.DD | UAG-GFP | 2 | LEHHHHHHGALGDPMVR | 17 | Unmodified | 0 | VOT16-1571 | 1 | 1170_R | 494.97 | 4 | 495.7434 | 1978.945 | 1888 | 18.58 |
| GRO.DD | UAG-GFP | 3 | LEHHHHHHGR | 10 | Unmodified | 0 | VOT16-1571 | 1 | 1170_R | 324.91 | 4 | 324.9106 | 1295.613 | 1044 | 38.754 |
| GRO.DD | UAG-GFP | 1 | LEHHHHHHHAANDENYALDD | 20 | Unmodified | 0 | VOT16-1571 | 1 | 1170_R | 805.01 | 3 | 804.6798 | 2411.018 | 1786 | 99.044 |
| GRO.DD | UAG-GFP | 1 | LEHHHHHHHHAANDENYALDD | 21 | Unmodified | 0 | VOT16-1571 | 1 | 1170_R | 638.53 | 4 | 638.0264 | 2548.077 | 1770 | 72.359 |
| GRO.DD | UAG-GFP | 2 | LEHHHHHHLGDPMVR | 15 | Unmodified | 0 | VOT16-1571 | 1 | 1170_R | 618.3 | 3 | 617.9693 | 1850.886 | 1777 | 97.767 |
| GRO.DD | UAG-GFP | 3 | LEHHHHHHLGR | 11 | Unmodified | 0 | VOT16-1571 | 1 | 1170_R | 353.18 | 4 | 353.1816 | 1408.697 | 1286 | 47.302 |
| GRO.DD | UAG-GFP | N/A | LEHHHHHHNRG | 11 | Unmodified | 1 | VOT16-1571 | 1 | 1170_R | 470.89 | 3 | 470.8927 | 1409.656 | 1608 | 33.87 |
| GRO.DD | UAG-GFP | 2 | LEHHHHHHQALGDPMVR | 17 | Unmodified | 0 | VOT16-1571 | 1 | 1170_R | 513.5 | 4 | 513.5027 | 2049.982 | 1857 | 79.837 |
| GRO.DD | UAG-GFP | 1 | LEHHHHHHQGAAANDENYALDD | 22 | Unmodified | 0 | VOT16-1571 | 1 | 1170_R | 633.78 | 4 | 633.5262 | 2530.076 | 1955 | 32.797 |
| GRO.DD | UAG-GFP | 1 | LEHHHHHHQGAR | 12 | Unmodified | 0 | VOT16-1571 | 1 | 1170_R | 374.68 | 4 | 374.6845 | 1494.709 | 1057 | 70.942 |
| GRO.DD | UAG-GFP | 3 | LEHHHHHHQR | 10 | Unmodified | 0 | VOT16-1571 | 1 | 1170_R | 342.67 | 4 | 342.6699 | 1366.65 | 1049 | 37.344 |
| GRO.DD | UAG-GFP | 1 | LEHHHHHHVAR | 11 | Unmodified | 0 | VOT16-1571 | 1 | 1170_R | 470.91 | 3 | 470.5731 | 1408.697 | 1309 | 41.399 |
| GRO.DD | UAG-GFP | 1 | LEHHHHHHYGAR | 12 | Unmodified | 0 | VOT16-1571 | 1 | 1170_R | 510.91 | 3 | 510.9118 | 1529.714 | 1161 | 141.1 |
| GRO.DD | UAG-GFP | 2 | ALGAANDENYALDD | 14 | Unmodified | 0 | VOT16-1571 | 1 | 1170_R | 726.32 | 2 | 726.3179 | 1450.621 | 5459 | 27.005 |
| GRO.DD | UAG-GFP | 2 | ALGDAANDENYALDD | 15 | Unmodified | 0 | VOT16-1571 | 1 | 1170_R | 783.83 | 2 | 783.8314 | 1565.648 | 5712 | 41.621 |
| GRO.DD | UAG-GFP | 2 | ALGDPMVR | 8 | Unmodified | 0 | VOT16-1571 | 1 | 1170_R | 429.73 | 2 | 429.7287 | 857.4429 | 3838 | 93.374 |
| GRO.DD | UAG-GFP | 2 | ALGDPMVR | 8 | Oxidation (M) | 0 | VOT16-1571 | 1 | 1170_R | 437.73 | 2 | 437.7262 | 873.4378 | 2513 | 83.313 |
| GRO.DD | UAG-GFP | 2 | GSVEAANDENYALDD | 15 | Unmodified | 0 | VOT16-1571 | 1 | 1170_R | 791.83 | 2 | 791.8288 | 1581.643 | 4775 | 22.855 |
| GRO.DD | UAG-GFP | 1 | HAANDENYALDD | 12 | Deamidation (NQ) | 0 | VOT16-1571 | 1 | 1170_R | 674.77 | 2 | 674.768 | 1347.522 | 3309 | 54.343 |
| GRO.DD | UAG-GFP | N/A | LEHHHHHHAAAANDENYALDD | 21 | Unmodified | 0 | VOT16-1571 | 1 | 1170_R | 605.27 | 4 | 605.0155 | 2416.033 | 2016 | 36.127 |
| GRO.DD | UAG-GFP | 1 | LEHHHHHHAANDENYALDD | 19 | Unmodified | 0 | VOT16-1571 | 1 | 1170_R | 759.33 | 3 | 758.9935 | 2273.959 | 2090 | 51.495 |
| GRO.DD | UAG-GFP | 1 | LEHHHHHHAANDENYALDD | 19 | Unmodified | 0 | VOT16-1571 | 1 | 1170_R | 569.5 | 4 | 569.497 | 2273.959 | 2158 | 46.249 |
| GRO.DD | UAG-GFP | 1 | LEHHHHHHAANDENYALDD | 19 | Unmodified | 0 | VOT16-1571 | 1 | 1170_R | 569.75 | 4 | 569.497 | 2273.959 | 2447 | 32.366 |
| GRO.DD | UAG-GFP | 1 | LEHHHHHHAANDENYALDD | 19 | Unmodified | 0 | VOT16-1571 | 1 | 1170_R | 570 | 4 | 569.497 | 2273.959 | 2322 | 13.636 |
| GRO.DD | UAG-GFP | 2 | LEHHHHHHALGD | 12 | Unmodified | 0 | VOT16-1571 | 1 | 1170_R | 480.56 | 3 | 480.5607 | 1438.66 | 1432 | 38.875 |
| GRO.DD | UAG-GFP | 2 | LEHHHHHHALGD | 12 | Unmodified | 0 | VOT16-1571 | 1 | 1170_R | 360.67 | 4 | 360.6723 | 1438.66 | 1444 | 38.875 |
| GRO.DD | UAG-GFP | 2 | LEHHHHHHALGDPAANDENYALDD | 24 | Unmodified | 0 | VOT16-1571 | 1 | 1170_R | 683.05 | 4 | 682.8025 | 2727.181 | 3375 | 5.9604 |
| GRO.DD | UAG-GFP | 2 | LEHHHHHHALGDPMVR | 16 | Unmodified | 0 | VOT16-1571 | 1 | 1170_R | 481.49 | 4 | 481.488 | 1921.923 | 2125 | 93.237 |
| GRO.DD | UAG-GFP | 2 | LEHHHHHHALGDPMVR | 16 | Unmodified | 0 | VOT16-1571 | 1 | 1170_R | 481.74 | 4 | 481.488 | 1921.923 | 1974 | 71.545 |
| GRO.DD | UAG-GFP | 2 | LEHHHHHHALGDPMVR | 16 | Oxidation (M) | 0 | VOT16-1571 | 1 | 1170_R | 647.31 | 3 | 646.9799 | 1937.918 | 1670 | 66.674 |
| GRO.DD | UAG-GFP | 2 | LEHHHHHHALGDPMVR | 16 | Unmodified | 0 | VOT16-1571 | 1 | 1170_R | 961.97 | 2 | 961.9688 | 1921.923 | 1828 | 55.755 |
| GRO.DD | UAG-GFP | 2 | LEHHHHHHALGDPMVR | 16 | Unmodified | 0 | VOT16-1571 | 1 | 1170_R | 641.65 | 3 | 641.6483 | 1921.923 | 2001 | 15.514 |
| GRO.DD | UAG-GFP | 2 | LEHHHHHHALGDPMVR | 16 | Unmodified | 0 | VOT16-1571 | 1 | 1170_R | 481.99 | 4 | 481.488 | 1921.923 | 2696 | 15.193 |
| GRO.DD | UAG-GFP | 2 | LEHHHHHHGDPM | 12 | Unmodified | 0 | VOT16-1571 | 1 | 1170_R | 495.22 | 3 | 495.2181 | 1482.632 | 1476 | 68.132 |
| GRO.DD | UAG-GFP | 2 | LEHHHHHHGDPM | 12 | Unmodified | 0 | VOT16-1571 | 1 | 1170_R | 371.67 | 4 | 371.6654 | 1482.632 | 1477 | 30.205 |
| GRO.DD | UAG-GFP | 2 | LEHHHHHHGDPMVR | 14 | Unmodified | 0 | VOT16-1571 | 1 | 1170_R | 580.28 | 3 | 580.2746 | 1737.802 | 1515 | 105.19 |
| GRO.DD | UAG-GFP | 2 | LEHHHHHHGDPMVR | 14 | Unmodified | 0 | VOT16-1571 | 1 | 1170_R | 435.46 | 4 | 435.4577 | 1737.802 | 1569 | 90.759 |
| GRO.DD | UAG-GFP | 2 | LEHHHHHHGDPMVR | 14 | Unmodified | 0 | VOT16-1571 | 1 | 1170_R | 435.88 | 4 | 435.4577 | 1737.802 | 2070 | 12.792 |
| GRO.DD | UAG-GFP | 2 | LEHHHHHHGGDPMVR | 15 | Unmodified | 0 | VOT16-1571 | 1 | 1170_R | 449.96 | 4 | 449.7131 | 1794.823 | 1517 | 17.252 |
| GRO.DD | UAG-GFP | 3 | LEHHHHHHGR | 10 | Unmodified | 0 | VOT16-1571 | 1 | 1170_R | 325.7 | 4 | 324.9106 | 1295.613 | 2575 | 14.461 |
| GRO.DD | UAG-GFP | N/A | LEHHHHHHNHAANDENYALDD | 21 | Unmodified | 0 | VOT16-1571 | 1 | 1170_R | 632.77 | 4 | 632.2724 | 2525.061 | 1787 | 7.5824 |
| GRO.DD | UAG-GFP | 1 | LEHHHHHHQGAR | 12 | Unmodified | 0 | VOT16-1571 | 1 | 1170_R | 374.94 | 4 | 374.6845 | 1494.709 | 2831 | 13.589 |
| GRO.DD | UAG-GFP | 1 | LEHHHHHHQGAR | 12 | Unmodified | 0 | VOT16-1571 | 1 | 1170_R | 499.58 | 3 | 499.2436 | 1494.709 | 1060 | 11.919 |
| GRO.DD | UAG-GFP | 3 | LEHHHHHHQL | 10 | Unmodified | 0 | VOT16-1571 | 1 | 1170_R | 442.22 | 3 | 442.2184 | 1323.633 | 8953 | 20.777 |
| GRO.DD | UAG-GFP | 3 | LEHHHHHHQLAANDENYALDD | 21 | Unmodified | 0 | VOT16-1571 | 1 | 1170_R | 630.28 | 4 | 629.7826 | 2515.101 | 2850 | 19.057 |
| GRO.DD | UAG-GFP | 3 | LEHHHHHHQR | 10 | Unmodified | 0 | VOT16-1571 | 1 | 1170_R | 342.67 | 4 | 342.6699 | 1366.65 | 1425 | 15.144 |
| GRO.DD | UAG-GFP | 3 | LEHHHHHHQR | 10 | Unmodified | 0 | VOT16-1571 | 1 | 1170_R | 342.67 | 4 | 342.6699 | 1366.65 | 1281 | 9.6551 |
| GRO.DD | UAG-GFP | 1 | LEHHHHHHRGA | 11 | Unmodified | 1 | VOT16-1571 | 1 | 1170_R | 456.56 | 3 | 456.5574 | 1366.65 | 1050 | 13.809 |
| GRO.DD | UAG-GFP | 3 | LEHHHHHHSGI | 11 | Unmodified | 0 | VOT16-1571 | 1 | 1170_R | 447.55 | 3 | 447.55 | 1339.628 | 1299 | 67.207 |
| GRO.DD | UAG-GFP | 3 | LEHHHHHHSGIP | 12 | Unmodified | 0 | VOT16-1571 | 1 | 1170_R | 479.9 | 3 | 479.9009 | 1436.681 | 1588 | 53.567 |
| GRO.DD | UAG-GFP | 3 | LEHHHHHHSGIP | 12 | Unmodified | 0 | VOT16-1571 | 1 | 1170_R | 360.18 | 4 | 360.1775 | 1436.681 | 1597 | 32.254 |
| GRO.DD | UAG-GFP | 3 | LEHHHHHHSGIPW | 13 | Unmodified | 0 | VOT16-1571 | 1 | 1170_R | 541.93 | 3 | 541.9274 | 1622.76 | 2988 | 34.96 |
| GRO.DD | UAG-GFP | 3 | LEHHHHHHSGIPWYA | 15 | Unmodified | 0 | VOT16-1571 | 1 | 1170_R | 619.96 | 3 | 619.9609 | 1856.861 | 3996 | 60.157 |
| GRO.DD | UAG-GFP | 3 | LEHHHHHHSGIPWYA | 15 | Unmodified | 0 | VOT16-1571 | 1 | 1170_R | 619.96 | 3 | 619.9609 | 1856.861 | 3847 | 51.726 |
| GRO.DD | UAG-GFP | 3 | LEHHHHHHSGIPWYAC | 16 | Unmodified | 0 | VOT16-1571 | 1 | 1170_R | 673.31 | 3 | 673.3044 | 2016.891 | 4492 | 72.891 |
| GRO.DD | UAG-GFP | 3 | LEHHHHHHSGIPWYAC | 16 | Unmodified | 0 | VOT16-1571 | 1 | 1170_R | 505.48 | 4 | 505.2301 | 2016.891 | 4491 | 64.104 |
| GRO.DD | UAG-GFP | 3 | LEHHHHHHSGIPWYAC | 16 | Unmodified | 0 | VOT16-1571 | 1 | 1170_R | 505.48 | 4 | 505.2301 | 2016.891 | 4965 | 41.621 |
| GRO.DD | UAG-GFP | 3 | LEHHHHHHSGIPWYAC | 16 | Unmodified | 0 | VOT16-1571 | 1 | 1170_R | 673.3 | 3 | 673.3044 | 2016.891 | 4194 | 40.278 |
| GRO.DD | UAG-GFP | 3 | LEHHHHHHSGIPWYAC | 16 | Unmodified | 0 | VOT16-1571 | 1 | 1170_R | 505.73 | 4 | 505.2301 | 2016.891 | 5142 | 34.432 |
| GRO.DD | UAG-GFP | 3 | LEHHHHHHSGIPWYAC | 16 | Unmodified | 0 | VOT16-1571 | 1 | 1170_R | 505.23 | 4 | 505.2301 | 2016.891 | 4198 | 33.842 |
| GRO.DD | UAG-GFP | 3 | LEHHHHHHSLGAANDENYALDD | 22 | Unmodified | 0 | VOT16-1571 | 1 | 1170_R | 634.03 | 4 | 633.7813 | 2531.096 | 2787 | 7.2559 |
| GRO.DD | UAG-GFP | 3 | LEHHHHHHSLGDPMVR | 16 | Unmodified | 0 | VOT16-1571 | 1 | 1170_R | 485.74 | 4 | 485.4868 | 1937.918 | 2061 | 11.643 |
| GRO.DD | UAG-GFP | 3 | LEHHHHHHWGDPMV | 14 | Unmodified | 0 | VOT16-1571 | 1 | 1170_R | 443.95 | 4 | 442.9523 | 1767.78 | 1455 | 13.746 |
| GRO.DD | UAG-GFP | 1 | LEHHHHHHYGA | 11 | Unmodified | 0 | VOT16-1571 | 1 | 1170_R | 458.88 | 3 | 458.8781 | 1373.613 | 1300 | 17.473 |
| GRO.DD | UAG-GFP | 1 | LEHHHHHHYGAR | 12 | Unmodified | 0 | VOT16-1571 | 1 | 1170_R | 383.44 | 4 | 383.4357 | 1529.714 | 1298 | 73.666 |
| GRO.DD | UAG-GFP | 1 | LEHHHHHHYGAR | 12 | Unmodified | 0 | VOT16-1571 | 1 | 1170_R | 306.95 | 5 | 306.95 | 1529.714 | 1174 | 24.696 |
| GRO.DD | UAG-GFP | 3 | LGDPMVR | 7 | Unmodified | 0 | VOT16-1571 | 1 | 1170_R | 394.21 | 2 | 394.2102 | 786.4058 | 3772 | 97.068 |
| GRO.DD | UAG-GFP | 1 | LGLSAANDENYALDD | 15 | Unmodified | 0 | VOT16-1571 | 1 | 1170_R | 790.86 | 2 | 790.8574 | 1579.7 | 7051 | 38.376 |
| GRO.DD | UAG-GFP | 2 | SGAANDENYALDD | 13 | Unmodified | 0 | VOT16-1571 | 1 | 1170_R | 677.77 | 2 | 677.7733 | 1353.532 | 4016 | 46.069 |
| GRO.DD | UAG-GFP | 2 | SHGAANDENYALDD | 14 | Unmodified | 0 | VOT16-1571 | 1 | 1170_R | 746.3 | 2 | 746.3028 | 1490.591 | 3166 | 126.83 |
| GRO.DD | UAG-GFP | 3 | VLEASNK | 7 | Unmodified | 0 | VOT16-1571 | 1 | 1170_R | 380.71 | 2 | 380.7136 | 759.4127 | 1726 | 143.02 |
| GRO.DD | UAG-GFP | 3 | VLEASNKAANDENYALDD | 18 | Phospho (STY),Deamidation (NQ) | 1 | VOT16-1571 | 1 | 1170_R | 678.28 | 3 | 678.2843 | 2031.831 | 1818 | 49.537 |
| GRO.DD.prfA+_R | UAG-GFP | 1 | LEHHHHHHAANDENYALDD | 19 | Unmodified | 0 | VOT16-1568 | 1 | 1171_R | 569.5 | 4 | 569.497 | 2273.959 | 2157 | 9.0576 |
| GRO.DD.prfA+_R | UAG-GFP | 2 | LEHHHHHHTTTK | 12 | Unmodified | 0 | VOT16-1568 | 1 | 1171_R | 505.92 | 3 | 505.5835 | 1513.729 | 2270 | 7.7558 |
| GRO.DD.prfA+ | UAA-GFP | 1 | LEHHHHHHAANDENYALDD | 19 | Unmodified | 0 | VOT16-1562 | 1 | 1181_R | 758.99 | 3 | 758.9935 | 2273.959 | 2034 | 16.924 |
| GRO.DD.prfA+ | UAA-GFP | 1 | LEHHHHHHAANDENYALDD | 19 | Unmodified | 0 | VOT16-1562 | 1 | 1181_R | 569.75 | 4 | 569.497 | 2273.959 | 2081 | 15.441 |
| GRO.DD | UAG-GFP | N/A | AANDENYALDD | 11 | Unmodified | 0 | VOT16-1571 | 1 | 1170_R | 605.75 | 2 | 605.7466 | 1209.479 | 3845 | 39.625 |
| GRO.DD | UAG-GFP | 1 | GSHGTR | 6 | Unmodified | 0 | VOT16-1542 | 1 | 1170 | 307.65 | 2 | 307.6539 | 613.2932 | 6707 | 71.949 |
| GRO.DD | UAG-GFP | 1 | LEHHHHHH | 8 | Unmodified | 0 | VOT16-1542 | 1 | 1170 | 361.84 | 3 | 361.8375 | 1082.491 | 1149 | 24.632 |
| GRO.DD.prfA+ | UAG-GFP | 1 | LEHHHHHH | 8 | Unmodified | 0 | VOT16-1545 | 1 | 1171 | 361.84 | 3 | 361.8375 | 1082.491 | 537 | 38.972 |
| GRO.DD.prfA+ | UAG-GFP | 1 | LEHHHHHH | 8 | Unmodified | 0 | VOT16-1545 | 1 | 1171 | 361.84 | 3 | 361.8375 | 1082.491 | 1029 | 37.646 |
| GRO.DD.prfA+ | UAG-GFP | 1 | LEHHHHHH | 8 | Unmodified | 0 | VOT16-1545 | 1 | 1171 | 361.84 | 3 | 361.8375 | 1082.491 | 4019 | 23.523 |
| GRO.DD | UAA-GFP | 1 | LEHHHHHH | 8 | Unmodified | 0 | VOT16-1548 | 1 | 1180 | 361.84 | 3 | 361.8375 | 1082.491 | 606 | 23.019 |
| GRO.DD | UAA-GFP | 1 | LEHHHHHH | 8 | Unmodified | 0 | VOT16-1548 | 1 | 1180 | 361.84 | 3 | 361.8375 | 1082.491 | 1252 | 23.019 |
| GRO.DD | UAA-GFP | 1 | LEHHHHHH | 8 | Unmodified | 0 | VOT16-1548 | 1 | 1180 | 362.17 | 3 | 361.8375 | 1082.491 | 3822 | 19.36 |
| GRO.DD.prfA+ | UAA-GFP | 1 | LEHHHHHH | 8 | Unmodified | 0 | VOT16-1551 | 1 | 1181 | 361.84 | 3 | 361.8375 | 1082.491 | 492 | 63.306 |
| GRO.DD.prfA+ | UAA-GFP | 1 | LEHHHHHH | 8 | Unmodified | 0 | VOT16-1551 | 1 | 1181 | 361.84 | 3 | 361.8375 | 1082.491 | 1853 | 28.423 |
| GRO.DD.prfA+ | UAA-GFP | 1 | LEHHHHHH | 8 | Unmodified | 0 | VOT16-1551 | 1 | 1181 | 362.17 | 3 | 361.8375 | 1082.491 | 3245 | 36.975 |
| GRO.DD.prfA+ | UAA-GFP | 1 | LEHHHHHH | 8 | Unmodified | 0 | VOT16-1551 | 1 | 1181 | 361.84 | 3 | 361.8375 | 1082.491 | 3372 | 38.806 |
| GRO.DD.prfA+ | UAA-GFP | 1 | LEHHHHHH | 8 | Unmodified | 0 | VOT16-1551 | 1 | 1181 | 361.84 | 3 | 361.8375 | 1082.491 | 3558 | 45.394 |
| GRO.DD.prfA+ | UAA-GFP | 1 | LEHHHHHH | 8 | Unmodified | 0 | VOT16-1551 | 1 | 1181 | 361.49 | 3 | 361.8375 | 1082.491 | 1626 | 24.632 |
| GRO.DD.prfA+ | UAA-GFP | 1 | LEHHHHHH | 8 | Unmodified | 0 | VOT16-1562 | 1 | 1181_R | 361.84 | 3 | 361.8375 | 1082.491 | 472 | 38.972 |
| GRO.DD.prfA+ | UAA-GFP | 1 | LEHHHHHH | 8 | Unmodified | 0 | VOT16-1562 | 1 | 1181_R | 361.84 | 3 | 361.8375 | 1082.491 | 1592 | 38.151 |
| GRO.DD.prfA+ | UAA-GFP | 1 | LEHHHHHH | 8 | Unmodified | 0 | VOT16-1562 | 1 | 1181_R | 361.84 | 3 | 361.8375 | 1082.491 | 2013 | 27.705 |
| GRO.DD.prfA+ | UAA-GFP | 1 | LEHHHHHH | 8 | Unmodified | 0 | VOT16-1562 | 1 | 1181_R | 361.84 | 3 | 361.8375 | 1082.491 | 3009 | 60.76 |
| GRO.DD.prfA+ | UAA-GFP | 1 | LEHHHHHH | 8 | Unmodified | 0 | VOT16-1562 | 1 | 1181_R | 361.84 | 3 | 361.8375 | 1082.491 | 3234 | 48.756 |
| GRO.DD.prfA+ | UAA-GFP | 1 | LEHHHHHH | 8 | Unmodified | 0 | VOT16-1562 | 1 | 1181_R | 361.84 | 3 | 361.8375 | 1082.491 | 3514 | 38.322 |
| GRO.DD | UAA-GFP | 1 | LEHHHHHH | 8 | Unmodified | 0 | VOT16-1565 | 1 | 1180_R | 361.84 | 3 | 361.8375 | 1082.491 | 508 | 25.45 |
| GRO.DD | UAA-GFP | 1 | LEHHHHHH | 8 | Unmodified | 0 | VOT16-1565 | 1 | 1180_R | 361.84 | 3 | 361.8375 | 1082.491 | 3474 | 19.36 |
| GRO.DD.prfA+_R | UAG-GFP | 1 | LEHHHHHH | 8 | Unmodified | 0 | VOT16-1568 | 1 | 1171_R | 361.84 | 3 | 361.8375 | 1082.491 | 489 | 49.65 |
| GRO.DD.prfA+_R | UAG-GFP | 1 | LEHHHHHH | 8 | Unmodified | 0 | VOT16-1568 | 1 | 1171_R | 361.84 | 3 | 361.8375 | 1082.491 | 1118 | 37.646 |
| GRO.DD | UAG-GFP | N/A | LEHHHHHHYQR | 11 | Unmodified | 0 | VOT16-1571 | 1 | 1170_R | 510.91 | 3 | 510.9118 | 1529.714 | 1161 | 151.22 |
| GRO.DD | UAG-GFP | N/A | LEHHHHHHYQR | 11 | Unmodified | 0 | VOT16-1571 | 1 | 1170_R | 383.44 | 4 | 383.4357 | 1529.714 | 1298 | 80.24 |
| GRO.DD | UAG-GFP | N/A | LEHHHHHHQQR | 11 | Unmodified | 0 | VOT16-1571 | 1 | 1170_R | 374.68 | 4 | 374.6845 | 1494.709 | 1057 | 77.662 |
| GRO.DD | UAG-GFP | 3 (+8 shift, I mislabled as L) | LEHHHHHHSGL | 11 | Unmodified | 0 | VOT16-1571 | 1 | 1170_R | 447.55 | 3 | 447.55 | 1339.628 | 1299 | 67.207 |
| GRO.DD | UAG-GFP | 2 (+19 frameshift) | LEHHHHHHMVR | 11 | Unmodified | 0 | VOT16-1542 | 1 | 1170 | 368.18 | 4 | 368.1825 | 1468.701 | 1710 | 57.149 |
| GRO.DD | UAG-GFP | N/A | LEHHHHHHQLD | 11 | Unmodified | 0 | VOT16-1542 | 1 | 1170 | 360.67 | 4 | 360.6723 | 1438.66 | 1751 | 56.404 |
| GRO.DD | UAG-GFP | 3 (possible I/L suppression of UAG, then +2 frameshift) | LEHHHHHHLGR | 11 | Unmodified | 0 | VOT16-1571 | 1 | 1170_R | 353.18 | 4 | 353.1816 | 1408.697 | 1286 | 47.302 |
| GRO.DD | UAG-GFP | 2 (+19 frameshift, MS/MS cannot distinguish between VMR and MVR) | LEHHHHHHVMR | 11 | Unmodified | 0 | VOT16-1542 | 1 | 1170 | 490.57 | 3 | 490.5742 | 1468.701 | 1711 | 47.302 |
| GRO.DD | UAG-GFP | N/A | LEHHHHHHYQR | 11 | Unmodified | 0 | VOT16-1542 | 1 | 1170 | 383.44 | 4 | 383.4357 | 1529.714 | 1766 | 46.406 |
| GRO.DD | UAG-GFP | 2 (+16 frameshift, MS/MS cannot distinguish between VM and MV) | LEHHHHHHPVM | 11 | Unmodified | 0 | VOT16-1571 | 1 | 1170_R | 470.89 | 3 | 470.8914 | 1409.652 | 1608 | 43.297 |
| GRO.DD | UAG-GFP | 1 (suppression of UAG with V, then +3 to translate AR) | LEHHHHHHVAR | 11 | Unmodified | 0 | VOT16-1571 | 1 | 1170_R | 470.91 | 3 | 470.5731 | 1408.697 | 1309 | 41.399 |
| GRO.DD | UAG-GFP | N/A | LEHHHHHHEKP | 11 | Unmodified | 1 | VOT16-1542 | 1 | 1170 | 479.9 | 3 | 479.9009 | 1436.681 | 1856 | 41.08 |
| GRO.DD | UAG-GFP | 2 (+19 frameshift) | LEHHHHHHMVR | 11 | Unmodified | 0 | VOT16-1571 | 1 | 1170_R | 368.18 | 4 | 368.1825 | 1468.701 | 1389 | 40.883 |
| GRO.DD | UAG-GFP | 3 (+2 frameshift) | LEHHHHHHGR | 10 | Unmodified | 0 | VOT16-1571 | 1 | 1170_R | 324.91 | 4 | 324.9106 | 1295.613 | 1044 | 38.754 |
| GRO.DD | UAG-GFP | 2 (+16 frameshift) | LEHHHHHHPM | 10 | Unmodified | 0 | VOT16-1571 | 1 | 1170_R | 437.87 | 3 | 437.8686 | 1310.584 | 1330 | 38.303 |
| GRO.DD | UAG-GFP | 2 (suppression with Q, +1 frameshift to R) | LEHHHHHHQR | 10 | Unmodified | 0 | VOT16-1571 | 1 | 1170_R | 342.67 | 4 | 342.6699 | 1366.65 | 1049 | 37.344 |
| GRO.DD | UAG-GFP | N/A | LEHHHHHHEKP | 11 | Unmodified | 1 | VOT16-1571 | 1 | 1170_R | 479.9 | 3 | 479.9009 | 1436.681 | 1588 | 35.71 |
| GRO.DD | UAG-GFP | N/A | LEHHHHHHQLD | 11 | Unmodified | 0 | VOT16-1571 | 1 | 1170_R | 360.67 | 4 | 360.6723 | 1438.66 | 1444 | 34.755 |
| GRO.DD | UAG-GFP | N/A | LEHHHHHHEKP | 11 | Unmodified | 1 | VOT16-1571 | 1 | 1170_R | 360.18 | 4 | 360.1775 | 1436.681 | 1597 | 33.875 |
| GRO.DD | UAG-GFP | N/A | LEHHHHHHSLK | 11 | Unmodified | 0 | VOT16-1571 | 1 | 1170_R | 353.93 | 4 | 353.6827 | 1410.702 | 1147 | 32.051 |

**Table S2: Manually-verified C-terminal fragments**

| **Strain** | **GFP** | **File #** | **Manual verification score (0 = not valid, 1 = valid)** | **Sequence** | **Length** | **Experiment** | **MS/MS m/z** | **Charge** | **m/z** | **Mass** | **Number of data points** | **Number of scans** | **MS/MS Scan Number** | **Score** |
| --- | --- | --- | --- | --- | --- | --- | --- | --- | --- | --- | --- | --- | --- | --- |
| GRO.DD | UAG-GFP | 1 | 0 | HAANDENYALDD | 12 | 1170_R | 674.28 | 2 | 674.276 | 1346.537 | 146 | 44 | 3171 | 120.32 |
| GRO.DD | UAG-GFP | 2 | 1 | LEHHHHHHAAAANDENYALDD | 21 | 1170_R | 806.69 | 3 | 806.3516 | 2416.033 | 29 | 12 | 1968 | 65.423 |
| GRO.DD | UAG-GFP | 3 | 1 | LEHHHHHHAANDENYALDD | 19 | 1170_R | 1138.48 | 2 | 1137.987 | 2273.959 | 30 | 8 | 1843 | 146.78 |
| GRO.DD | UAG-GFP | 4 | 1 | LEHHHHHHALGD | 12 | 1170 | 360.67 | 4 | 360.6723 | 1438.66 | 9 | 4 | 1751 | 60.691 |
| GRO.DD | UAG-GFP | 5 | 1 | LEHHHHHHALGDPMVR | 16 | 1170_R | 641.98 | 3 | 641.6483 | 1921.923 | 65 | 18 | 1808 | 121.82 |
| GRO.DD | UAG-GFP | 6 | 0 | LEHHHHHHASGIP | 13 | 1170 | 503.58 | 3 | 503.58 | 1507.718 | 11 | 6 | 1846 | 15.941 |
| GRO.DD | UAG-GFP | 7 | 0 | LEHHHHHHGAANDENYALDD | 20 | 1170_R | 582.97 | 4 | 583.7523 | 2330.98 | 15 | 7 | 1832 | 31.41 |
| GRO.DD | UAG-GFP | 8 | 0 | LEHHHHHHGALGDPMVR | 17 | 1170_R | 494.97 | 4 | 495.7434 | 1978.945 | 6 | 3 | 1888 | 18.58 |
| GRO.DD | UAG-GFP | 9 | 1 | LEHHHHHHGDAANDENYALDD | 21 | 1170 | 612.51 | 4 | 612.5091 | 2446.007 | 26 | 8 | 2210 | 63.302 |
| GRO.DD | UAG-GFP | 10 | 1 | LEHHHHHHGDPMVR | 14 | 1170 | 580.28 | 3 | 580.2746 | 1737.802 | 70 | 23 | 1785 | 159.12 |
| GRO.DD | UAG-GFP | 11 | 0 | LEHHHHHHGR | 10 | 1170_R | 324.91 | 4 | 324.9106 | 1295.613 | 71 | 32 | 1044 | 38.754 |
| GRO.DD | UAG-GFP | 12 | 0 | LEHHHHHHGSGIPWYAC | 17 | 1170 | 519.74 | 4 | 519.4855 | 2073.913 | 40 | 11 | 3771 | 15.273 |
| GRO.DD | UAG-GFP | 13 | 1 | LEHHHHHHHAANDENYALDD | 20 | 1170_R | 805.01 | 3 | 804.6798 | 2411.018 | 8 | 3 | 1786 | 99.044 |
| GRO.DD | UAG-GFP | 14 | 1 | LEHHHHHHHHAANDENYALDD | 21 | 1170_R | 638.53 | 4 | 638.0264 | 2548.077 | 20 | 7 | 1770 | 72.359 |
| GRO.DD | UAG-GFP | 15 | 1 | LEHHHHHHLGDPMVR | 15 | 1170_R | 618.3 | 3 | 617.9693 | 1850.886 | 18 | 5 | 1777 | 97.767 |
| GRO.DD | UAG-GFP | 16 | 1 | LEHHHHHHLGR | 11 | 1170_R | 353.18 | 4 | 353.1816 | 1408.697 | 261 | 78 | 1286 | 47.302 |
| GRO.DD | UAG-GFP | 17 | 0 | LEHHHHHHLSGI | 12 | 1170 | 485.24 | 3 | 485.2447 | 1452.712 | 19 | 5 | 1726 | 14.009 |
| GRO.DD | UAG-GFP | 18 | 0 | LEHHHHHHNRG | 11 | 1170_R | 470.89 | 3 | 470.8927 | 1409.656 | 21 | 8 | 1608 | 33.87 |
| GRO.DD | UAG-GFP | 19 | 1 | LEHHHHHHQALGDPMVR | 17 | 1170_R | 513.5 | 4 | 513.5027 | 2049.982 | 71 | 20 | 1857 | 79.837 |
| GRO.DD | UAG-GFP | 20 | 0 | LEHHHHHHQGAAANDENYALDD | 22 | 1170_R | 633.78 | 4 | 633.5262 | 2530.076 | 24 | 9 | 1955 | 32.797 |
| GRO.DD | UAG-GFP | 21 | 1 | LEHHHHHHQGAR | 12 | 1170_R | 374.68 | 4 | 374.6845 | 1494.709 | 94 | 33 | 1057 | 70.942 |
| GRO.DD | UAG-GFP | 22 | 0 | LEHHHHHHQR | 10 | 1170_R | 342.67 | 4 | 342.6699 | 1366.65 | 78 | 30 | 1049 | 37.344 |
| GRO.DD | UAG-GFP | 23 | 1 | LEHHHHHHSGAANDENYALDD | 21 | 1170 | 605.76 | 4 | 605.5103 | 2418.012 | 28 | 14 | 2156 | 73.138 |
| GRO.DD | UAG-GFP | 24 | 0 | LEHHHHHHSGIPAANDENYALDD | 23 | 1170 | 658.54 | 4 | 658.0445 | 2628.149 | 84 | 37 | 3264 | 9.5658 |
| GRO.DD | UAG-GFP | 25 | 1 | LEHHHHHHSGIPWYAC | 16 | 1170 | 673.64 | 3 | 673.3044 | 2016.891 | 77 | 30 | 3801 | 82.202 |
| GRO.DD | UAG-GFP | 26 | 0 | LEHHHHHHVAR | 11 | 1170_R | 470.91 | 3 | 470.5731 | 1408.697 | 140 | 51 | 1309 | 41.399 |
| GRO.DD | UAG-GFP | 27 | 1 | LEHHHHHHYGAR | 12 | 1170_R | 510.91 | 3 | 510.9118 | 1529.714 | 197 | 65 | 1161 | 141.1 |
| GRO.DD | UAG-GFP | 28 | 1 | LEHHHHHHGDPMVR | 14 | 1170 | 580.28 | 3 | 580.2746 | 1737.802 | 70 | 23 | 1785 | 159.12 |
| GRO.DD | UAG-GFP | 29 | 1 | LEHHHHHHAANDENYALDD | 19 | 1170_R | 1138.48 | 2 | 1137.987 | 2273.959 | 30 | 8 | 1843 | 146.78 |
| GRO.DD | UAG-GFP | 30 | 1 | LEHHHHHHYGAR | 12 | 1170_R | 510.91 | 3 | 510.9118 | 1529.714 | 197 | 65 | 1161 | 141.1 |
| GRO.DD | UAG-GFP | 31 | 1 | LEHHHHHHALGDPMVR | 16 | 1170 | 481.74 | 4 | 481.488 | 1921.923 | 155 | 38 | 2228 | 95.822 |
| GRO.DD | UAG-GFP | 32 | 1 | LEHHHHHHGDPMV | 13 | 1170 | 528.24 | 3 | 528.2409 | 1581.701 | 24 | 7 | 1911 | 91.313 |
| GRO.DD | UAG-GFP | 33 | 1 | LEHHHHHHGDPMVR | 14 | 1170_R | 435.46 | 4 | 435.4577 | 1737.802 | 143 | 34 | 1569 | 90.759 |
| GRO.DD | UAG-GFP | 34 | 1 | LEHHHHHHYQR | 11 | 1170_R | 510.91 | 3 | 510.9118 | 1529.714 | 197 | 65 | 1161 | 151.22 |
| GRO.DD | UAG-GFP | 35 | 1 | LEHHHHHHYQR | 11 | 1170_R | 383.44 | 4 | 383.4357 | 1529.714 | 350 | 92 | 1298 | 80.24 |
| GRO.DD | UAG-GFP | 36 | 1 | LEHHHHHHQQR | 11 | 1170_R | 374.68 | 4 | 374.6845 | 1494.709 | 94 | 33 | 1057 | 77.662 |
| GRO.DD | UAG-GFP | 37 | 1 | LEHHHHHHSGL | 11 | 1170_R | 447.55 | 3 | 447.55 | 1339.628 | 190 | 56 | 1299 | 67.207 |
| GRO.DD | UAG-GFP | 38 | 1 | LEHHHHHHMVR | 11 | 1170 | 368.18 | 4 | 368.1825 | 1468.701 | 65 | 24 | 1710 | 57.149 |
| GRO.DD | UAG-GFP | 39 | 0 | LEHHHHHHQLD | 11 | 1170 | 360.67 | 4 | 360.6723 | 1438.66 | 9 | 4 | 1751 | 56.404 |
| GRO.DD | UAG-GFP | 40 | 1 | LEHHHHHHLGR | 11 | 1170_R | 353.18 | 4 | 353.1816 | 1408.697 | 261 | 78 | 1286 | 47.302 |
| GRO.DD | UAG-GFP | 41 | 1 | LEHHHHHHVMR | 11 | 1170 | 490.57 | 3 | 490.5742 | 1468.701 | 34 | 14 | 1711 | 47.302 |
| GRO.DD | UAG-GFP | 42 | 1 | LEHHHHHHYQR | 11 | 1170 | 383.44 | 4 | 383.4357 | 1529.714 | 112 | 38 | 1766 | 46.406 |
| GRO.DD | UAG-GFP | 43 | 1 | LEHHHHHHPVM | 11 | 1170_R | 470.89 | 3 | 470.8914 | 1409.652 | 21 | 8 | 1608 | 43.297 |
| GRO.DD | UAG-GFP | 44 | 1 | LEHHHHHHVAR | 11 | 1170_R | 470.91 | 3 | 470.5731 | 1408.697 | 140 | 51 | 1309 | 41.399 |
| GRO.DD | UAG-GFP | 45 | 0 | LEHHHHHHEKP | 11 | 1170 | 479.9 | 3 | 479.9009 | 1436.681 | 36 | 12 | 1856 | 41.08 |
| GRO.DD | UAG-GFP | 46 | 1 | LEHHHHHHMVR | 11 | 1170_R | 368.18 | 4 | 368.1825 | 1468.701 | 74 | 21 | 1389 | 40.883 |

**Table S3: Doubling Times of Strain with or without Induction of GFP Protein Production, for Figures 3A and 3B**

| **Condition** | **Strain** | **GFP Stop Codon** | **Mean Doubling Time (min)** | **Standard Deviation Doubling Time** | **Max OD_600_** | **Standard Deviation Max OD_600_** | **% change in doubling time during GFP induction** | **% change in Max OD_600_ during GFP induction** |
| --- | --- | --- | --- | --- | --- | --- | --- | --- |
| no induction | ECNR2 | UAG | 49.09 | 1.59 | 1.803 | 0.0023 |  |  |
| no induction | GRO.AA | UAG | 70.76 | 4.55 | 1.610 | 0.0162 |  |  |
| no induction | GRO.AA.∆arfB | UAG | 62.29 | 5.23 | 1.601 | 0.0045 |  |  |
| no induction | GRO.AA.∆ssrA | UAG | 91.53 | 5.13 | 1.435 | 0.0296 |  |  |
| no induction | GRO.AA.∆arfA | UAG | 76.02 | 0.29 | 1.536 | 0.0652 |  |  |
| no induction | GRO.AA.∆ssrA.∆arfB | UAG | 94.97 | 1.24 | 1.244 | 0.0017 |  |  |
| no induction | GRO.AA.∆arfA.∆arfB | UAG | 81.69 | 0.96 | 1.444 | 0.0296 |  |  |
| no induction | GRO.AA | UAA | 79.04 | 0.57 | 1.484 | 0.0449 |  |  |
| induction of GFP | ECNR2 | UAG | 52.39 | 0.57 | 1.738 | 0.0053 | 6.72 | -3.59 |
| induction of GFP | GRO.AA | UAG | 109.18 | 3.18 | 1.489 | 0.0077 | 54.29 | -7.47 |
| induction of GFP | GRO.AA.∆arfB | UAG | 93.20 | 4.49 | 1.529 | 0.0050 | 49.63 | -4.51 |
| induction of GFP | GRO.AA.∆ssrA | UAG | 121.40 | 2.70 | 0.942 | 0.0124 | 32.64 | -34.35 |
| induction of GFP | GRO.AA.∆arfA | UAG | 111.75 | 3.74 | 1.504 | 0.0093 | 47.01 | -2.10 |
| induction of GFP | GRO.AA.∆ssrA.∆arfB | UAG | 106.79 | 8.02 | 0.481 | 0.0490 | 12.44 | -61.32 |
| induction of GFP | GRO.AA.∆arfA.∆arfB | UAG | 99.05 | 2.26 | 1.297 | 0.0211 | 21.25 | -10.16 |
| induction of GFP | GRO.AA | UAA | 87.09 | 0.89 | 1.281 | 0.0062 | 10.19 | -13.68 |

**Table S4: Conjugative efficiency of strains for plasmid RK2, For Figure 4A**

| **Donor** | **Recipient** | **Efficiency 1** | **Efficiency 2** | **Efficiency 3** | **Mean** | **StDev** |
| --- | --- | --- | --- | --- | --- | --- |
| NJM699 (ECNR2GIB.prfA+.ΔλRed [RK24]) | ECNR2 | 100 | 100 | 98.8372093 | 99.612403 | 0.5481448 |
| NJM699 (ECNR2GIB.prfA+.ΔλRed [RK24]) | GRO.AA | 87.20930233 | 83.72093023 | 90.69767442 | 87.209302 | 2.8482439 |
| NJM699 (ECNR2GIB.prfA+.ΔλRed [RK24]) | GRO.AA.∆arfB | 38.37209302 | 44.18604651 | 47.6744186 | 43.410853 | 3.8370135 |
| NJM699 (ECNR2GIB.prfA+.ΔλRed [RK24]) | GRO.AA.∆ssrA | 98.8372093 | 98.8372093 | 100 | 99.224806 | 0.5481448 |
| NJM699 (ECNR2GIB.prfA+.ΔλRed [RK24]) | GRO.AA.∆arfA | 58.13953488 | 70.93023256 | 65.11627907 | 64.728682 | 5.228968 |
| NJM699 (ECNR2GIB.prfA+.ΔλRed [RK24]) | GRO.AA.∆ssrA.∆arfB | 100 | 100 | 100 | 100 | 0 |
| NJM699 (ECNR2GIB.prfA+.ΔλRed [RK24]) | GRO.AA.∆arfA.∆arfB | 40.69767442 | 53.48837209 | 46.51162791 | 46.899225 | 5.228968 |

**Table S5: Doubling Times of Strains with and without conjugative plasmid RK2, for Figure 4B**

| **Condition** | **Strain** | **Mean Doubling Time (min)** | **Standard Deviation Doubling Time** | **Max OD_600_** | **Standard Deviation Max OD_600_** |
| --- | --- | --- | --- | --- | --- |
| no RK2 | ECNR2 | 53.60 | 0.22 | 1.217 | 0.0096 |
| no RK2 | GRO.AA | 62.19 | 0.99 | 1.085 | 0.0116 |
| no RK2 | GRO.AA.∆arfB | 60.97 | 0.64 | 1.115 | 0.0218 |
| no RK2 | GRO.AA.∆ssrA | 71.10 | 0.92 | 1.018 | 0.0162 |
| no RK2 | GRO.AA.∆arfA | 63.24 | 0.52 | 1.116 | 0.0214 |
| no RK2 | GRO.AA.∆ssrA.∆arfB | 71.78 | 1.06 | 1.026 | 0.0112 |
| no RK2 | GRO.AA.∆arfA.∆arfB | 65.02 | 0.72 | 1.148 | 0.0181 |
| with RK2 | ECNR2 | 55.12 | 1.04 | 1.190 | 0.0121 |
| with RK2 | GRO.AA | 79.61 | 1.65 | 0.886 | 0.0074 |
| with RK2 | GRO.AA.∆arfB | 84.22 | 6.62 | 0.784 | 0.0263 |
| with RK2 | GRO.AA.∆ssrA | 75.77 | 0.53 | 0.959 | 0.0112 |
| with RK2 | GRO.AA.∆arfA | 79.93 | 2.46 | 0.863 | 0.0225 |
| with RK2 | GRO.AA.∆ssrA.∆arfB | 78.95 | 1.11 | 0.938 | 0.0119 |
| with RK2 | GRO.AA.∆arfA.∆arfB | 117.99 | 3.61 | 0.686 | 0.0134 |

**Table S6: Quantification of conjugation events for F plasmid, for Figure 4C**

| **Donor** | **Recipient** | **S1** | **S2** | **S3** | **Mean** | **StDev** |
| --- | --- | --- | --- | --- | --- | --- |
| ECNR2 | NJM1 (ECNR2) | 8.52E+08 | 8.49E+08 | 9.99E+08 | 900000000 | 70014284 |
| GRO.AA | NJM1 (ECNR2) | 3.11E+04 | 3.37E+04 | 3.43E+04 | 33033.333 | 1388.8444 |
| GRO.AA.∆arfB | NJM1 (ECNR2) | 3.61E+04 | 3.62E+04 | 3.17E+04 | 34666.667 | 2098.1473 |
| GRO.AA.∆ssrA | NJM1 (ECNR2) | 2.85E+07 | 3.40E+07 | 4.42E+07 | 35566667 | 6504528.3 |
| GRO.AA.∆arfA | NJM1 (ECNR2) | 3.54E+04 | 3.22E+04 | 3.46E+04 | 34066.667 | 1359.7385 |
| GRO.AA.∆ssrA.∆arfB | NJM1 (ECNR2) | 5.00E+06 | 7.20E+06 | 7.80E+06 | 6666666.7 | 1203698 |
| GRO.AA.∆arfA.∆arfB | NJM1 (ECNR2) | 1.52E+04 | 1.54E+04 | 2.06E+04 | 17066.667 | 2499.7778 |

**Table S7: Relative titers of phage λ, for Figure 4D**

| **Phage** | **Strains (DB #)** | **TITER1** | **TITER2** | **TITER3** | **MEAN** |
| --- | --- | --- | --- | --- | --- |
| Lambda cI857 (#102) | ECNR2 | 2.60E+08 | 2.70E+08 | 4.80E+08 | 3.37E+08 |
| Lambda cI857 (#102) | GRO.AA | 0.00E+00 | 0.00E+00 | 0.00E+00 | 0.00E+00 |
| Lambda cI857 (#102) | GRO.AA.∆arfB | 0.00E+00 | 0.00E+00 | 0.00E+00 | 0.00E+00 |
| Lambda cI857 (#102) | GRO.AA.∆ssrA | 4.00E+08 | 5.50E+08 | 3.60E+08 | 4.37E+08 |
| Lambda cI857 (#102) | GRO.AA.∆arfA | 0.00E+00 | 0.00E+00 | 0.00E+00 | 0.00E+00 |
| Lambda cI857 (#102) | GRO.AA.∆ssrA.∆arfB | 5.00E+08 | 5.20E+08 | 4.40E+08 | 4.87E+08 |
| Lambda cI857 (#102) | GRO.AA.∆arfA.∆arfB | 0.00E+00 | 0.00E+00 | 0.00E+00 | 0.00E+00 |
