# Supplementary material for: Organisms with alternative genetic codes resolve unassigned codons via mistranslation and ribosomal rescue
Source: eLife. 2018 Oct 30;7:e34878. doi: 10.7554/eLife.34878 (PMC6207430; doi:10.7554/eLife.34878)

Raw file	Scan	Method	Score	m/z
VOT16-1571	1044	FTMS; HCD	38.75	324.91

Figure 2 – Supplement File 1
Peptides detected through mass spectrometry
Strain: GRO.DD
Plasmid: [UAG-GFP]
Spectrum 1

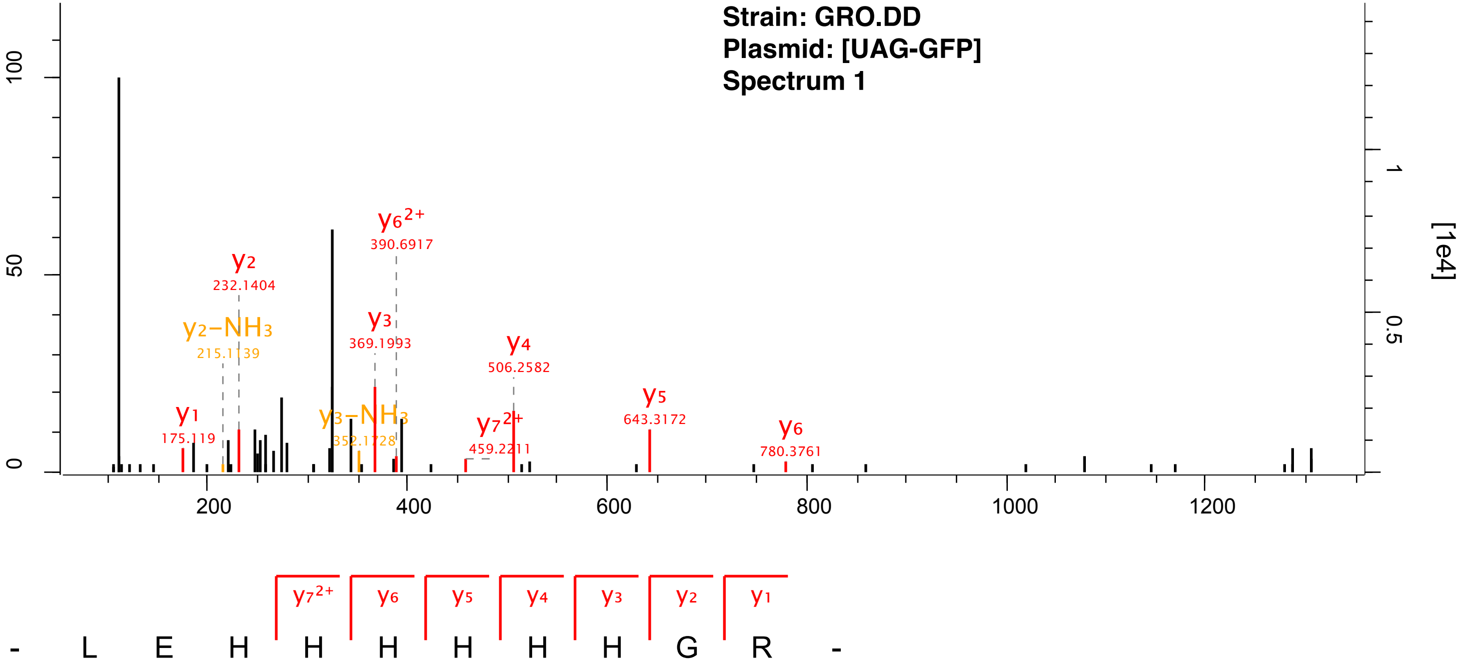

Figure 2 – Supplement File 1
Peptides detected through mass spectrometry
Strain: GRO.DD
Plasmid: [UAG-GFP]
Spectrum 2

Raw file	Scan	Method	Score	m/z
VOT16-1571	1049	FTMS; HCD	37.34	342.67

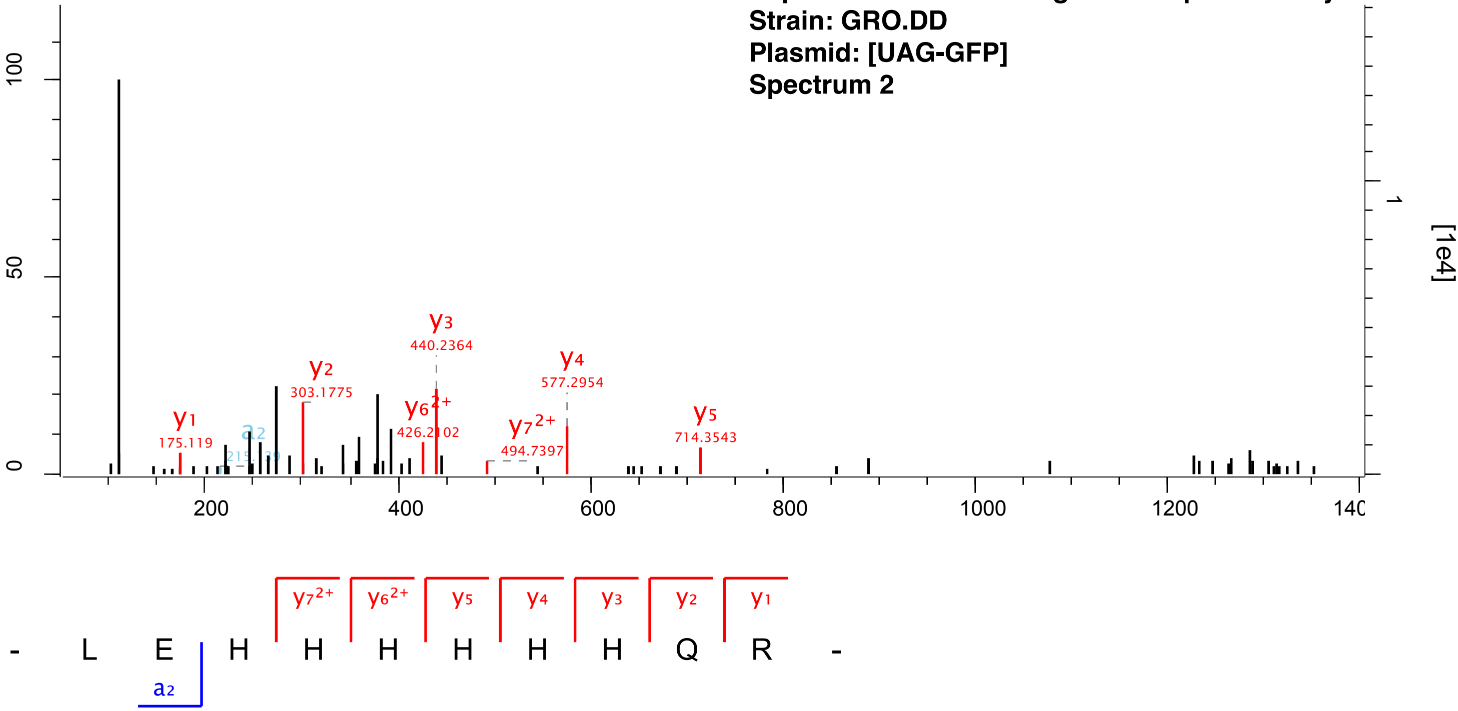

Raw file	Scan	Method	Score	m/z
VOT16-1571	1057	FTMS; HCD	77.66	374.68

Figure 2 – Supplement File 1
Peptides detected through mass spectrometry
Strain: GRO.DD
Plasmid: [UAG-GFP]
Spectrum 3

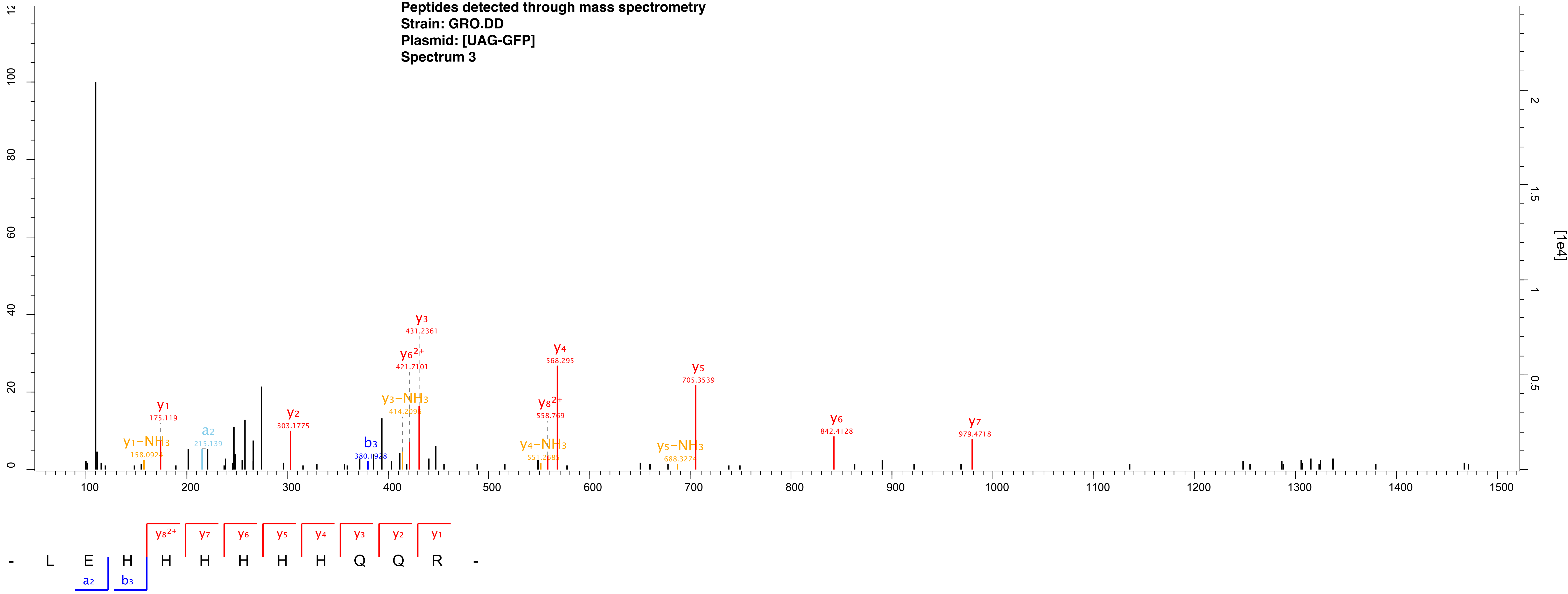

Raw file	Scan	Method	Score	m/z
VOT16-1571	1057	FTMS; HCD	70.94	374.68

Figure 2 – Supplement File 1
Peptides detected through mass spectrometry
Strain: GRO.DD
Plasmid: [UAG-GFP]
Spectrum 4

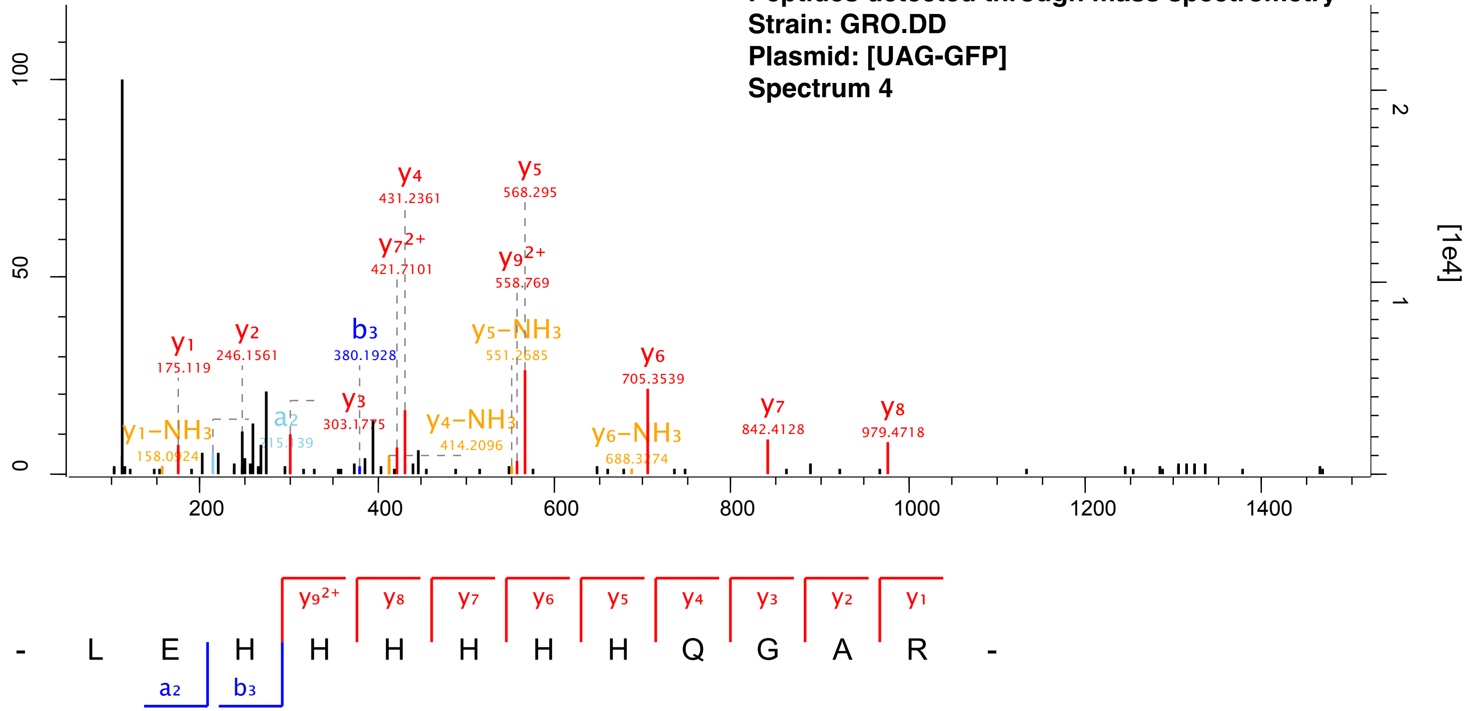

Raw file	Scan	Method	Score	m/z
VOT16-1571	1147	FTMS; HCD	32.05	353.93

Figure 2 – Supplement File 1
Peptides detected through mass spectrometry
Strain: GRO.DD
Plasmid: [UAG-GFP]
Spectrum 5

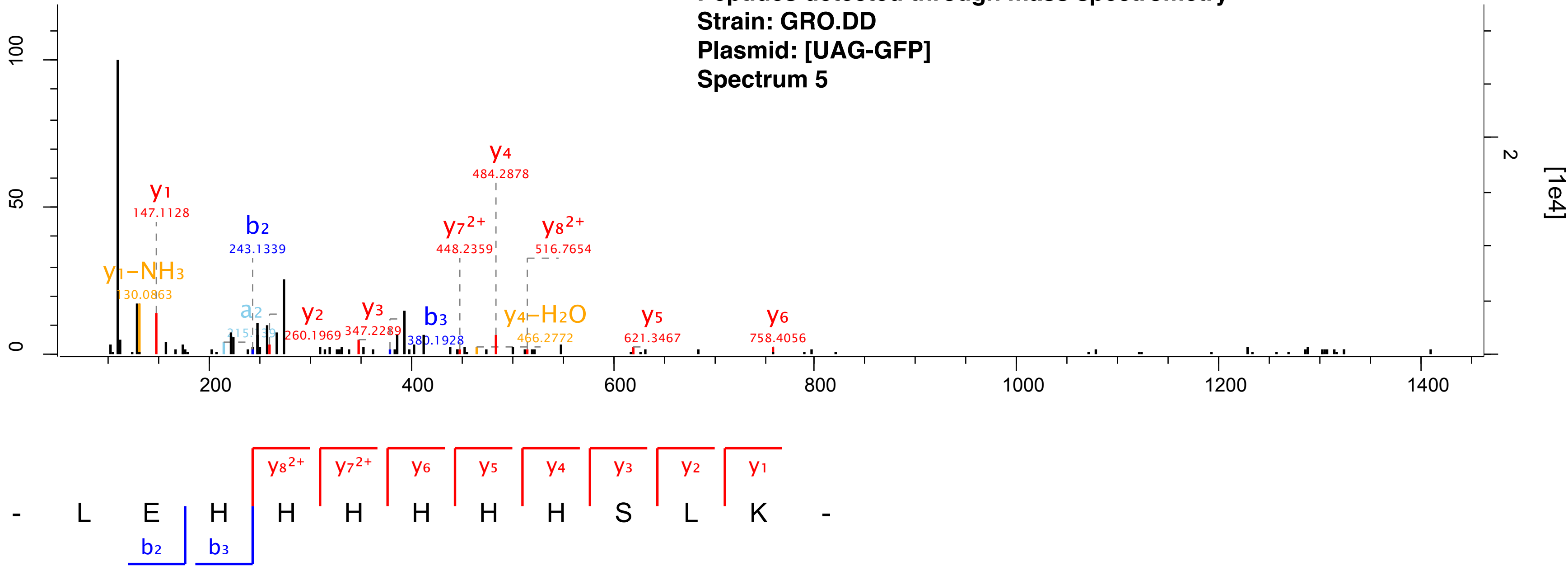

Raw file	Scan	Method	Score	m/z
VOT16-1571	1160	FTMS; HCD	62.8	383.44

Figure 2 – Supplement File 1
Peptides detected through mass spectrometry
Strain: GRO.DD
Plasmid: [UAG-GFP]
Spectrum 6

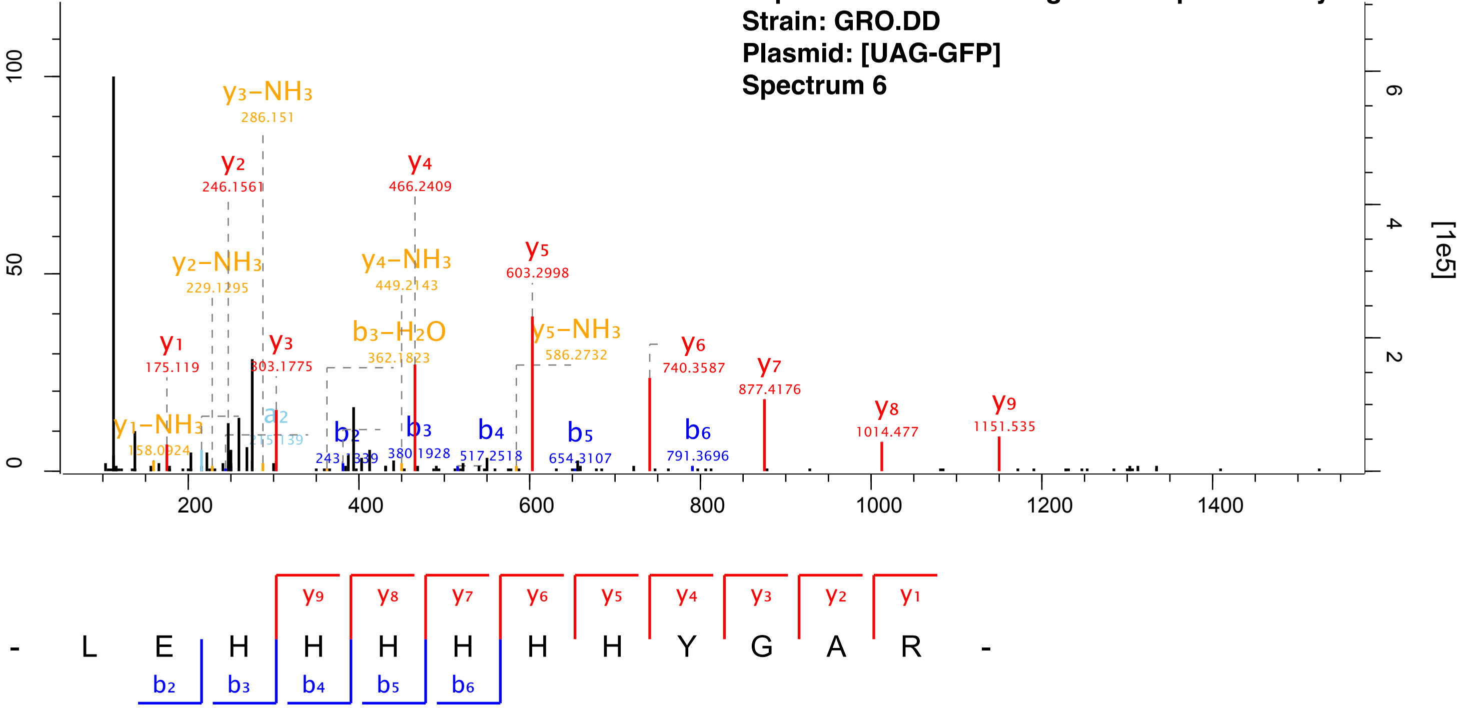

Raw file	Scan	Method	Score	m/z
VOT16-1571	1161	FTMS; HCD	151.22	510.91

Figure 2 – Supplement File 1
Peptides detected through mass spectrometry
Strain: GRO.DD
Plasmid: [UAG-GFP]
Spectrum 7

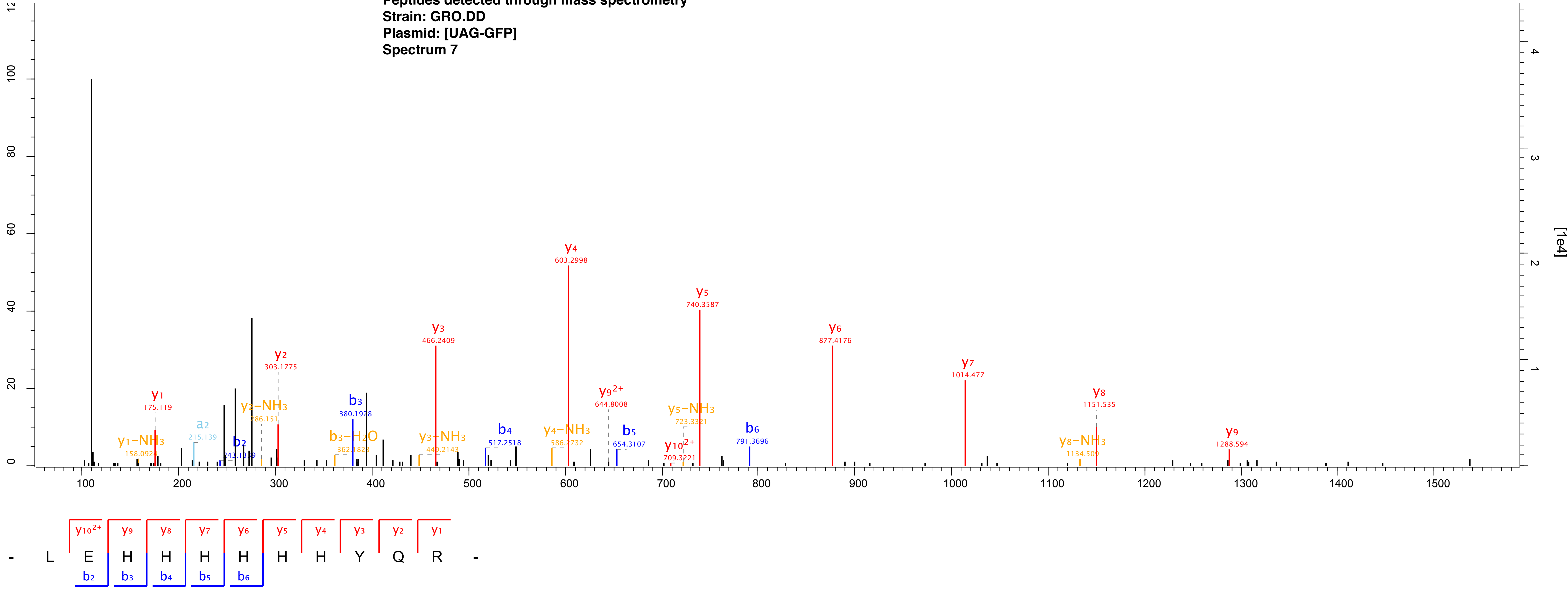

Raw file	Scan	Method	Score	m/z
VOT16-1571	1161	FTMS; HCD	141.1	510.91

Figure 2 – Supplement File 1
Peptides detected through mass spectrometry
Strain: GRO.DD
Plasmid: [UAG-GFP]
Spectrum 8

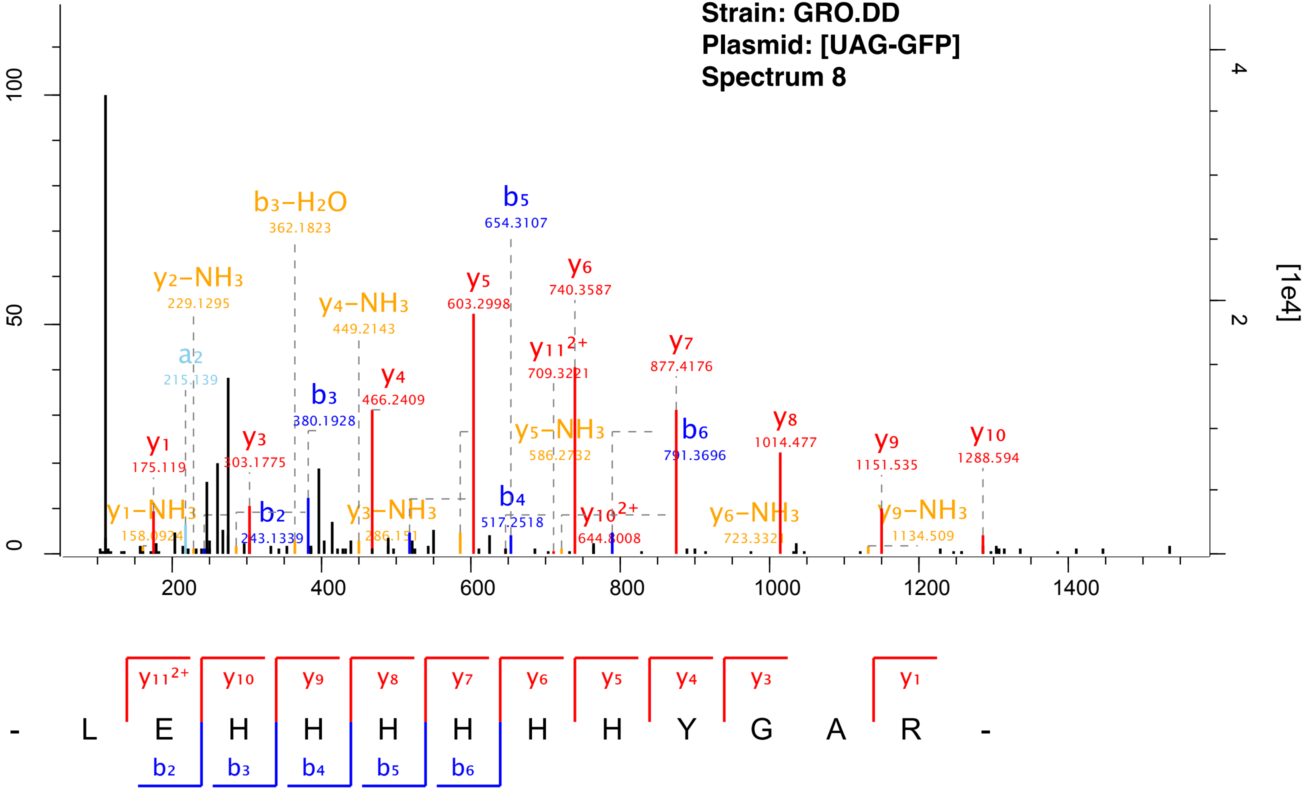

Raw file	Scan	Method	Score	m/z
VOT16-1571	1286	FTMS; HCD	47.3	353.18

Figure 2 – Supplement File 1
Peptides detected through mass spectrometry
Strain: GRO.DD
Plasmid: [UAG-GFP]
Spectrum 9

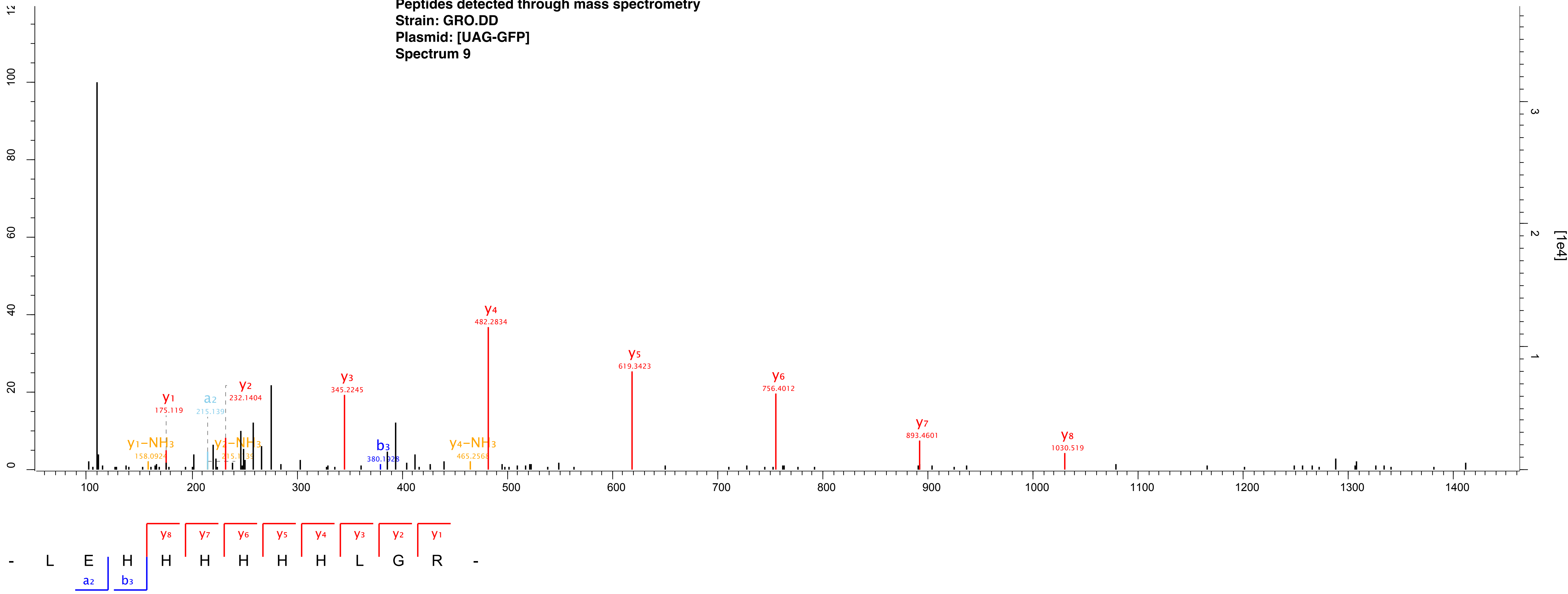

Raw file	Scan	Method	Score	m/z
VOT16-1571	1286	FTMS; HCD	47.3	353.18

Figure 2 – Supplement File 1
Peptides detected through mass spectrometry
Strain: GRO.DD
Plasmid: [UAG-GFP]
Spectrum 10

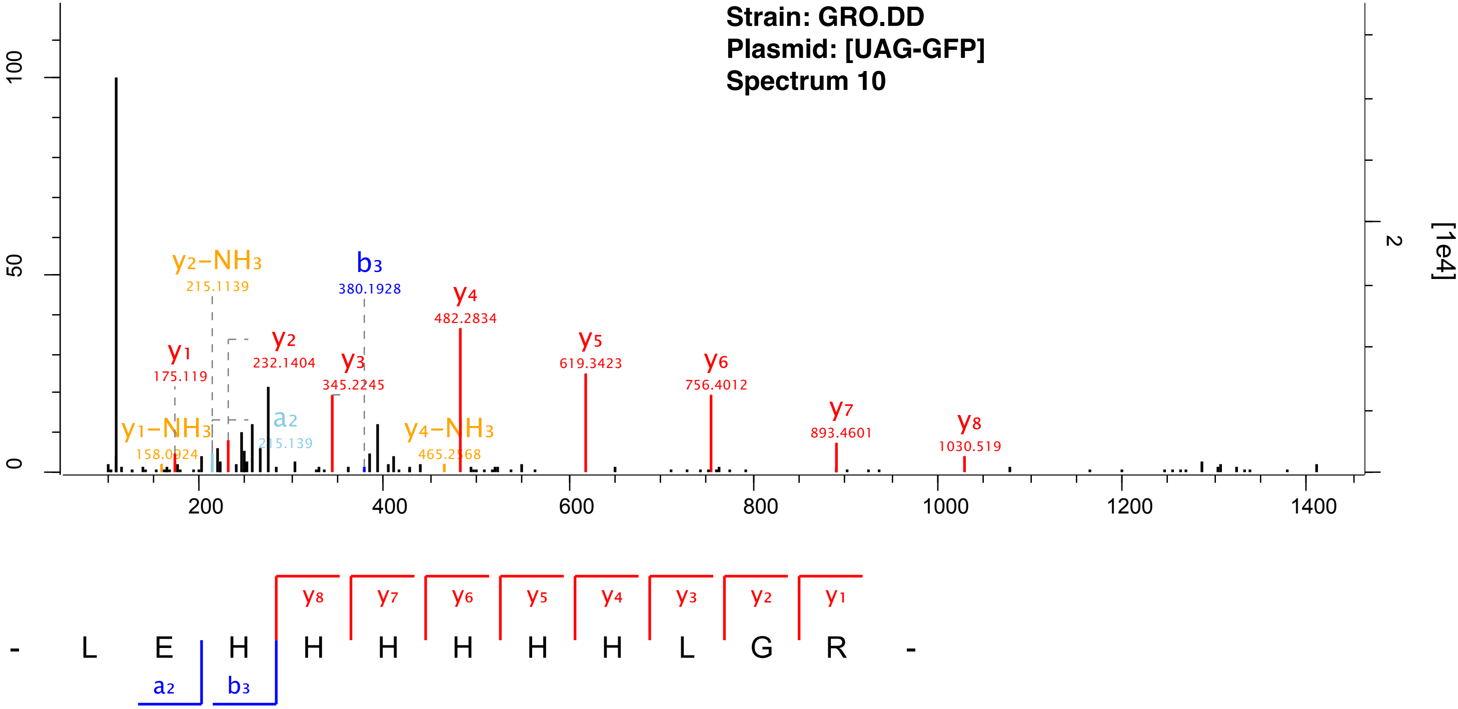

Raw file	Scan	Method	Score	m/z
VOT16-1571	1298	FTMS; HCD	80.24	383.44

Figure 2 – Supplement File 1
Peptides detected through mass spectrometry
Strain: GRO.DD
Plasmid: [UAG-GFP]
Spectrum 11

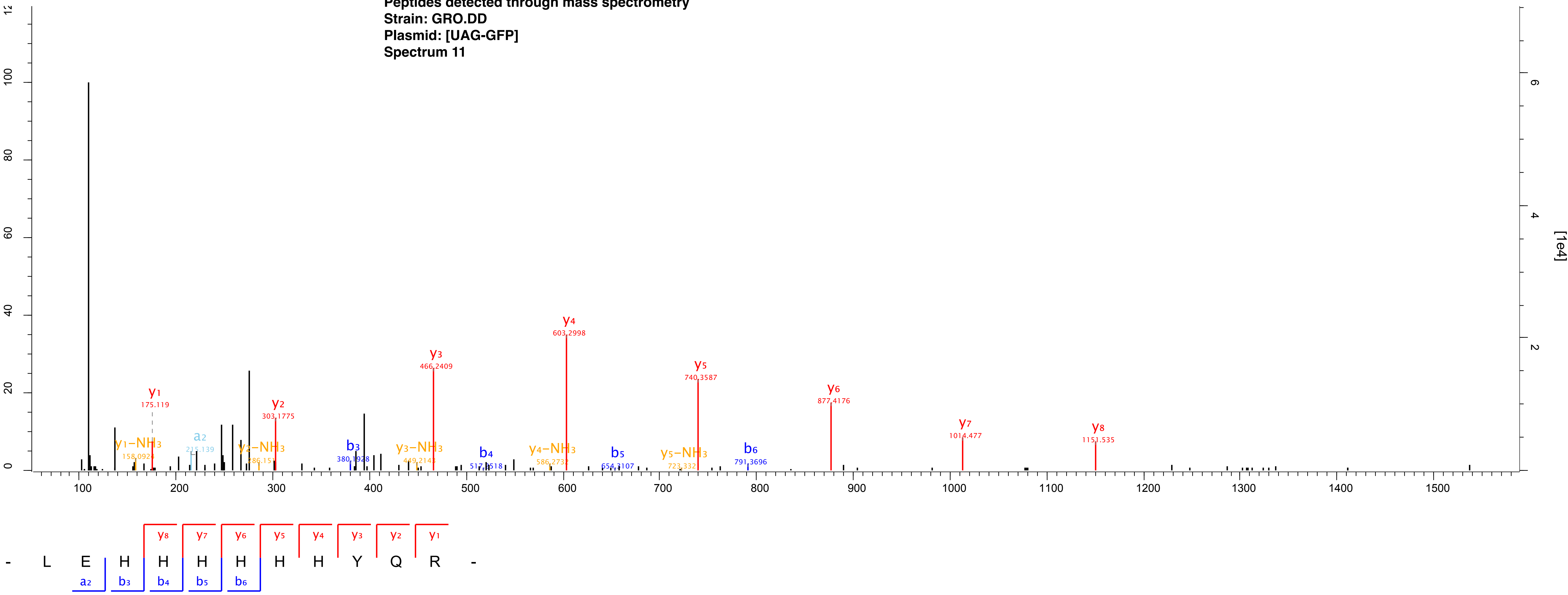

Raw file	Scan	Method	Score	m/z
VOT16-1571	1299	FTMS; HCD	67.21	447.55

Figure 2 – Supplement File 1
Peptides detected through mass spectrometry
Strain: GRO.DD
Plasmid: [UAG-GFP]
Spectrum 12

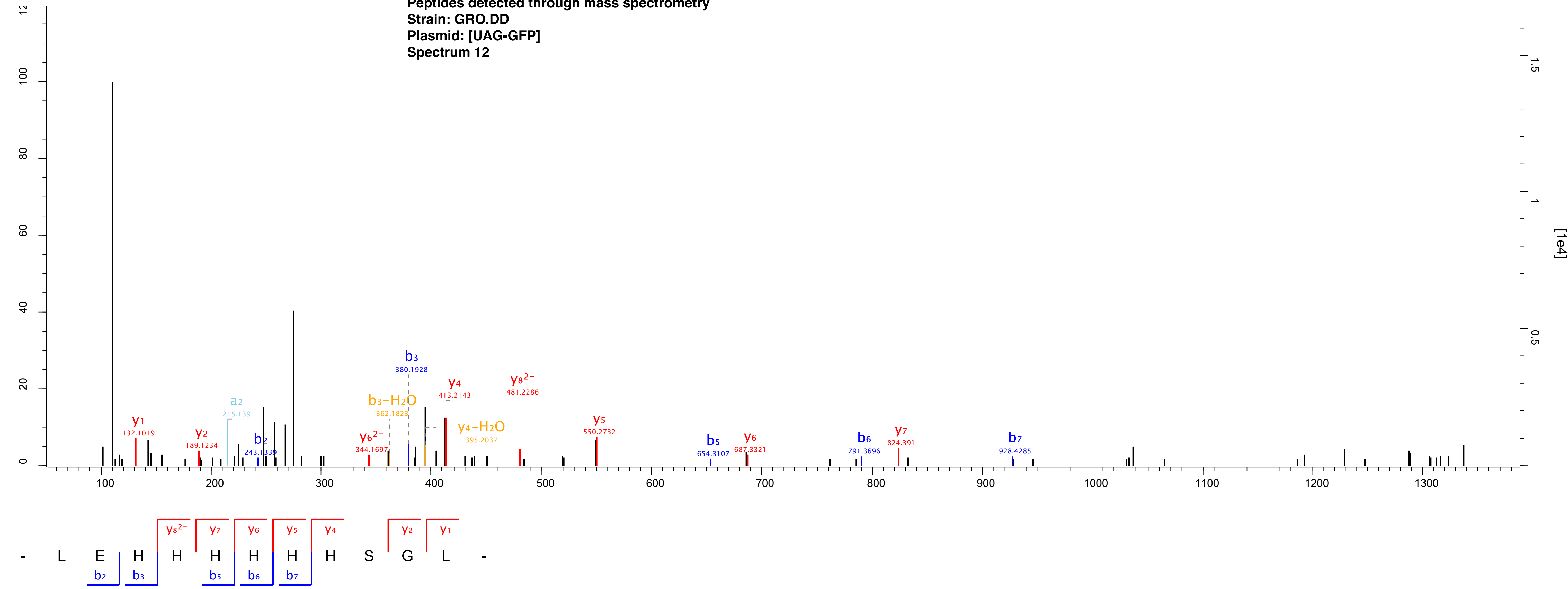

Raw file	Scan	Method	Score	m/z
VOT16-1571	1309	FTMS; HCD	41.4	470.91

Figure 2 – Supplement File 1
Peptides detected through mass spectrometry
Strain: GRO.DD
Plasmid: [UAG-GFP]
Spectrum 13

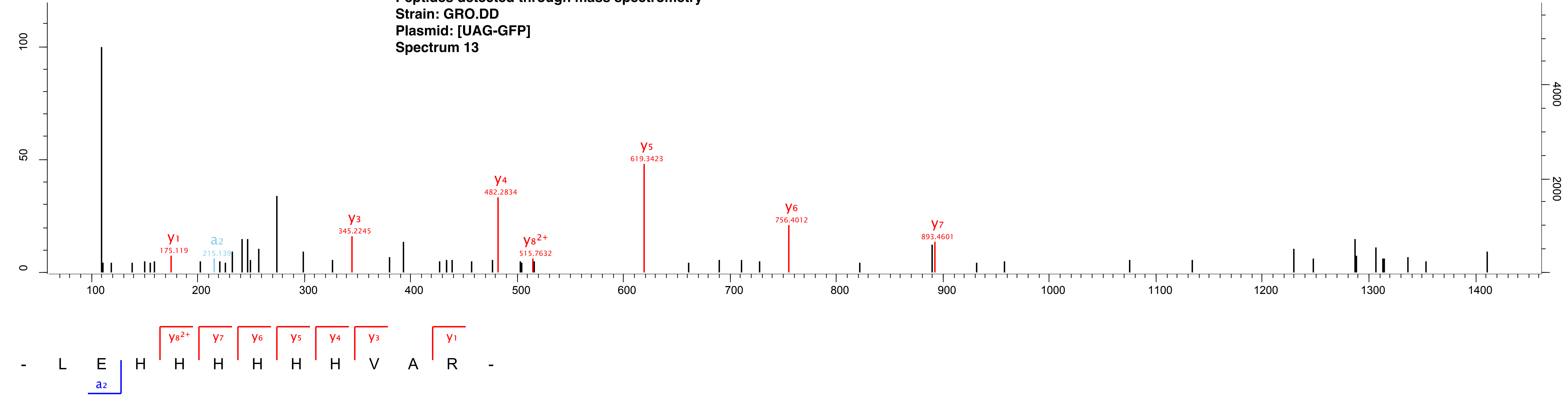

Raw file	Scan	Method	Score	m/z
VOT16-1571	1309	FTMS; HCD	41.4	470.91

Figure 2 – Supplement File 1
Peptides detected through mass spectrometry
Strain: GRO.DD
Plasmid: [UAG-GFP]
Spectrum 14

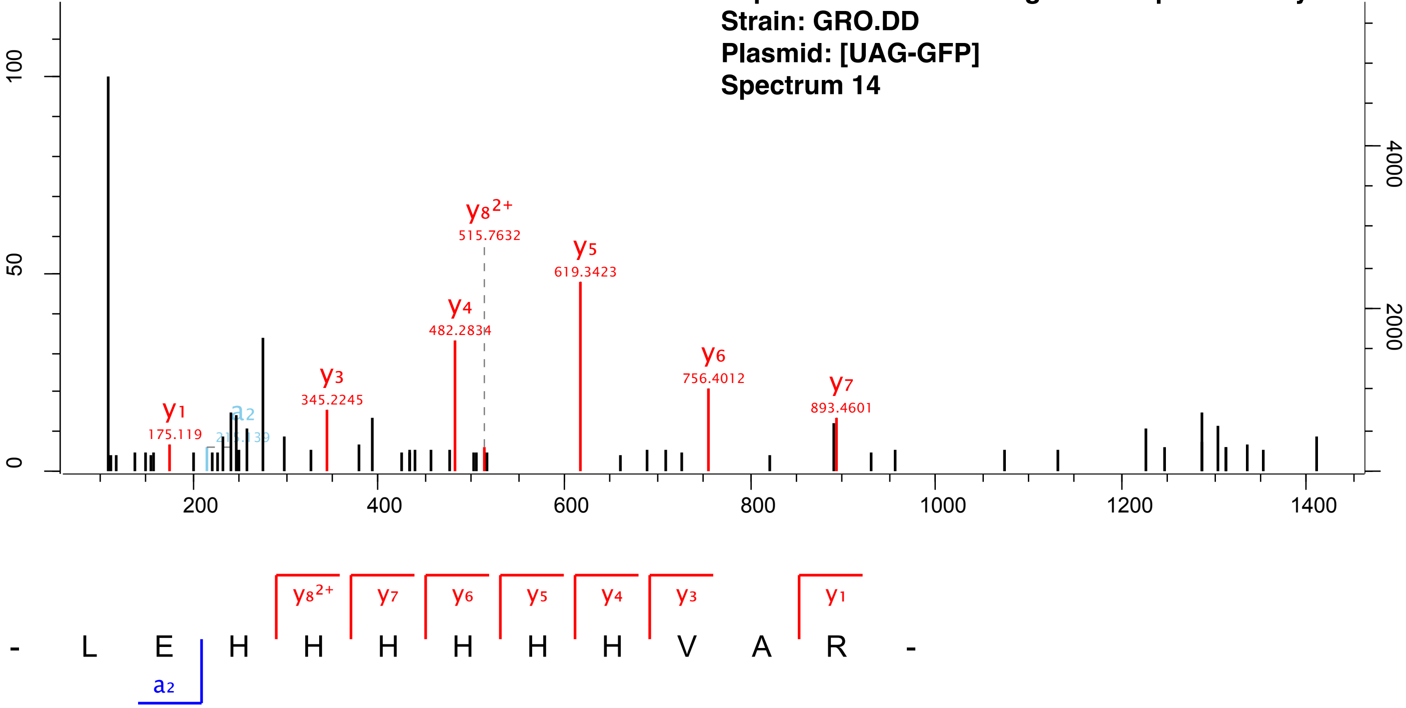

Raw file	Scan	Method	Score	m/z
VOT16-1571	1389	FTMS; HCD	40.88	368.18

Figure 2 – Supplement File 1
Peptides detected through mass spectrometry
Strain: GRO.DD
Plasmid: [UAG-GFP]
Spectrum 15

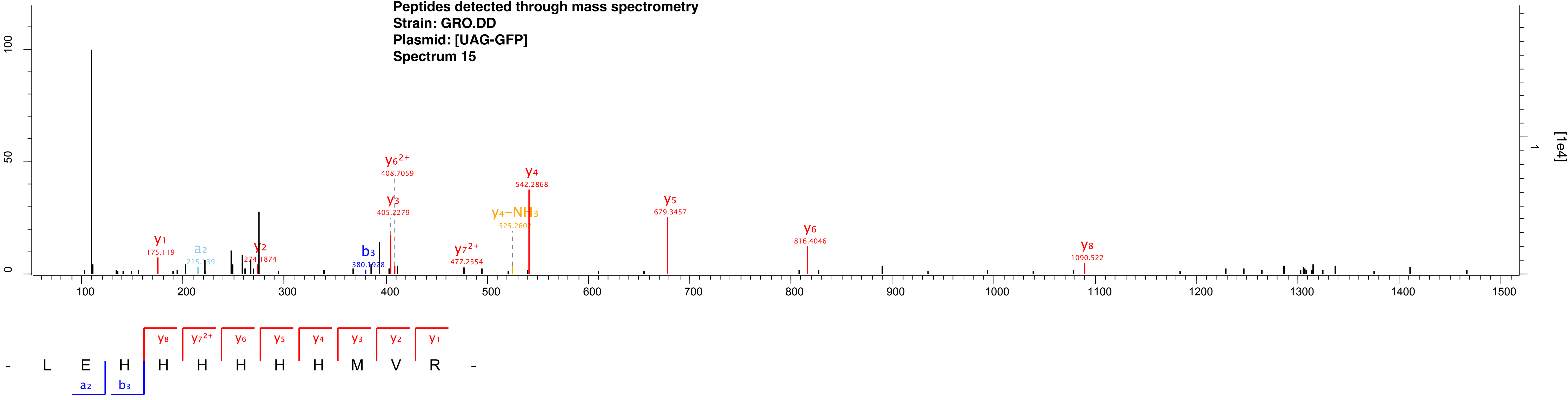

Raw file	Scan	Method	Score	m/z
VOT16-1571	1569	FTMS; HCD	90.76	435.46

Figure 2 – Supplement File 1
Peptides detected through mass spectrometry
Strain: GRO.DD
Plasmid: [UAG-GFP]
Spectrum 16

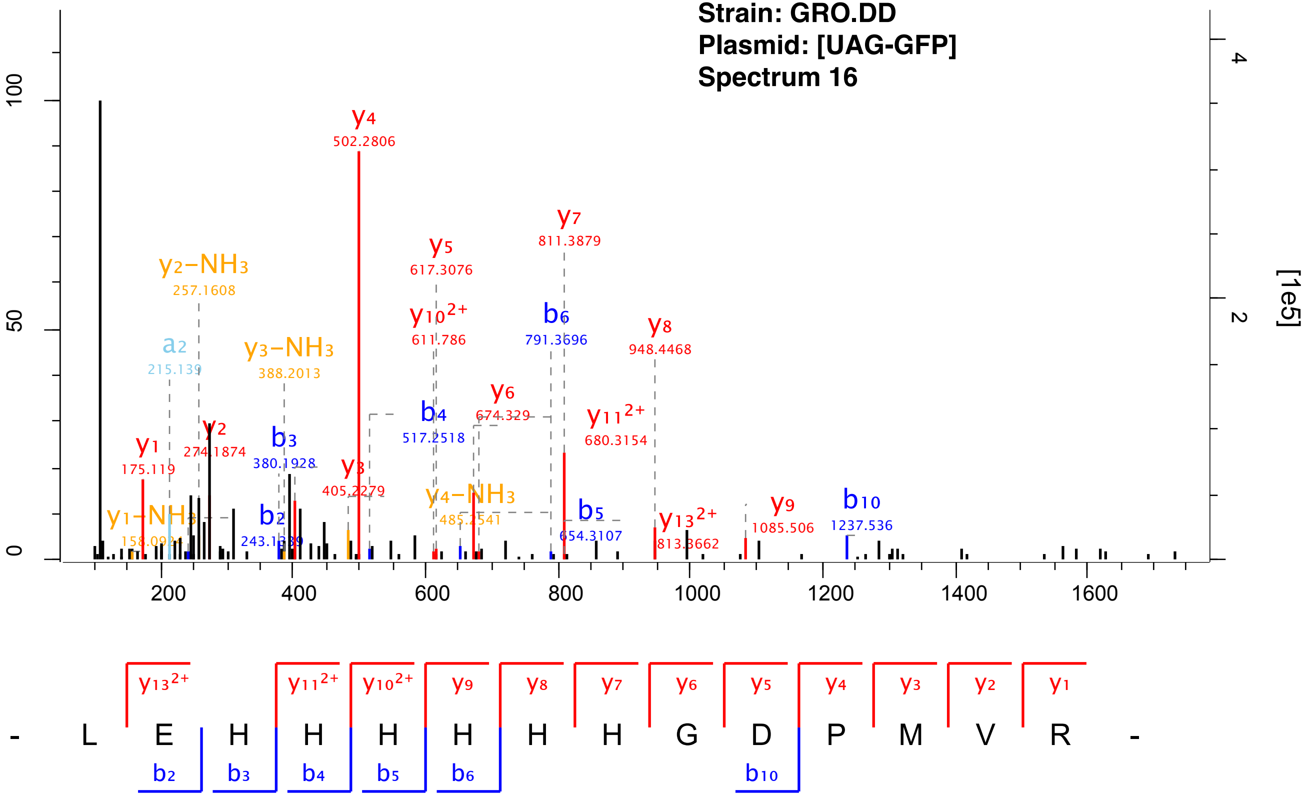

Raw file	Scan	Method	Score	m/z
VOT16-1571	1608	FTMS; HCD	43.3	470.89

Figure 2 – Supplement File 1
Peptides detected through mass spectrometry
Strain: GRO.DD
Plasmid: [UAG-GFP]
Spectrum 17

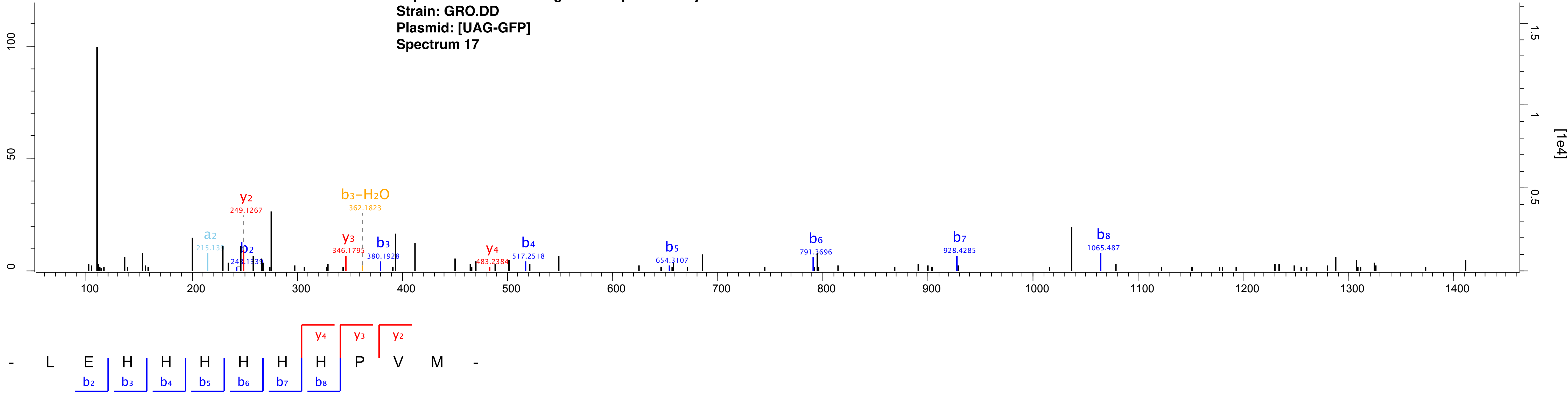

Raw file	Scan	Method	Score	m/z
VOT16-1571	1608	FTMS; HCD	33.87	470.89

Figure 2 – Supplement File 1
Peptides detected through mass spectrometry
Strain: GRO.DD
Plasmid: [UAG-GFP]
Spectrum 18

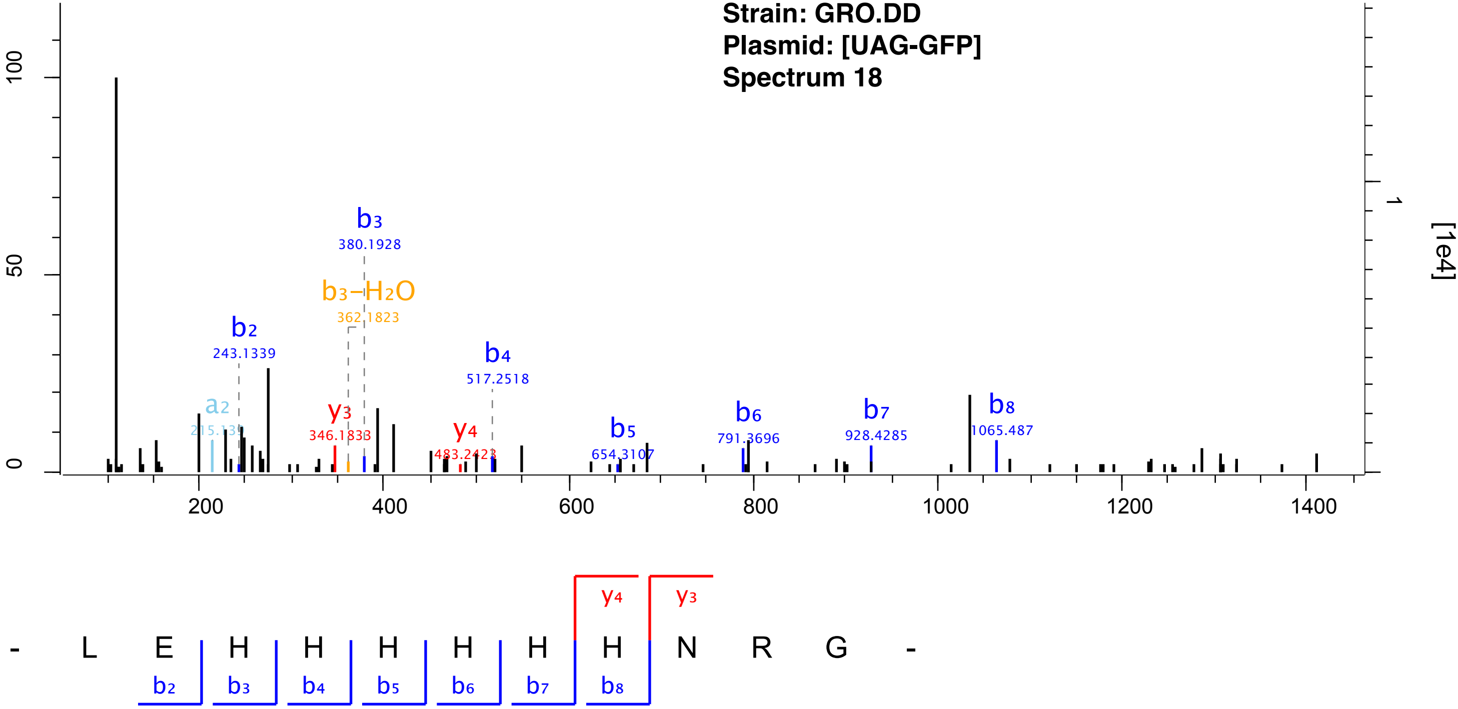

Raw file	Scan	Method	Score	m/z
VOT16-1542	1710	FTMS; HCD	57.15	368.18

Figure 2 – Supplement File 1
Peptides detected through mass spectrometry
Strain: GRO.DD
Plasmid: [UAG-GFP]
Spectrum 19

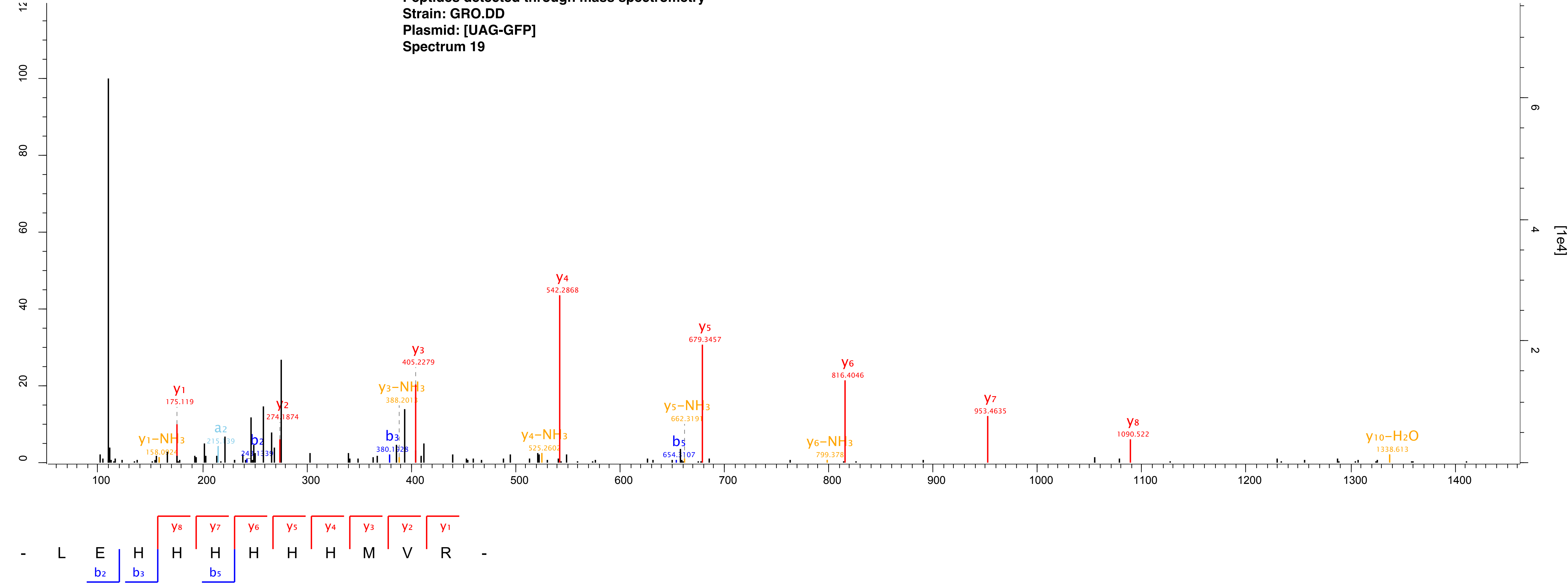

Raw file	Scan	Method	Score	m/z
VOT16-1542	1711	FTMS; HCD	47.3	490.57

Figure 2 – Supplement File 1
Peptides detected through mass spectrometry
Strain: GRO.DD
Plasmid: [UAG-GFP]
Spectrum 20

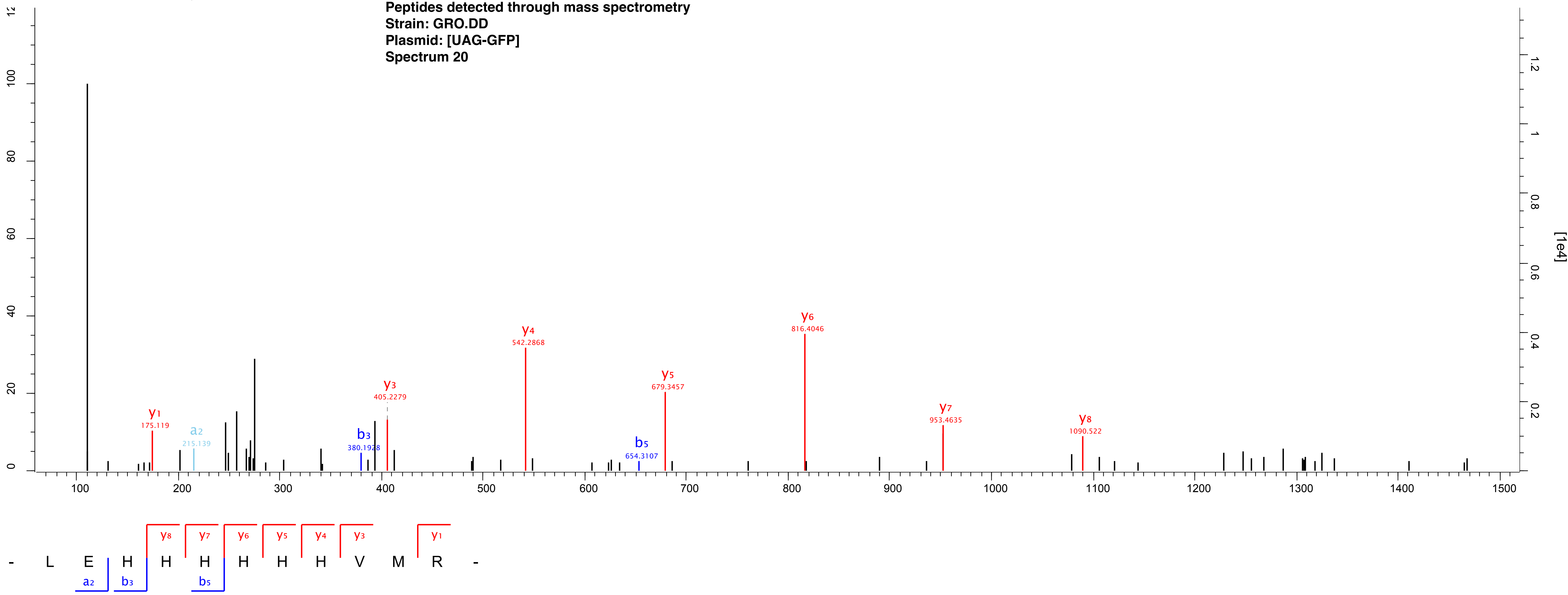

Raw file	Scan	Method	Score	m/z
VOT16-1542	1726	FTMS; HCD	14.01	485.24

Figure 2 – Supplement File 1
Peptides detected through mass spectrometry
Strain: GRO.DD
Plasmid: [UAG-GFP]
Spectrum 21

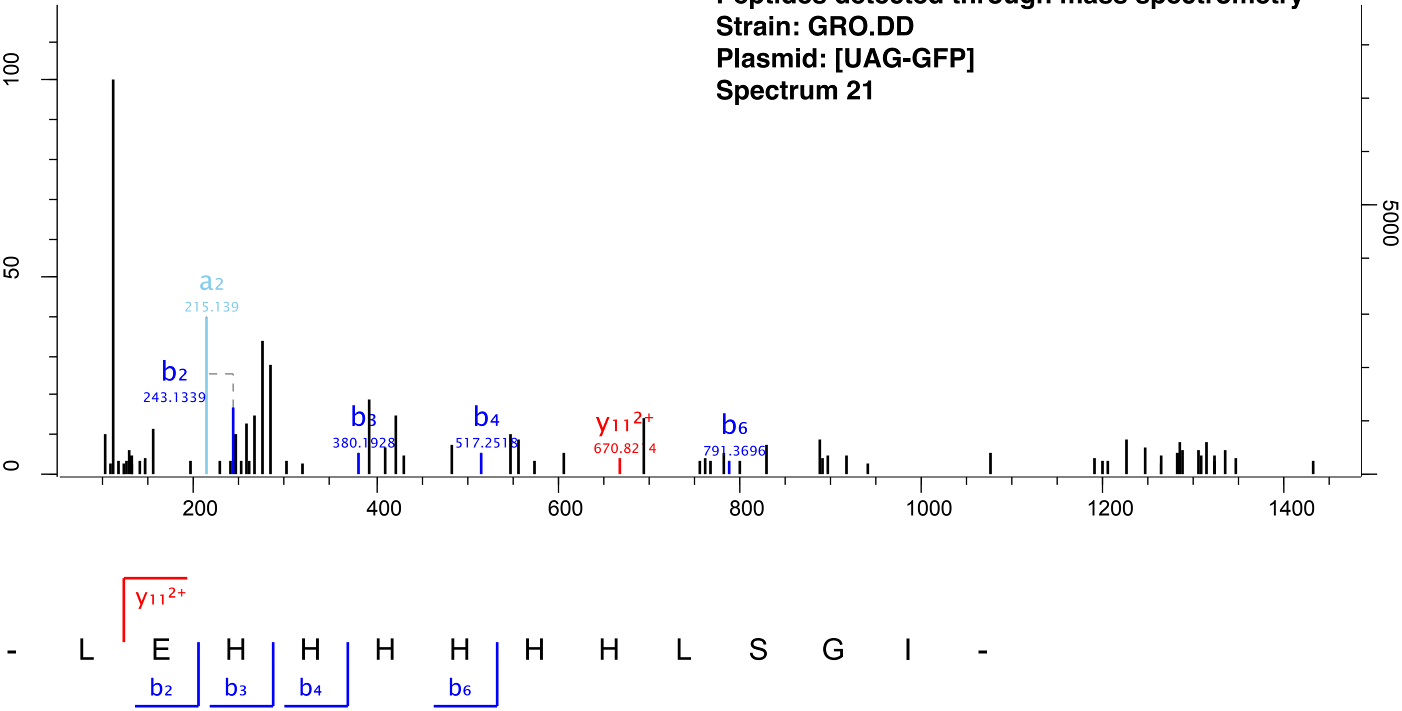

Raw file	Scan	Method	Score	m/z
VOT16-1542	1751	FTMS; HCD	56.4	360.67

Figure 2 – Supplement File 1
Peptides detected through mass spectrometry
Strain: GRO.DD
Plasmid: [UAG-GFP]
Spectrum 22

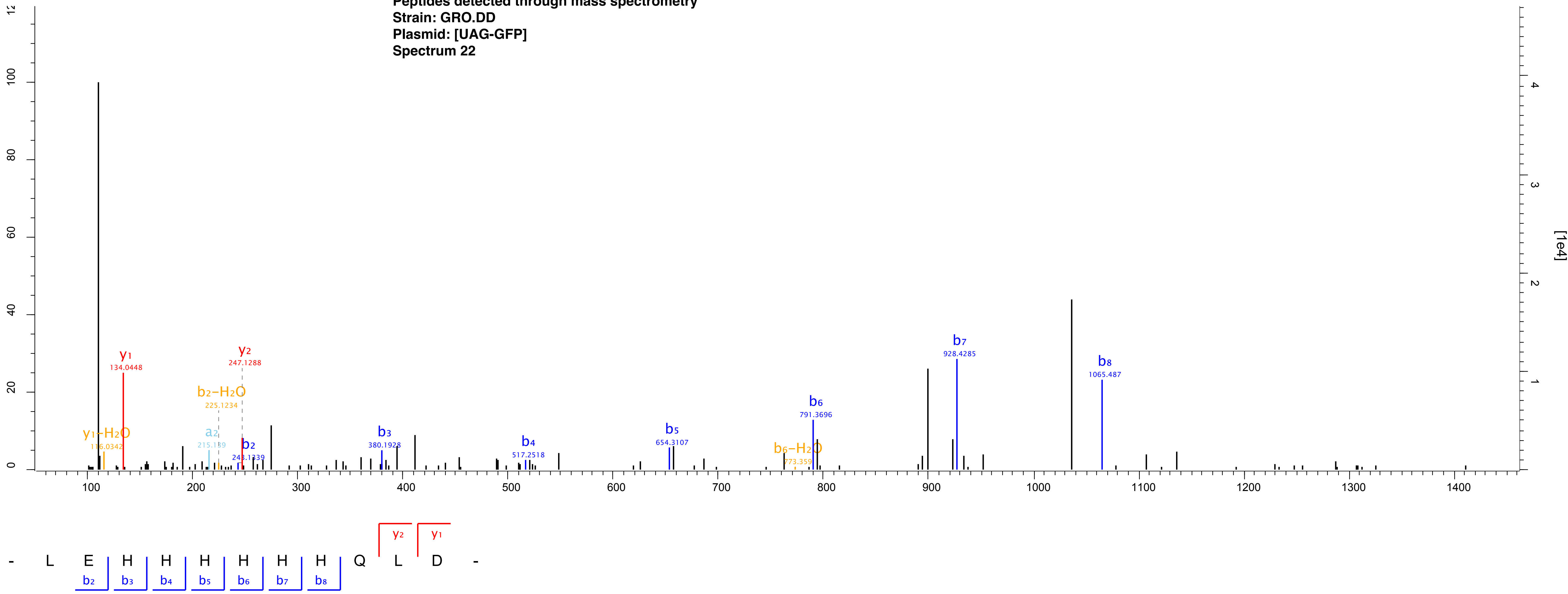

Raw file	Scan	Method	Score	m/z
VOT16-1542	1766	FTMS; HCD	46.41	383.44

Figure 2 – Supplement File 1
Peptides detected through mass spectrometry
Strain: GRO.DD
Plasmid: [UAG-GFP]
Spectrum 23

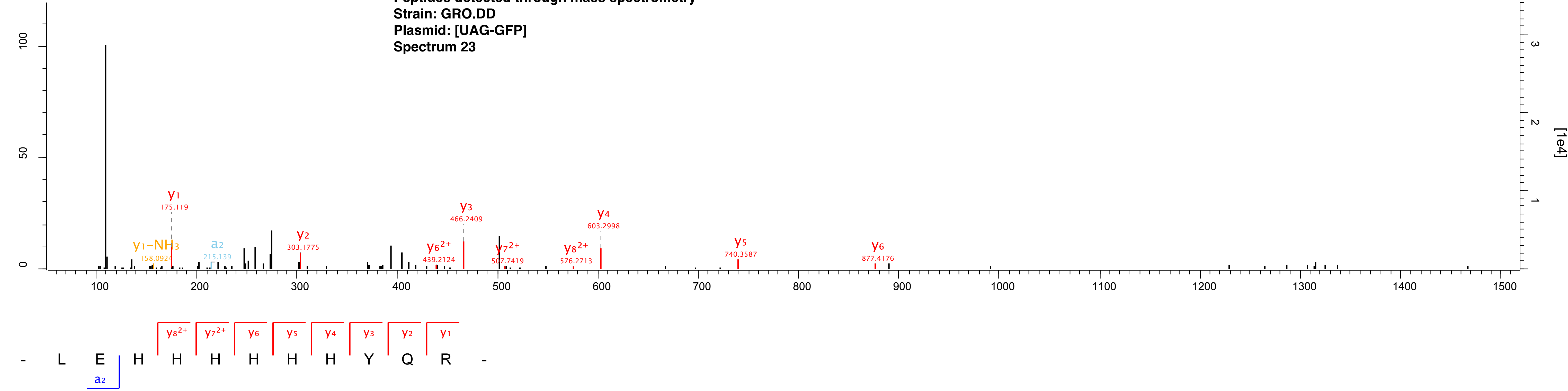

Raw file	Scan	Method	Score	m/z
VOT16-1571	1770	FTMS; HCD	72.36	638.53

Figure 2 – Supplement File 1
Peptides detected through mass spectrometry
Strain: GRO.DD
Plasmid: [UAG-GFP]
Spectrum 24

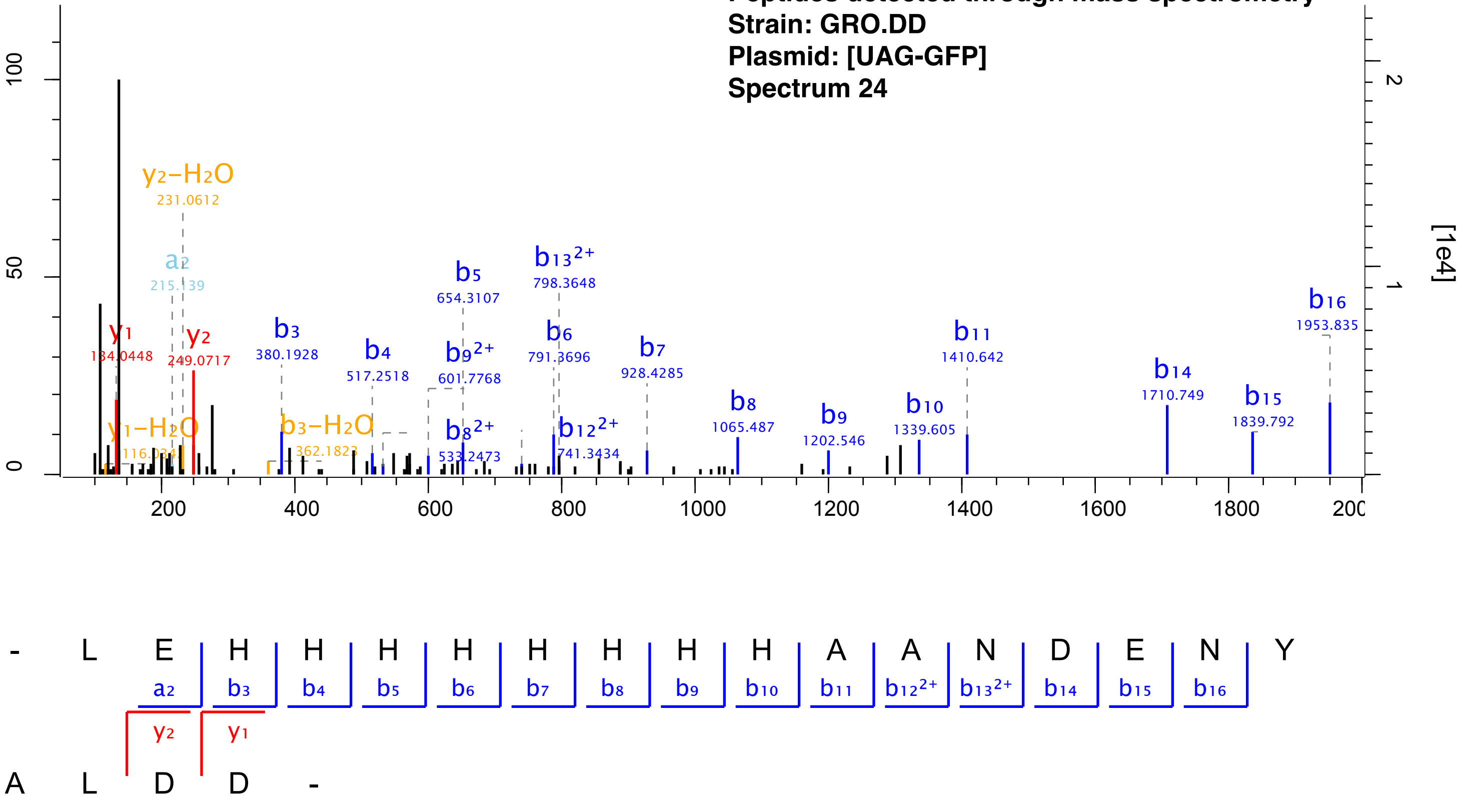

Raw file	Scan	Method	Score	m/z
VOT16-1571	1777	FTMS; HCD	97.77	618.3

Figure 2 – Supplement File 1
Peptides detected through mass spectrometry
Strain: GRO.DD
Plasmid: [UAG-GFP]
Spectrum 25

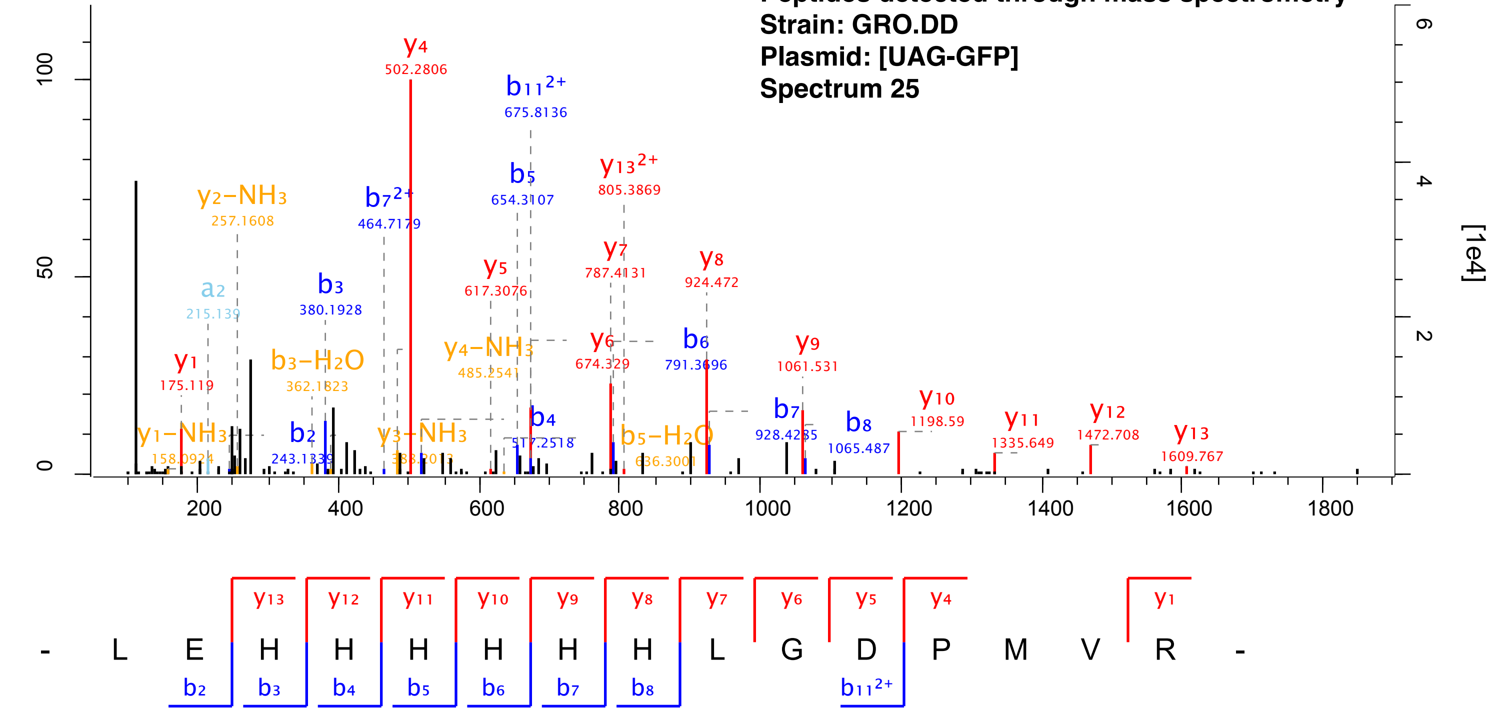

Raw file	Scan	Method	Score	m/z
VOT16-1542	1785	FTMS; HCD	159.12	580.28

Figure 2 – Supplement File 1
Peptides detected through mass spectrometry
Strain: GRO.DD
Plasmid: [UAG-GFP]
Spectrum 26

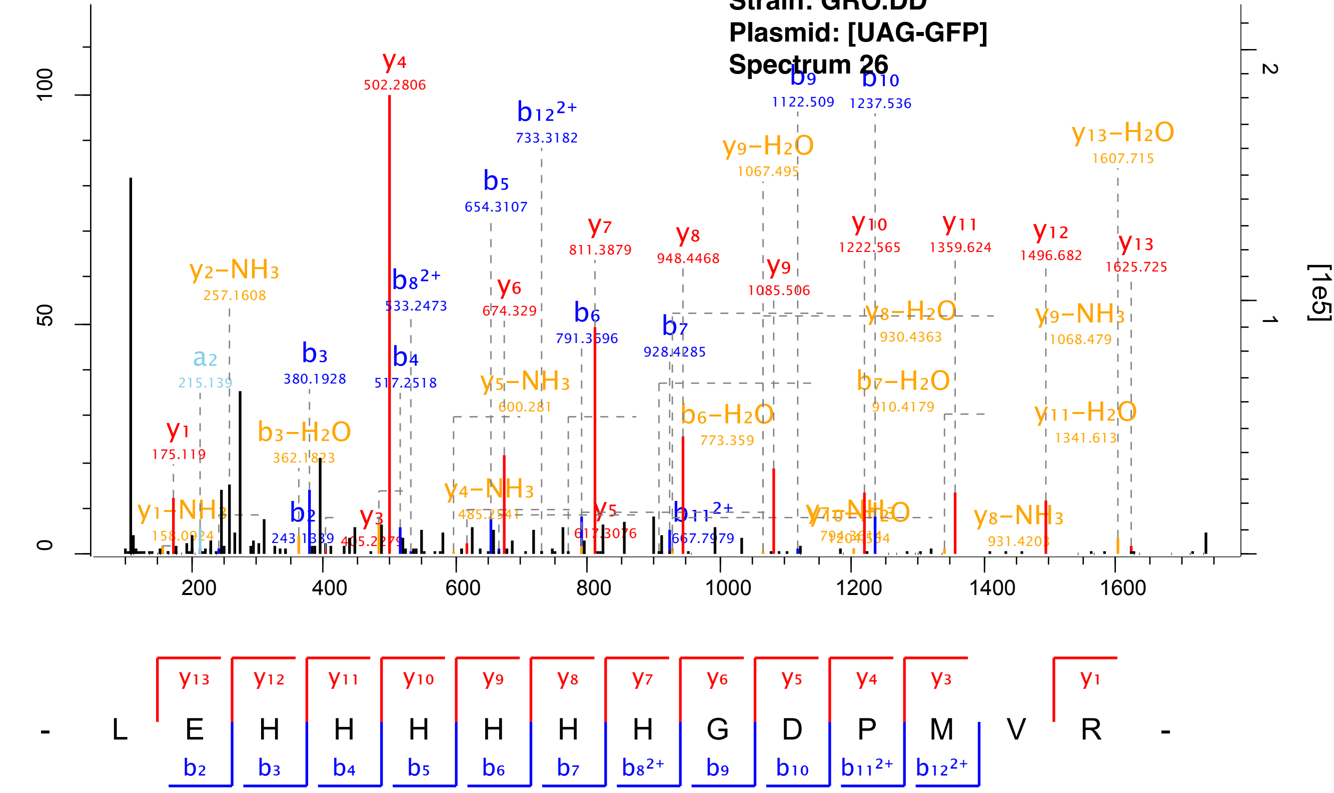

Raw file	Scan	Method	Score	m/z
VOT16-1542	1785	FTMS; HCD	159.12	580.28

Figure 2 – Supplement File 1
Peptides detected through mass spectrometry
Strain: GRO.DD
Plasmid: [UAG-GFP]
Spectrum 27

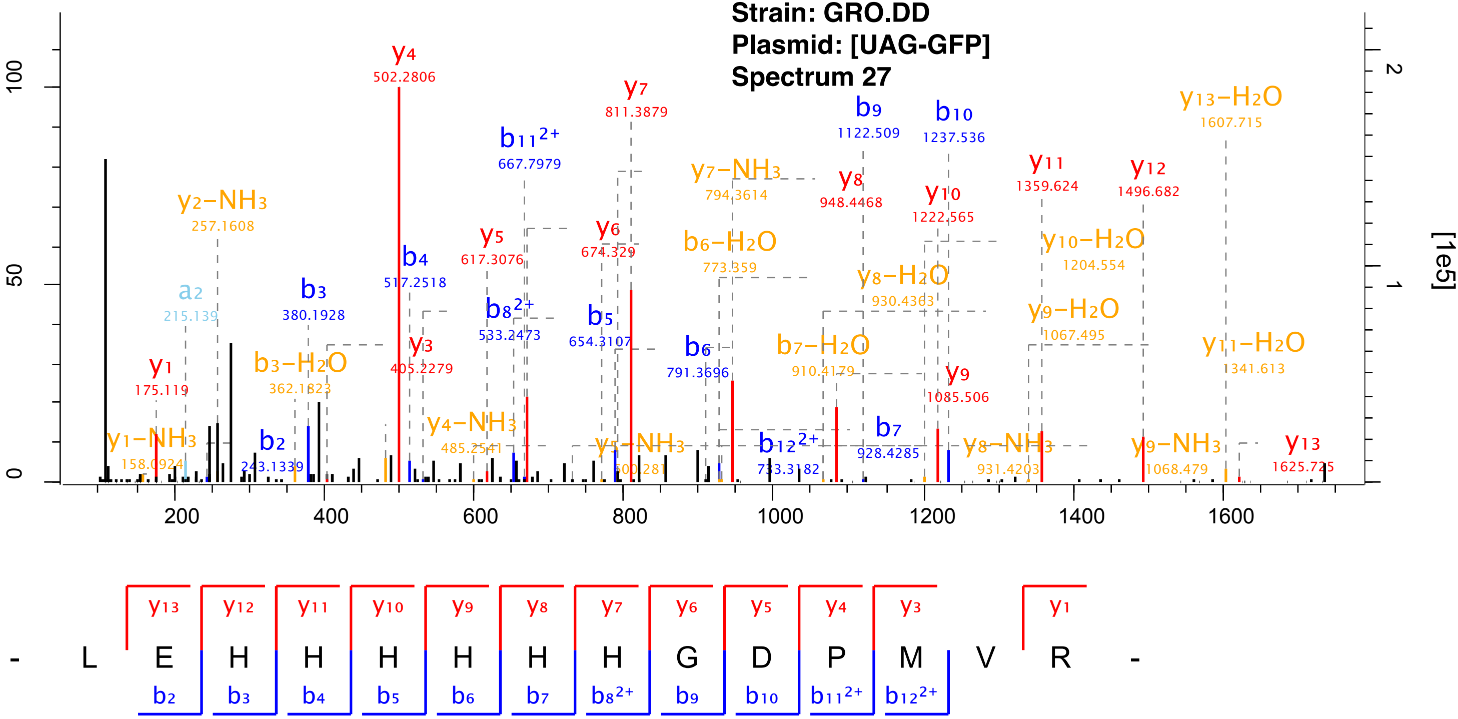

Raw file	Scan	Method	Score	m/z
VOT16-1571	1786	FTMS; HCD	99.04	805.01

Figure 2 – Supplement File 1
Peptides detected through mass spectrometry
Strain: GRO.DD
Plasmid: [UAG-GFP]
Spectrum 28

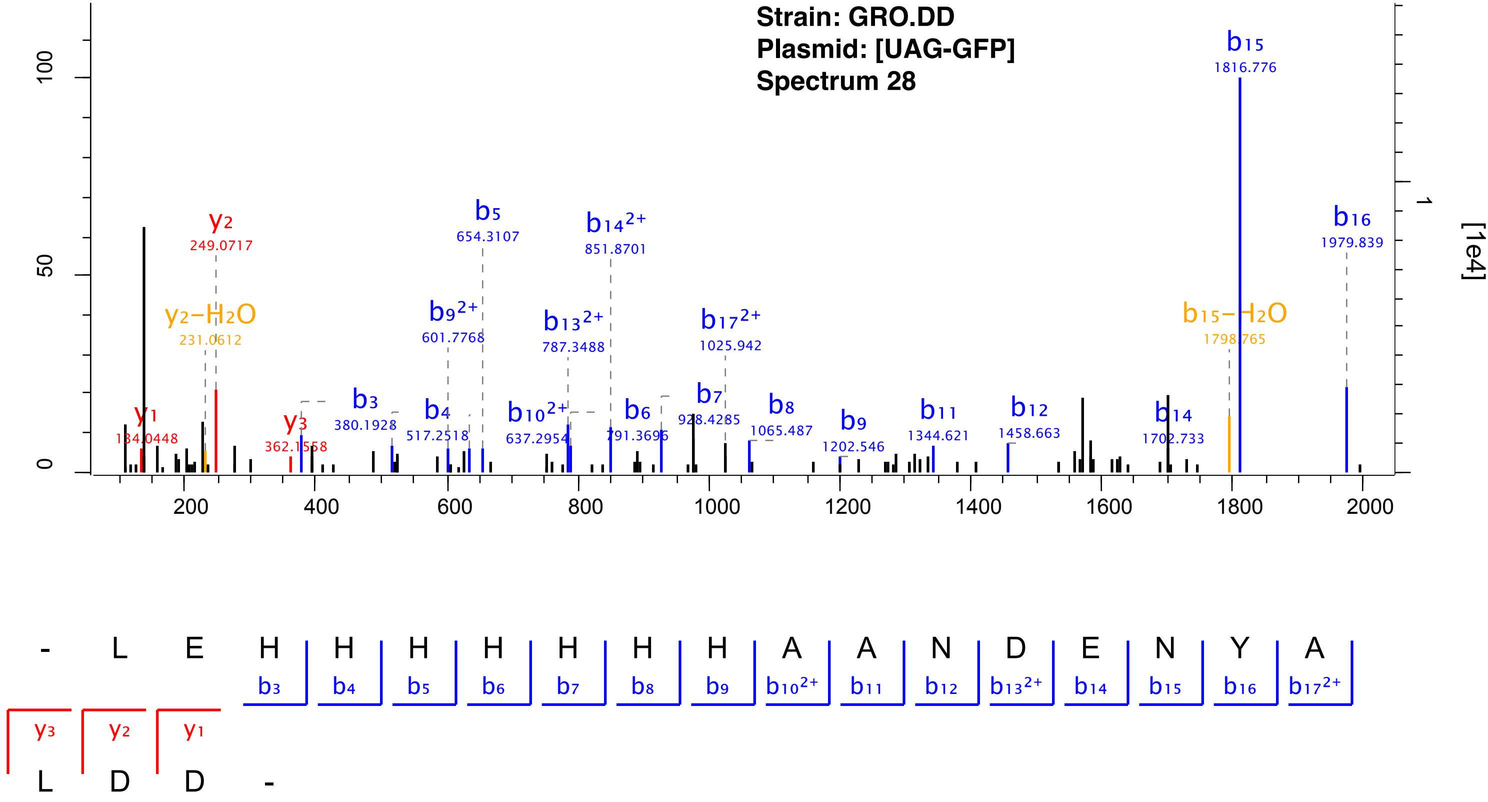

Raw file	Scan	Method	Score	m/z
VOT16-1571	1832	FTMS; HCD	31.41	582.97

Figure 2 – Supplement File 1
Peptides detected through mass spectrometry
Strain: GRO.DD
Plasmid: [UAG-GFP]
Spectrum 29

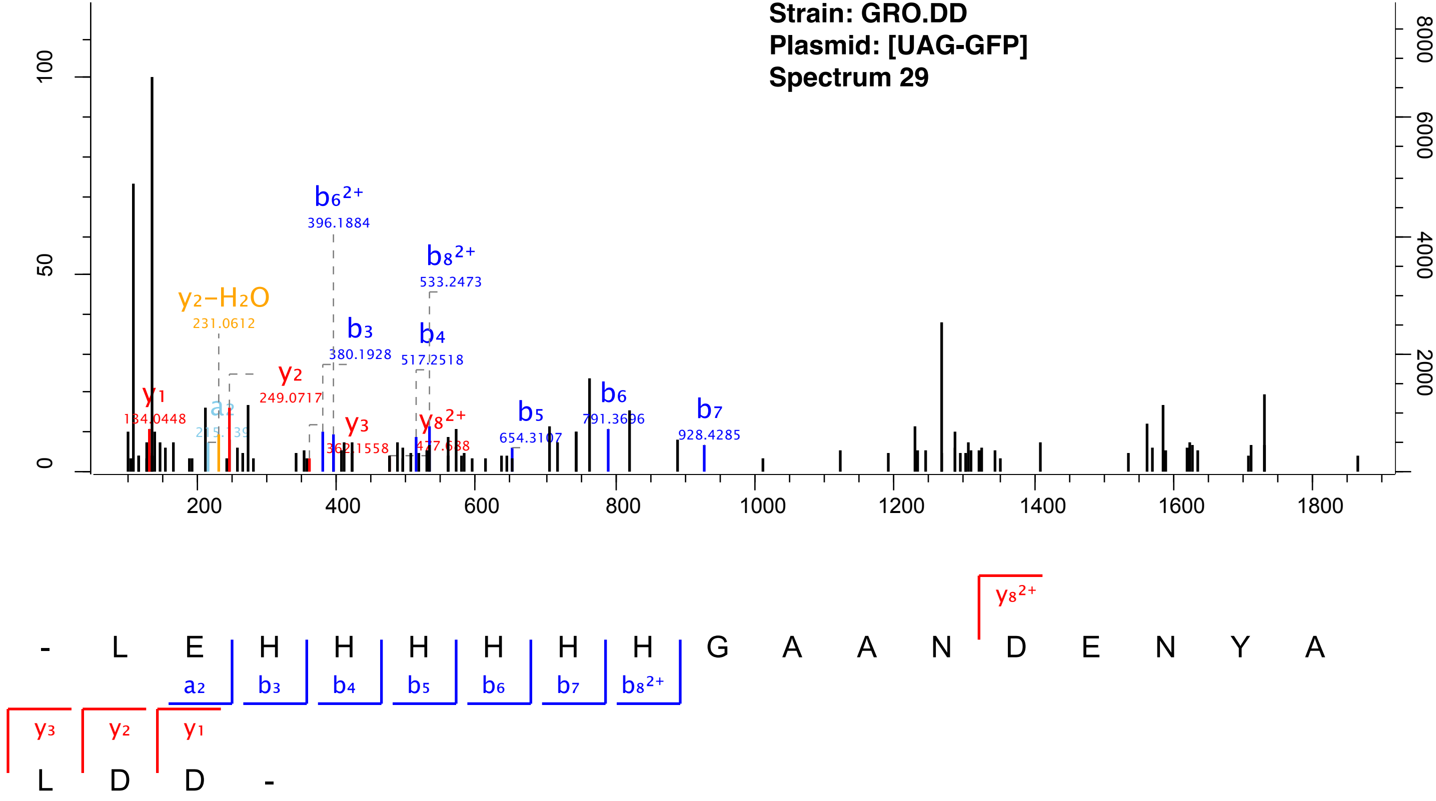

Raw file	Scan	Method	Score	m/z
VOT16-1571	1843	FTMS; HCD	146.78	1138.48

Figure 2 – Supplement File 1
Peptides detected through mass spectrometry
Strain: GRO.DD
Plasmid: [UAG-GFP]
Spectrum 30

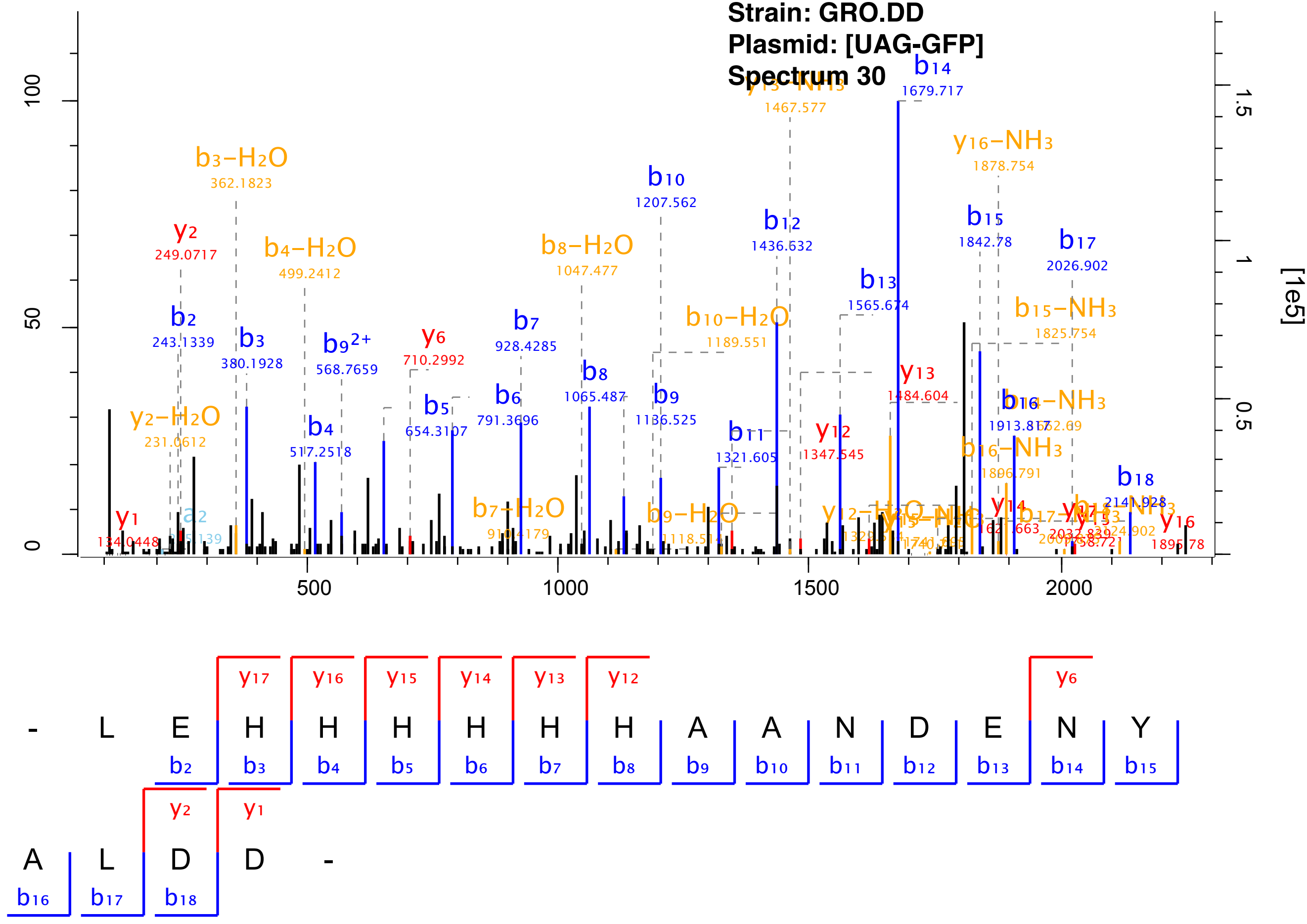

Raw file	Scan	Method	Score	m/z
VOT16-1571	1843	FTMS; HCD	146.78	1138.48

Figure 2 – Supplement File 1
Peptides detected through mass spectrometry
Strain: GRO.DD
Plasmid: [UAG-GFP]
Spectrum 31

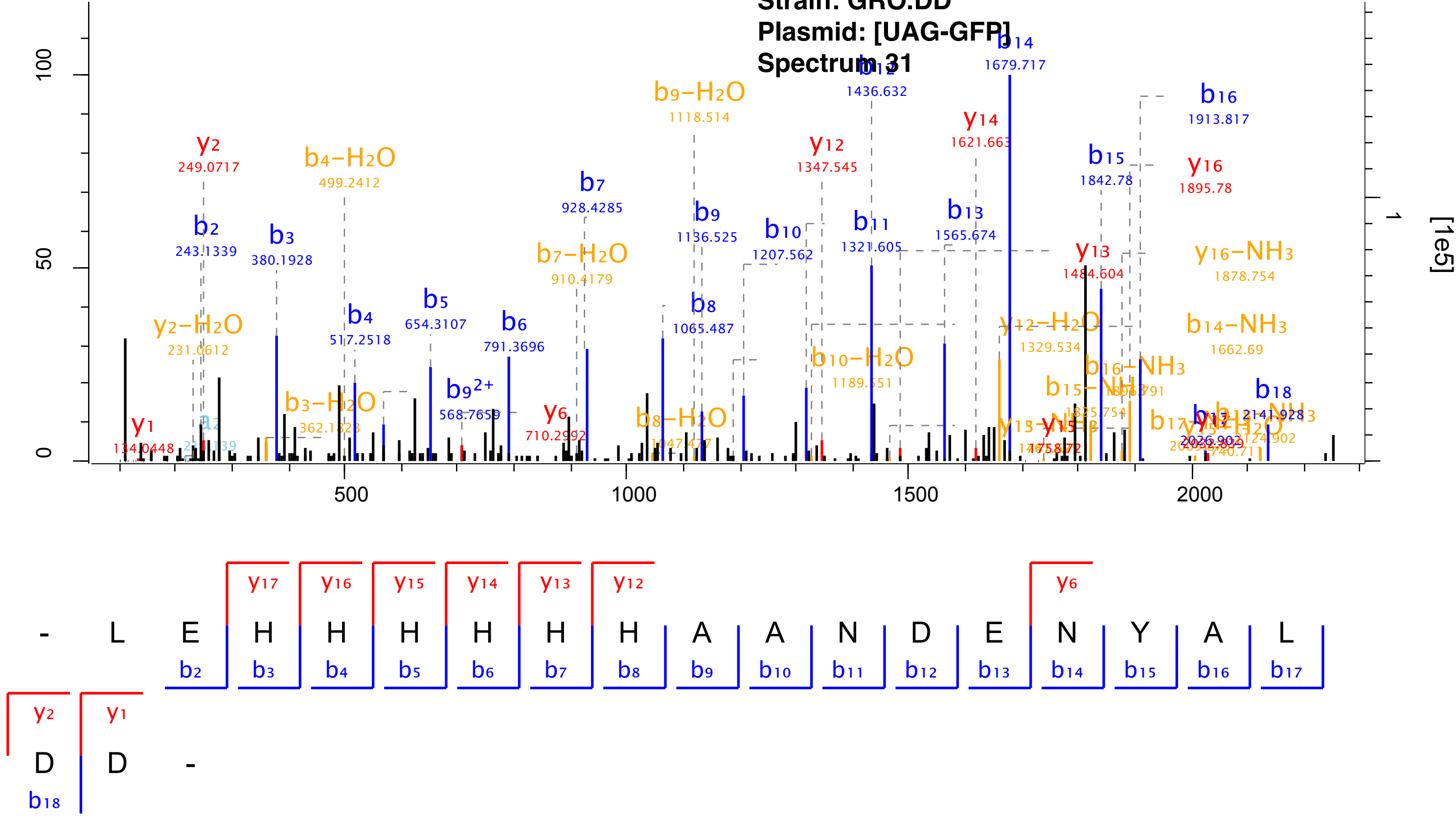

Raw file	Scan	Method	Score	m/z
VOT16-1542	1846	FTMS; HCD	15.94	503.58

Figure 2 – Supplement File 1
Peptides detected through mass spectrometry
Strain: GRO.DD
Plasmid: [UAG-GFP]
Spectrum 32

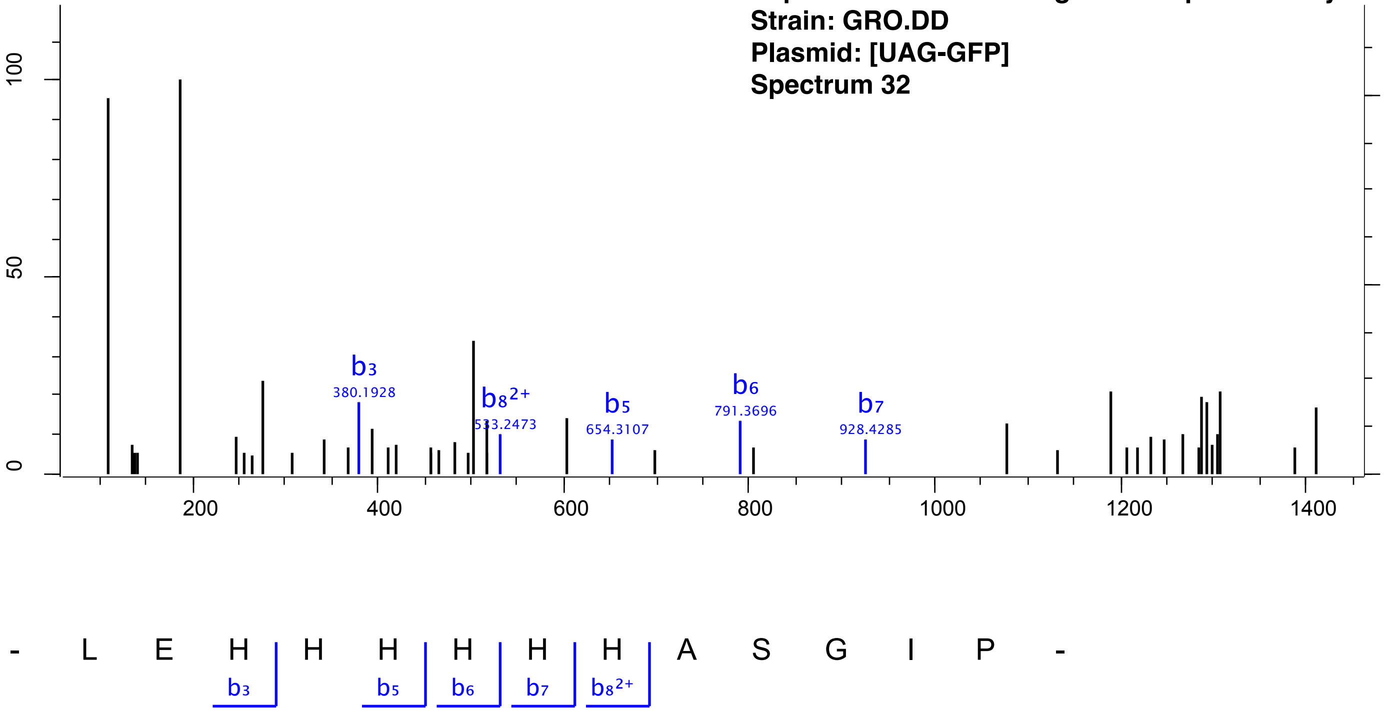

Raw file	Scan	Method	Score	m/z
VOT16-1542	1856	FTMS; HCD	41.08	479.9

Figure 2 – Supplement File 1
Peptides detected through mass spectrometry
Strain: GRO.DD
Plasmid: [UAG-GFP]
Spectrum 33

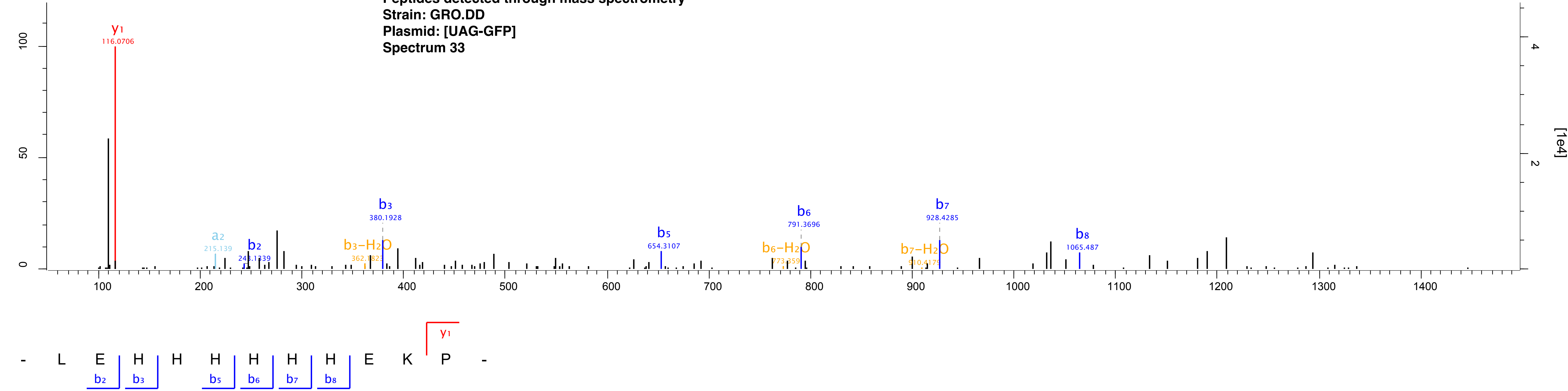

Raw file	Scan	Method	Score	m/z
VOT16-1571	1857	FTMS; HCD	79.84	513.5

Figure 2 – Supplement File 1
Peptides detected through mass spectrometry
Strain: GRO.DD
Plasmid: [UAG-GFP]
Spectrum 34

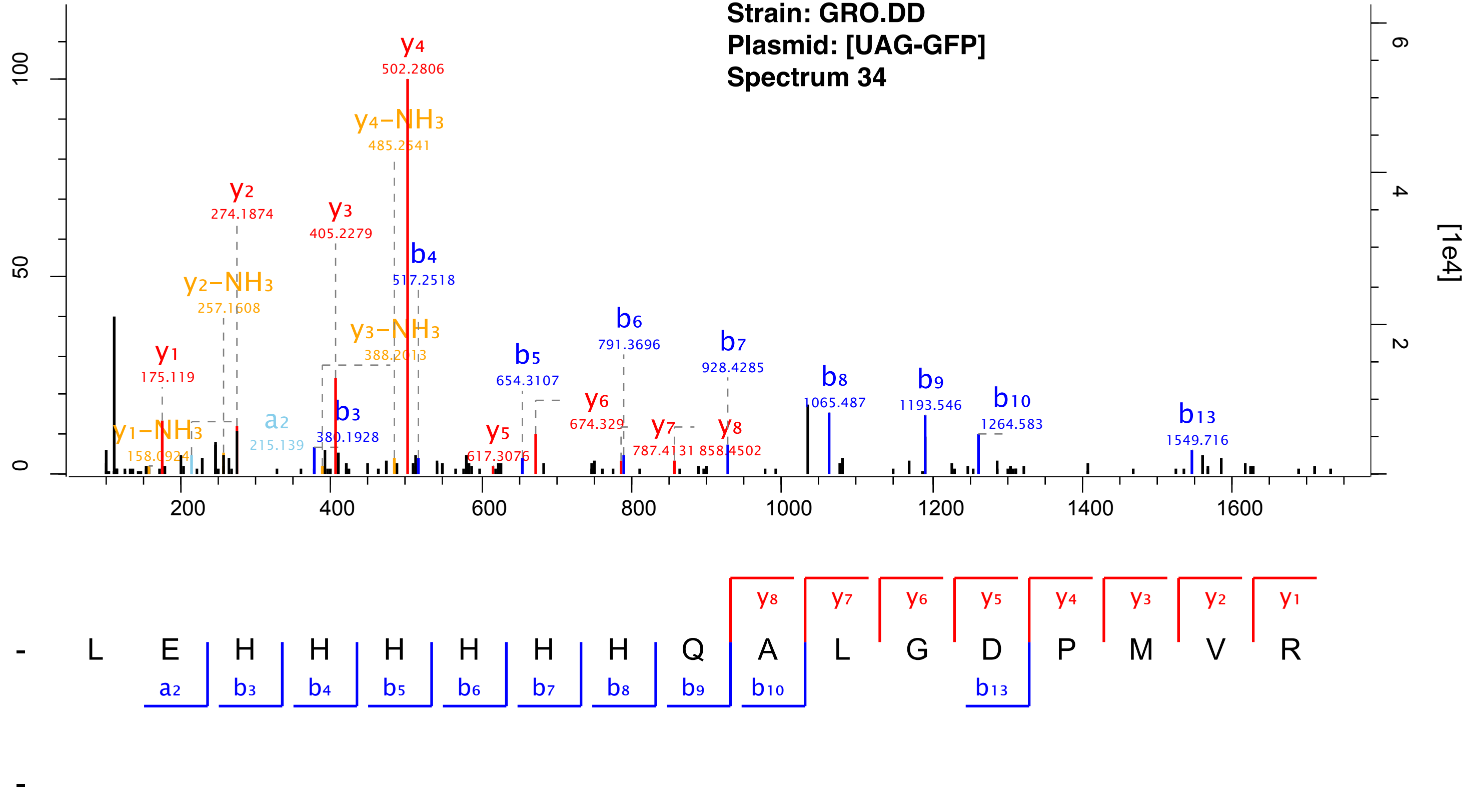

Raw file	Scan	Method	Score	m/z
VOT16-1571	1888	FTMS; HCD	18.58	494.97

Figure 2 – Supplement File 1
Peptides detected through mass spectrometry
Strain: GRO.DD
Plasmid: [UAG-GFP]
Spectrum 35

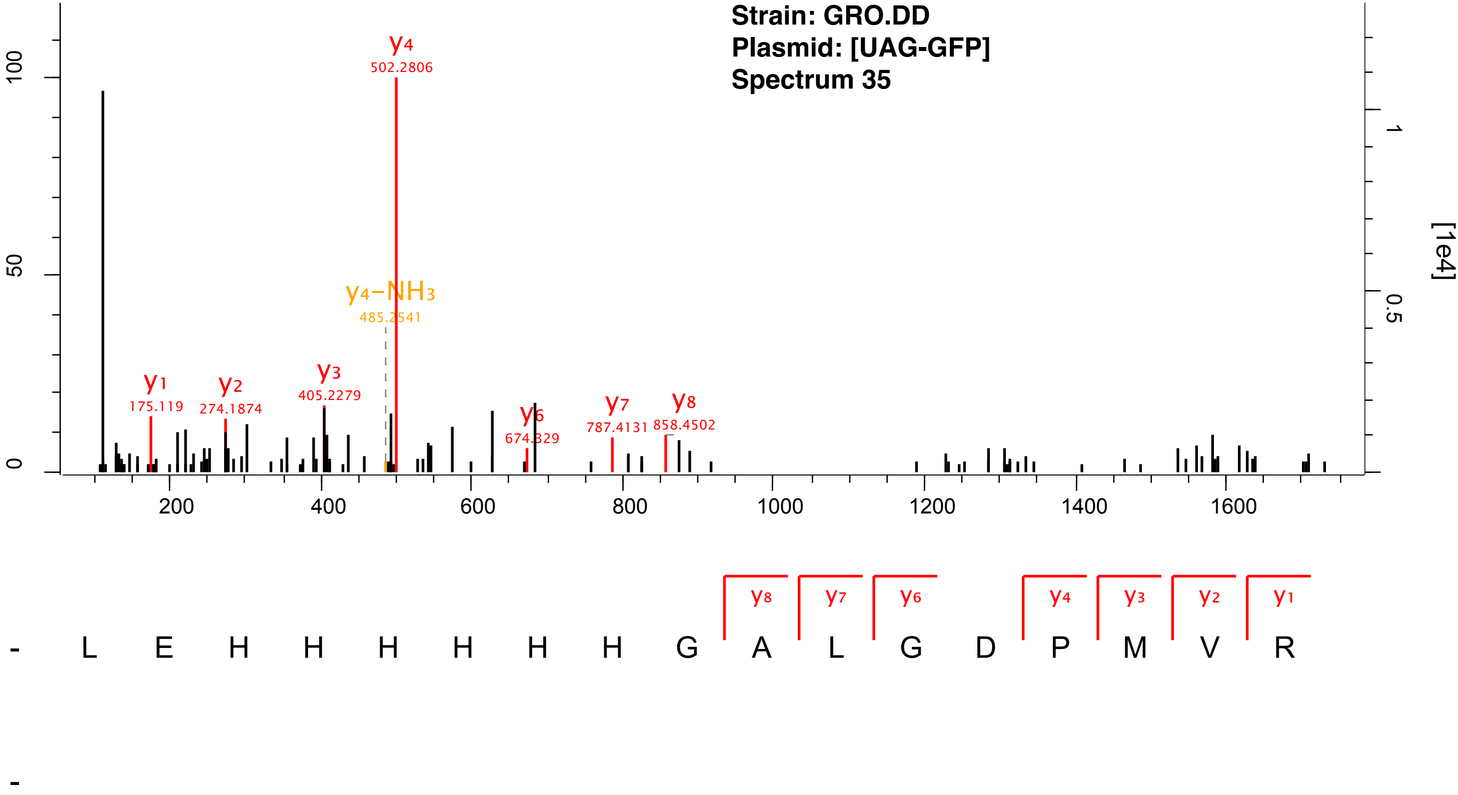

Raw file	Scan	Method	Score	m/z
VOT16-1542	1911	FTMS; HCD	91.31	528.24

Figure 2 – Supplement File 1
Peptides detected through mass spectrometry
Strain: GRO.DD
Plasmid: [UAG-GFP]
Spectrum 36

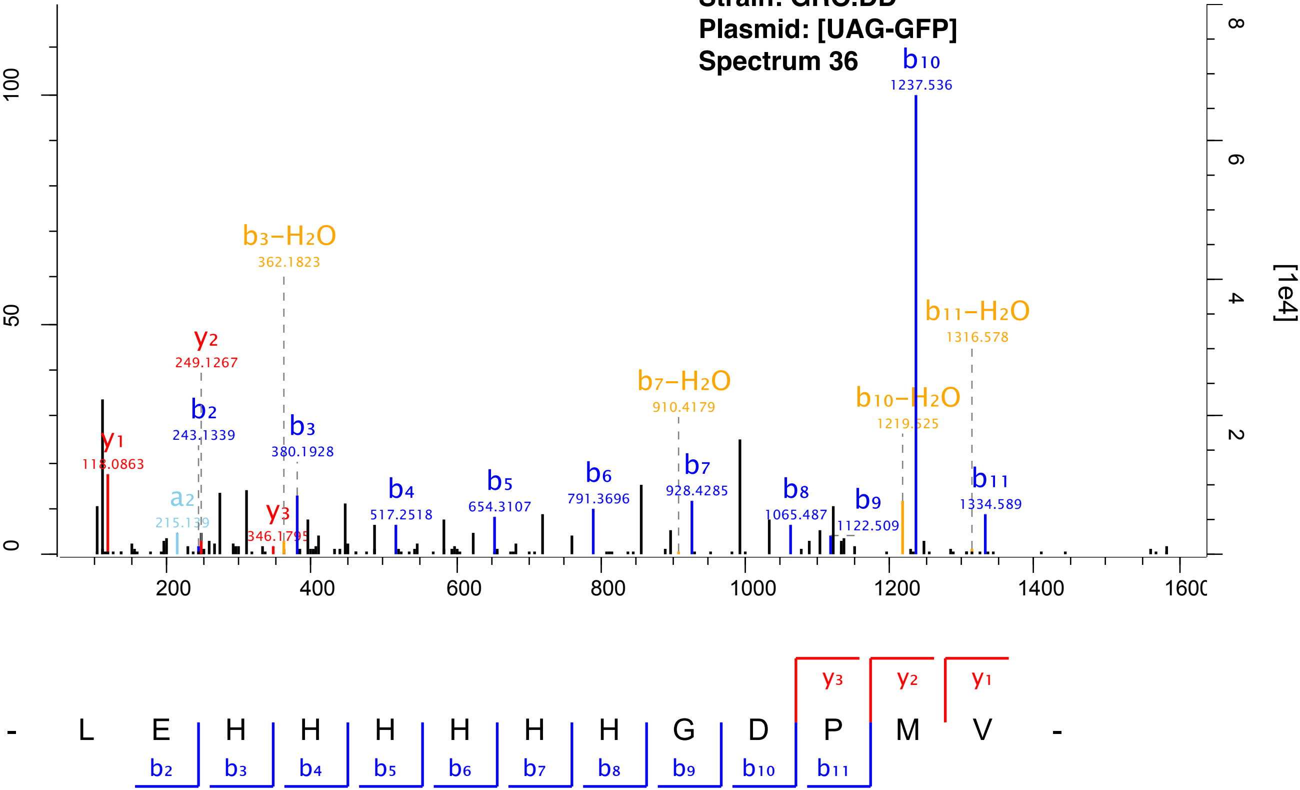

Raw file	Scan	Method	Score	m/z
VOT16-1571	1955	FTMS; HCD	32.8	633.78

Figure 2 – Supplement File 1
Peptides detected through mass spectrometry
Strain: GRO.DD
Plasmid: [UAG-GFP]
Spectrum 37

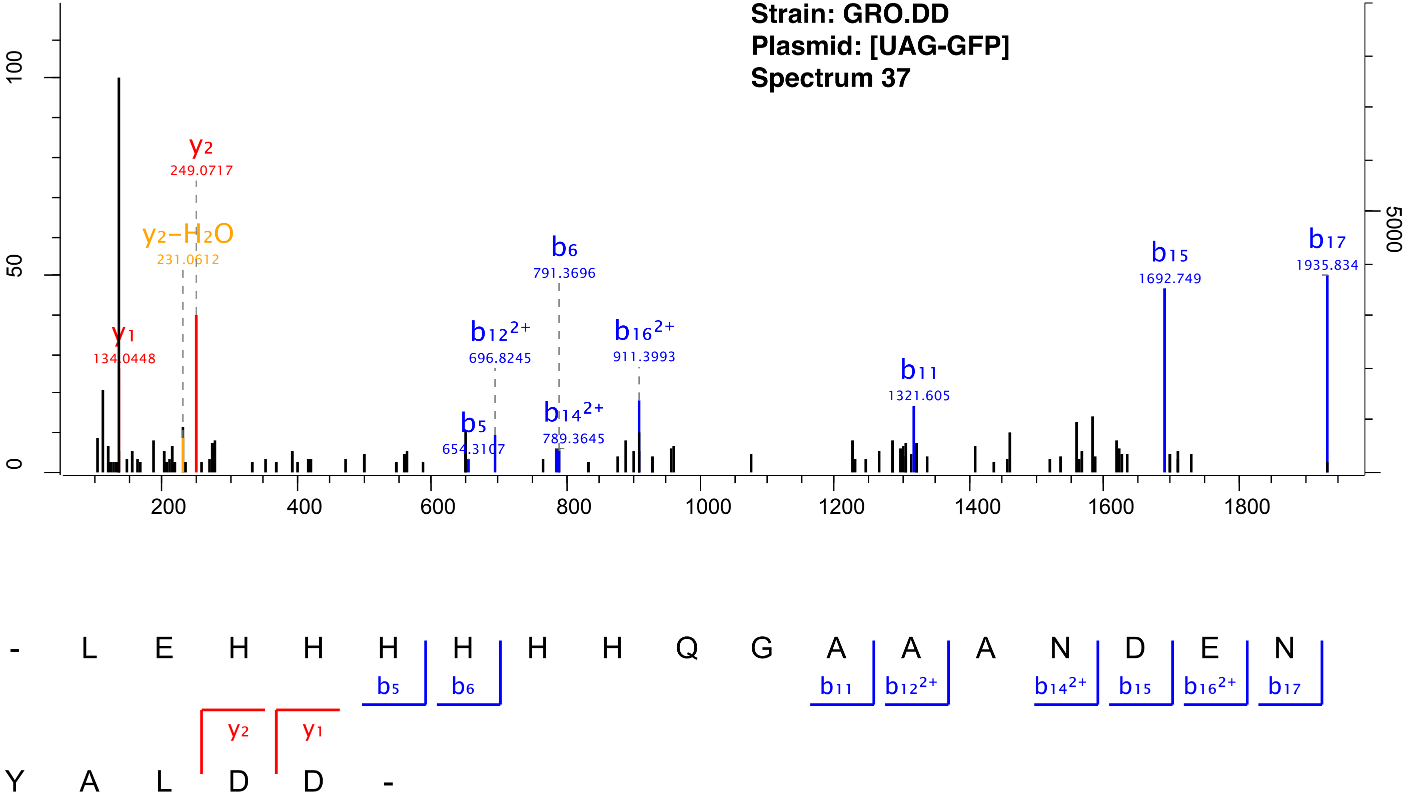

Raw file	Scan	Method	Score	m/z
VOT16-1571	1968	FTMS; HCD	65.42	806.69

Figure 2 – Supplement File 1
Peptides detected through mass spectrometry
Strain: GRO.DD
Plasmid: [UAG-GFP]
Spectrum 38

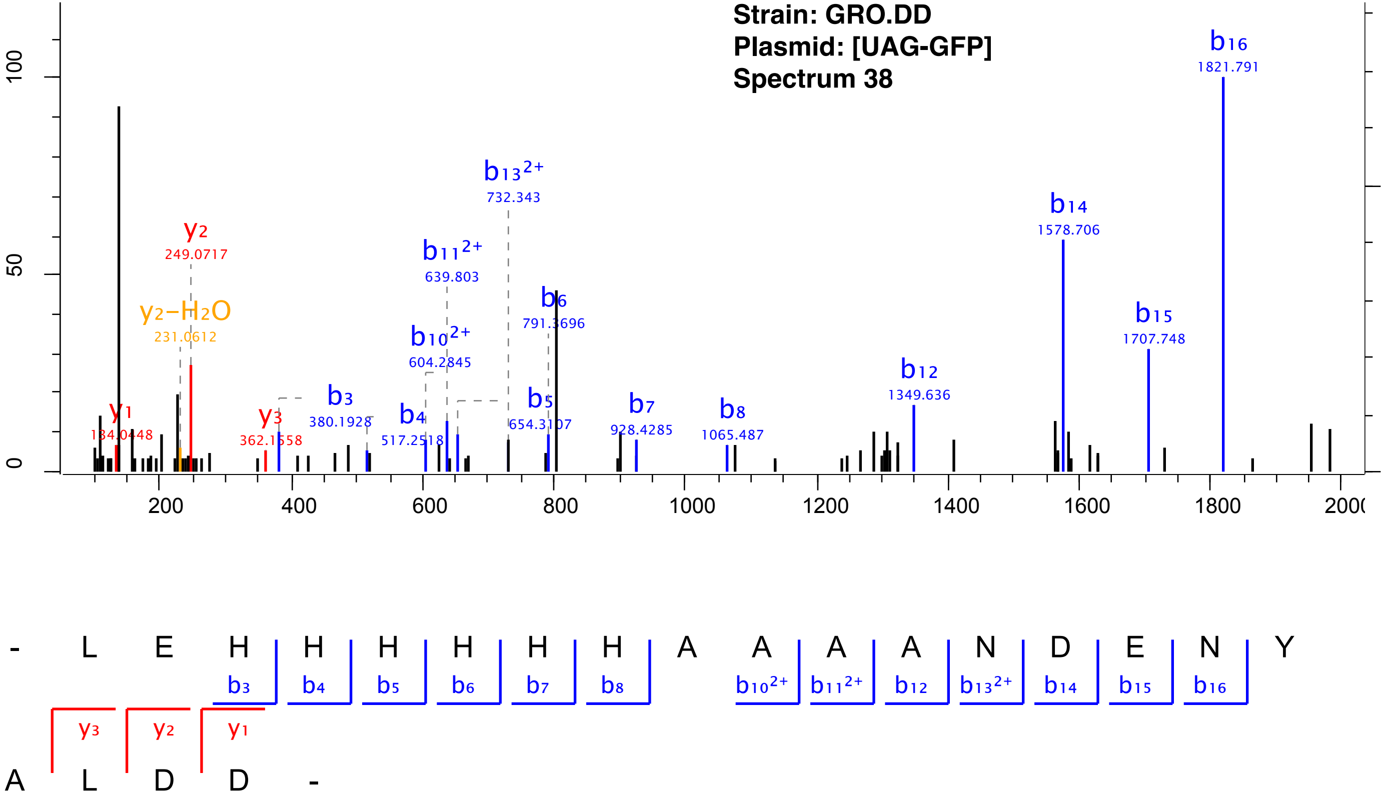

Raw file	Scan	Method	Score	m/z
VOT16-1571	2125	FTMS; HCD	93.24	481.49

Figure 2 – Supplement File 1
Peptides detected through mass spectrometry
Strain: GRO.DD
Plasmid: [UAG-GFP]
Spectrum 39

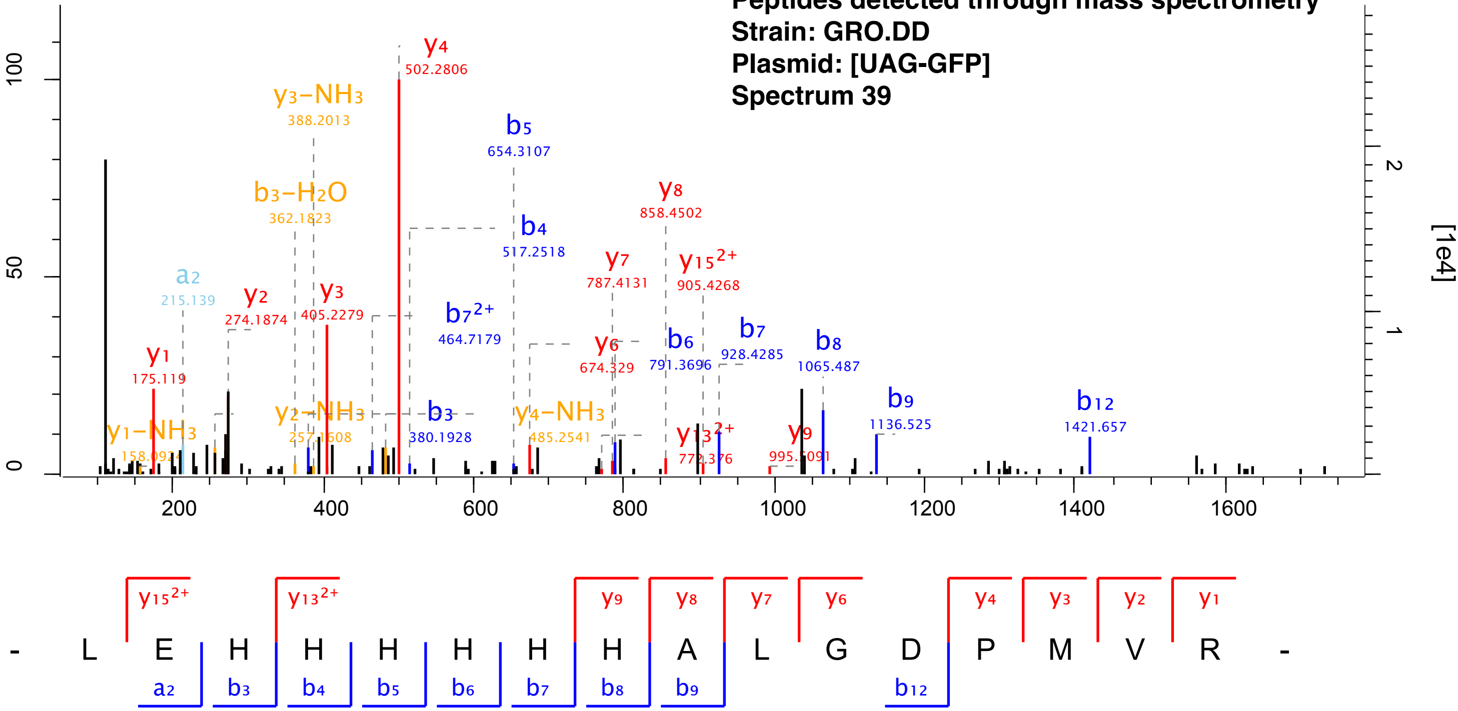

Raw file	Scan	Method	Score	m/z
VOT16-1542	2156	FTMS; HCD	73.14	605.76

Figure 2 – Supplement File 1
Peptides detected through mass spectrometry
Strain: GRO.DD
Plasmid: [UAG-GFP]
Spectrum 40

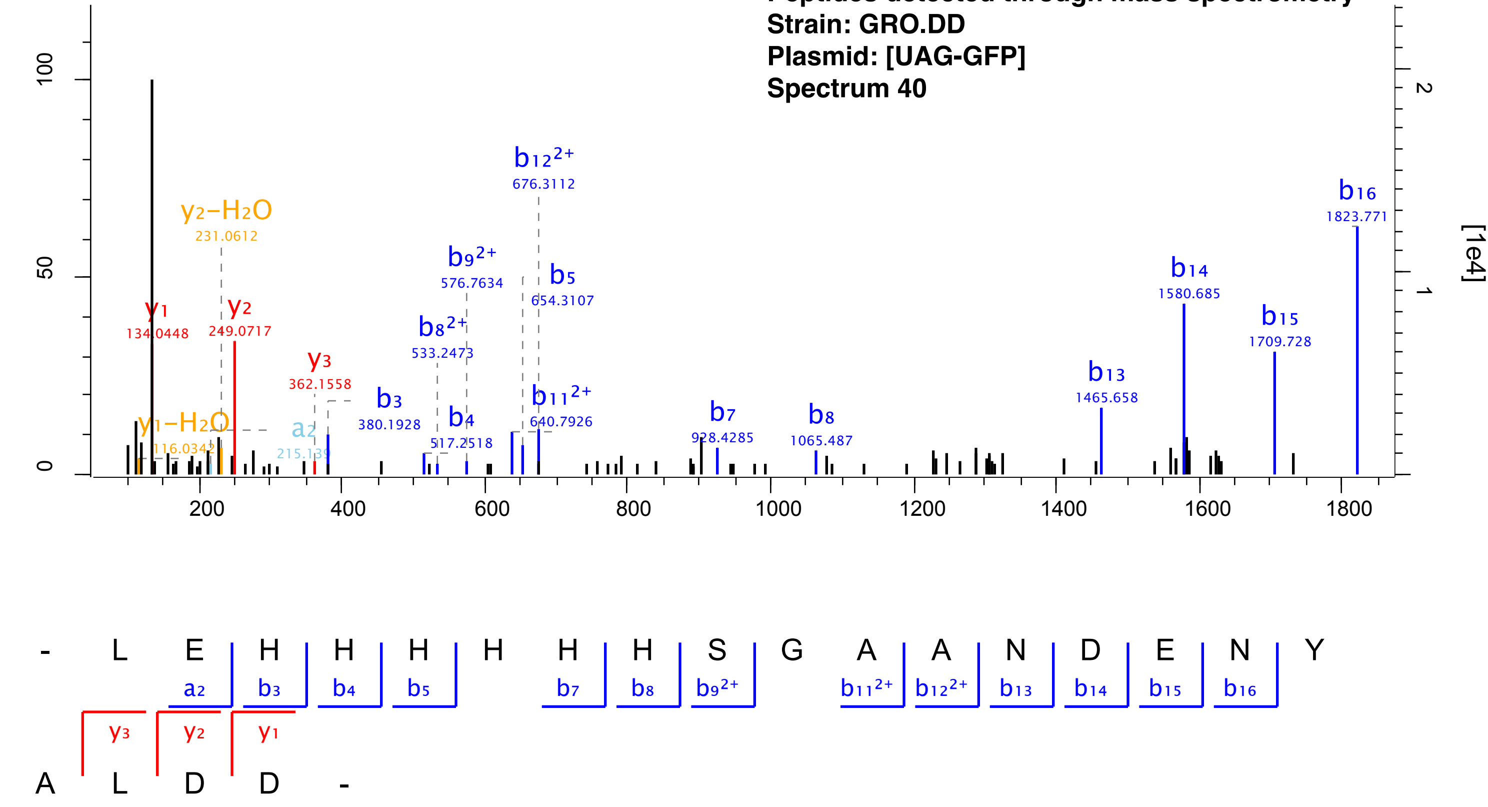

Raw file	Scan	Method	Score	m/z
VOT16-1542	2210	FTMS; HCD	63.3	612.51

Figure 2 – Supplement File 1
Peptides detected through mass spectrometry
Strain: GRO.DD
Plasmid: [UAG-GFP]
Spectrum 41

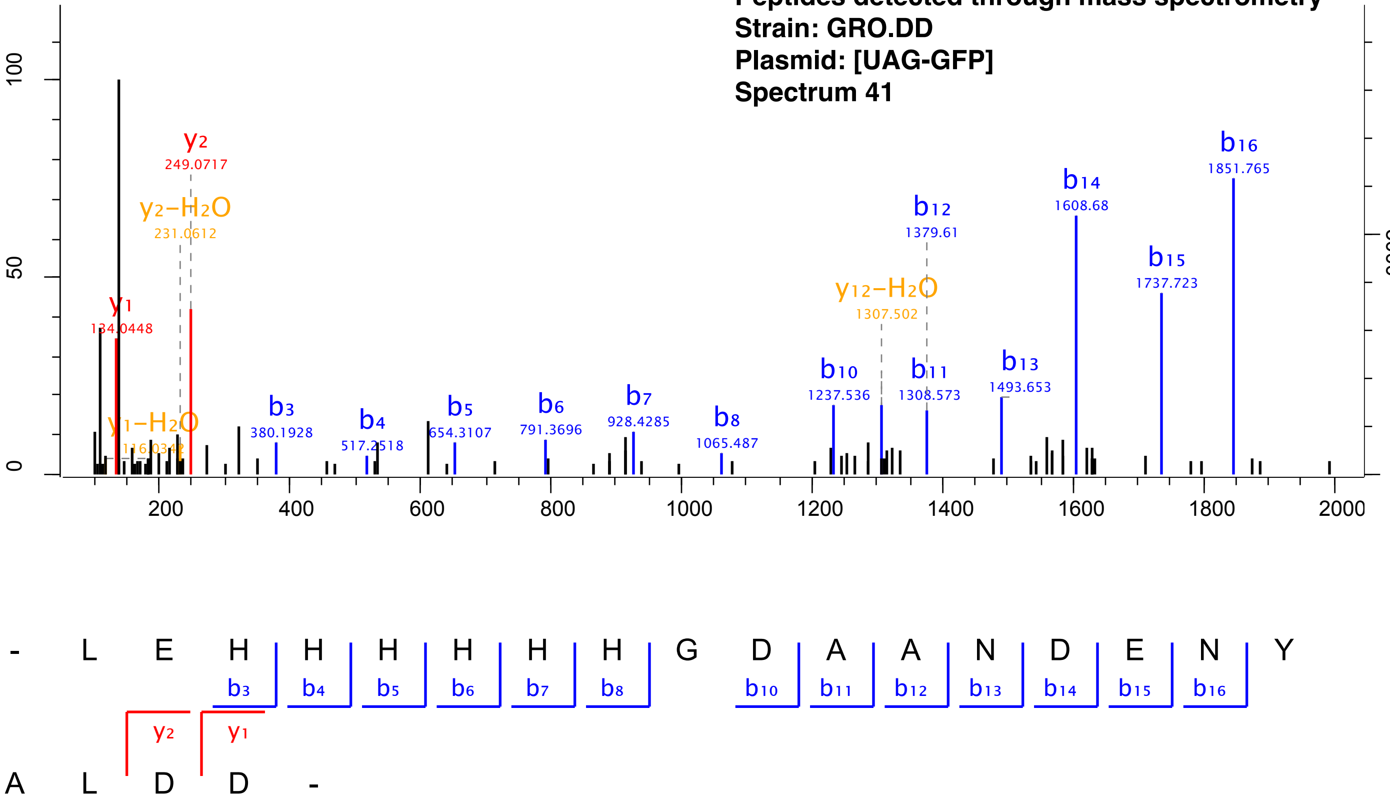

Raw file	Scan	Method	Score	m/z
VOT16-1542	2228	FTMS; HCD	95.82	481.74

Figure 2 – Supplement File 1
Peptides detected through mass spectrometry
Strain: GRO.DD
Plasmid: [UAG-GFP]
Spectrum 42

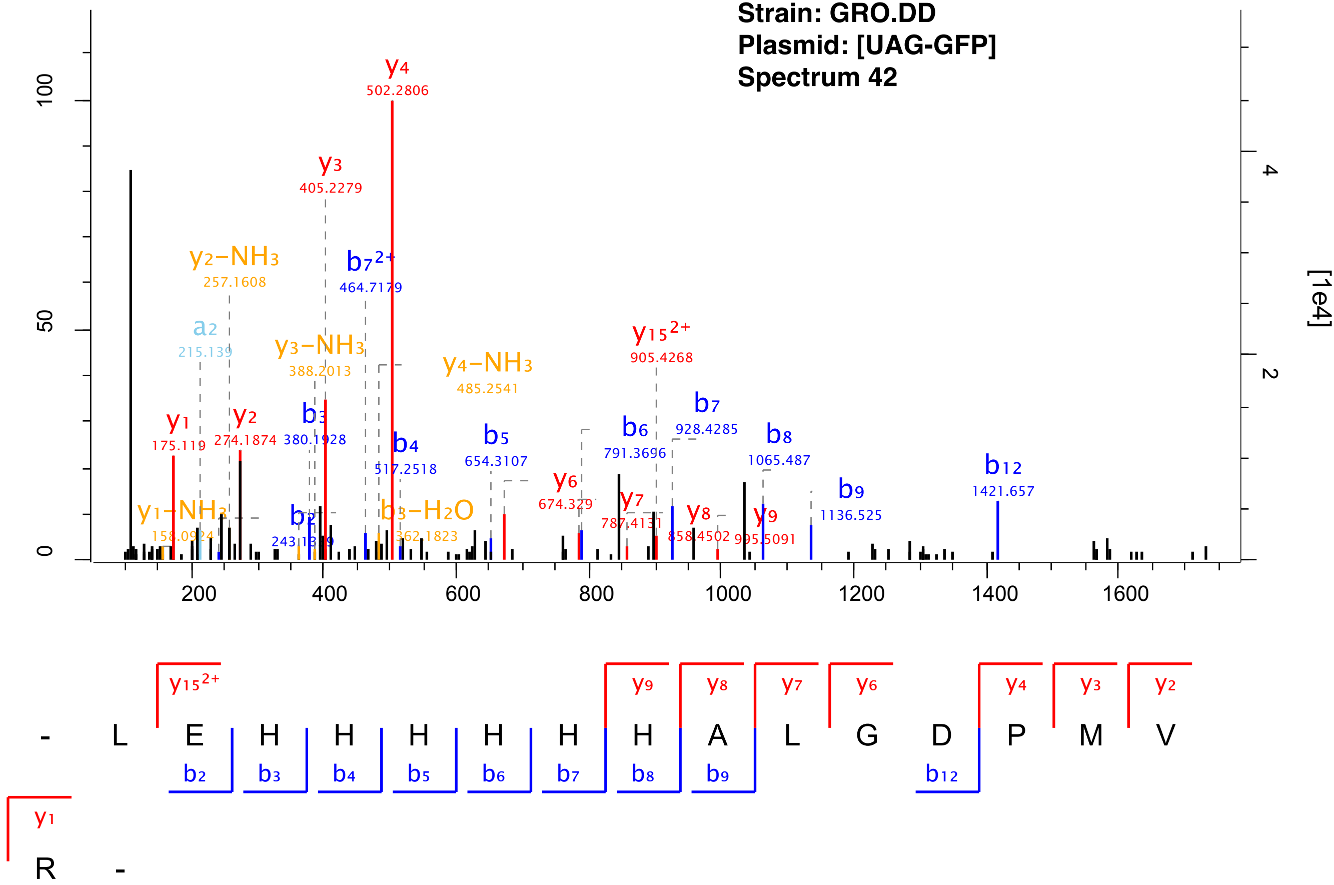

Raw file	Scan	Method	Score	m/z
VOT16-1542	2228	FTMS; HCD	95.82	481.74

Figure 2 – Supplement File 1
Peptides detected through mass spectrometry
Strain: GRO.DD
Plasmid: [UAG-GFP]
Spectrum 43

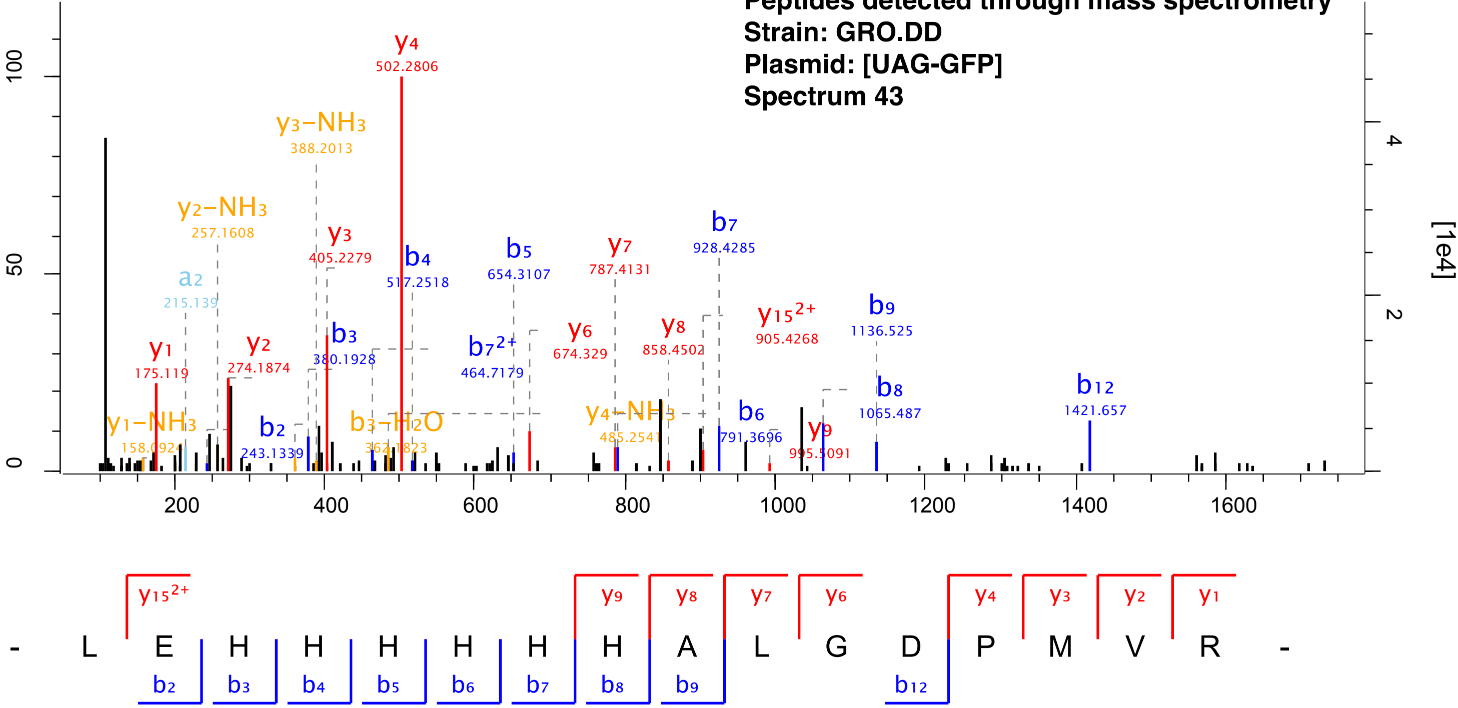

Raw file	Scan	Method	Score	m/z
VOT16-1571	3171	FTMS; HCD	120.32	674.28

Figure 2 – Supplement File 1
Peptides detected through mass spectrometry
Strain: GRO.DD
Plasmid: [UAG-GFP]
Spectrum 44

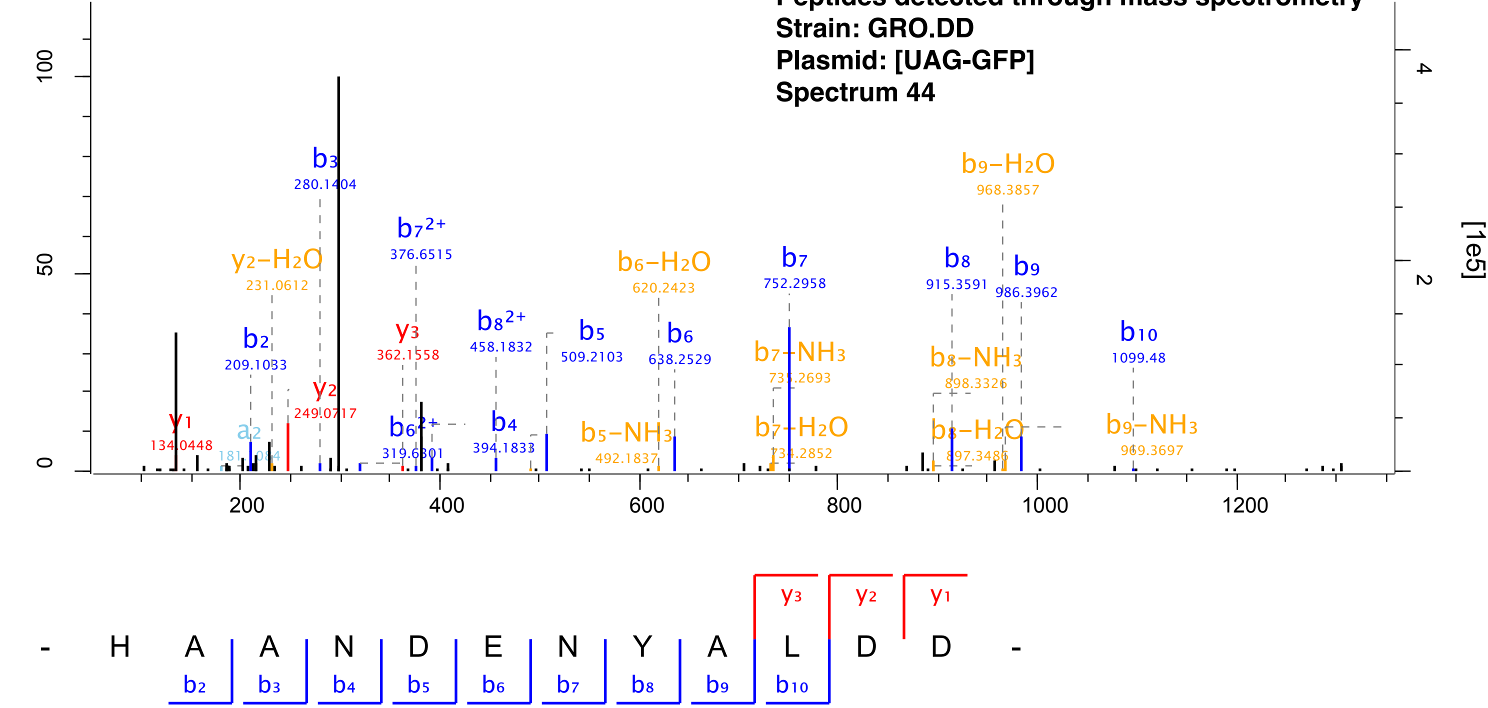

Raw file	Scan	Method	Score	m/z
VOT16-1542	3264	FTMS; HCD	9.57	658.54

Figure 2 – Supplement File 1
Peptides detected through mass spectrometry
Strain: GRO.DD
Plasmid: [UAG-GFP]
Spectrum 45

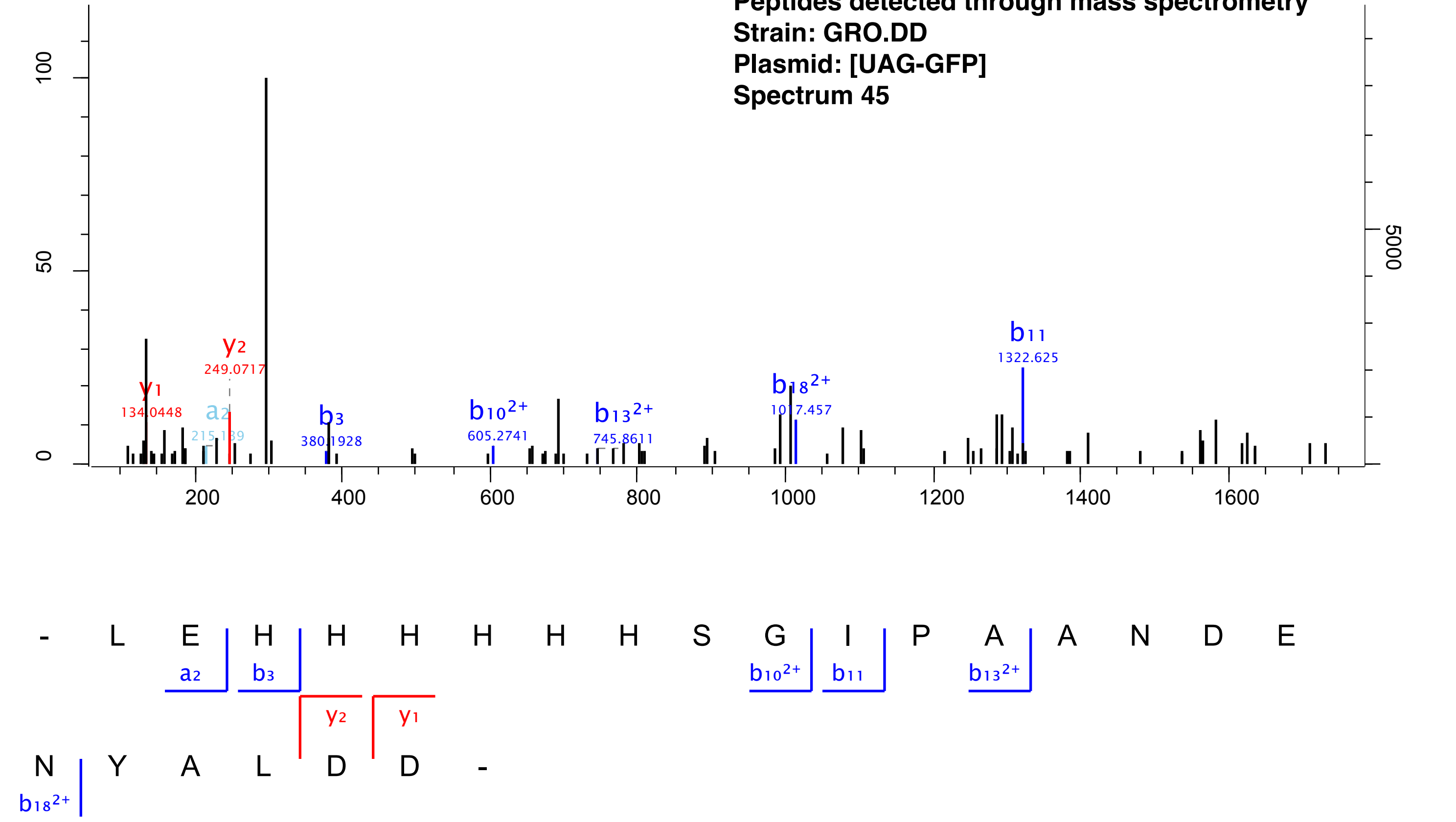

Raw file	Scan	Method	Score	m/z
VOT16-1542	3771	FTMS; HCD	15.27	519.74

Figure 2 – Supplement File 1
Peptides detected through mass spectrometry
Strain: GRO.DD
Plasmid: [UAG-GFP]
Spectrum 46

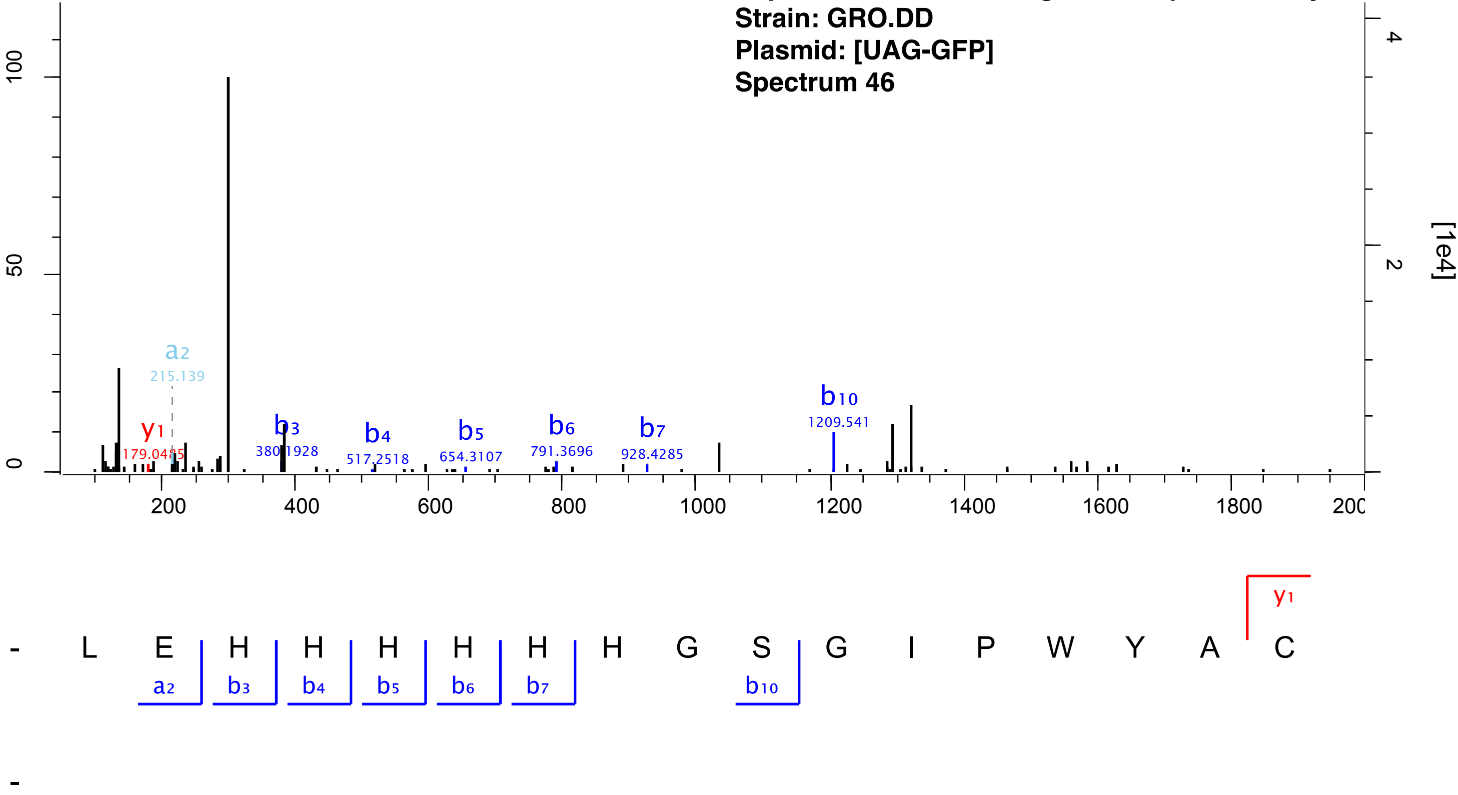

Raw file	Scan	Method	Score	m/z
VOT16-1542	3956	FTMS; HCD	47.04	673.64

Figure 2 – Supplement File 1
Peptides detected through mass spectrometry
Strain: GRO.DD
Plasmid: [UAG-GFP]
Spectrum 47

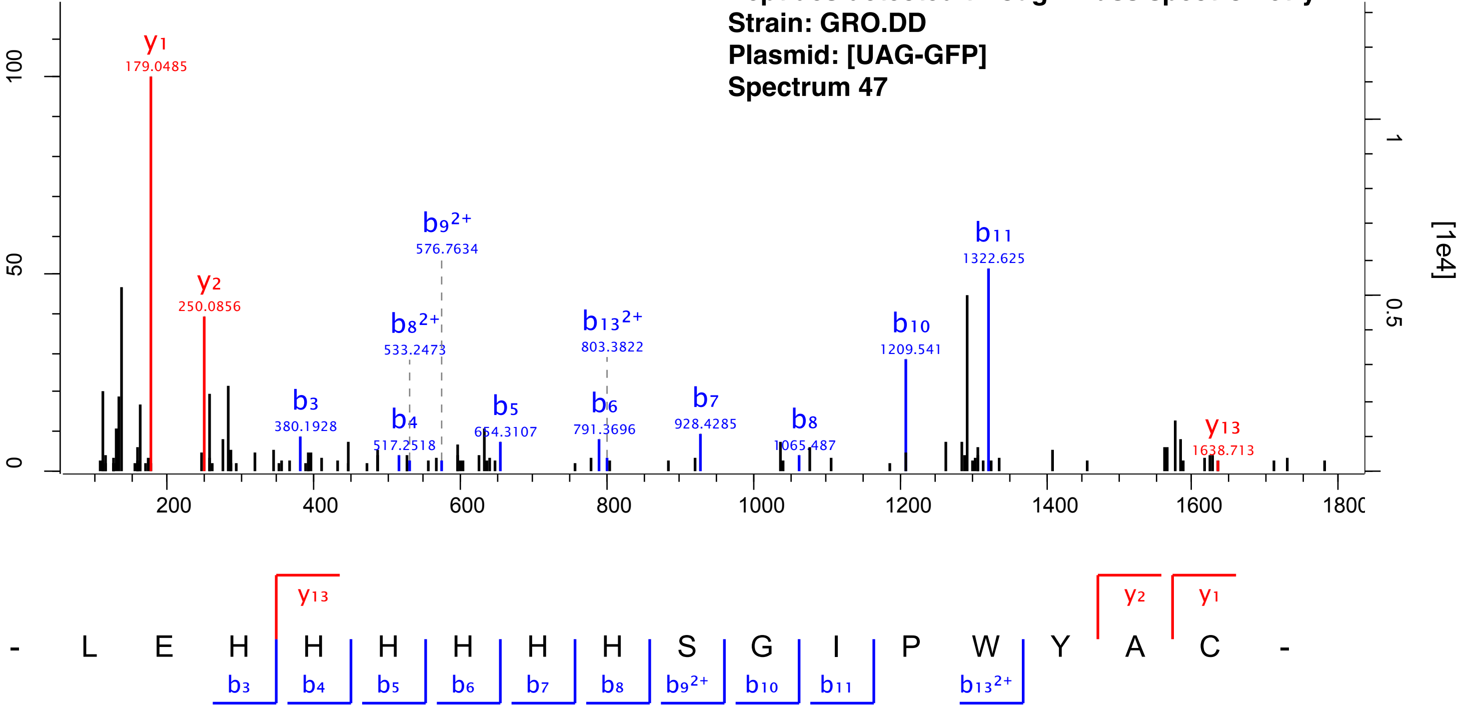

Supplement: Supplementary file 2. [file elife-34878-supp2.pdf]
